# Butler enables rapid cloud-based analysis of thousands of human genomes

**DOI:** 10.1038/s41587-019-0360-3

**Published:** 2020-02-05

**Authors:** Sergei Yakneen, Sebastian M. Waszak, PCAWG Technical Working Group, Brice Aminou, Javier Bartolome, Keith A. Boroevich, Rich Boyce, Angela N. Brooks, Alex Buchanan, Ivo Buchhalter, Adam P. Butler, Niall J. Byrne, Andy Cafferkey, Peter J. Campbell, Zhaohong Chen, Sunghoon Cho, Wan Choi, Peter Clapham, Brandi N. Davis-Dusenbery, Francisco M. De La Vega, Jonas Demeulemeester, Michelle T. Dow, Lewis Jonathan Dursi, Juergen Eils, Roland Eils, Kyle Ellrott, Claudiu Farcas, Francesco Favero, Nodirjon Fayzullaev, Vincent Ferretti, Paul Flicek, Nuno A. Fonseca, Josep Ll. Gelpi, Gad Getz, Bob Gibson, Robert L. Grossman, Olivier Harismendy, Allison P. Heath, Michael C. Heinold, Julian M. Hess, Oliver Hofmann, Jongwhi H. Hong, Thomas J. Hudson, Barbara Hutter, Carolyn M. Hutter, Daniel Hübschmann, Seiya Imoto, Sinisa Ivkovic, Seung-Hyup Jeon, Wei Jiao, Jongsun Jung, Rolf Kabbe, Andre Kahles, Jules N. A. Kerssemakers, Hyung-Lae Kim, Hyunghwan Kim, Jihoon Kim, Youngwook Kim, Kortine Kleinheinz, Michael Koscher, Antonios Koures, Milena Kovacevic, Chris Lawerenz, Ignaty Leshchiner, Jia Liu, Dimitri Livitz, George L. Mihaiescu, Sanja Mijalkovic, Ana Mijalkovic Lazic, Satoru Miyano, Naoki Miyoshi, Hardeep K. Nahal-Bose, Hidewaki Nakagawa, Mia Nastic, Steven J. Newhouse, Jonathan Nicholson, Brian D. O’Connor, David Ocana, Kazuhiro Ohi, Lucila Ohno-Machado, Larsson Omberg, B. F. Francis Ouellette, Nagarajan Paramasivam, Marc D. Perry, Todd D. Pihl, Manuel Prinz, Montserrat Puiggròs, Petar Radovic, Keiran M. Raine, Esther Rheinbay, Mara Rosenberg, Romina Royo, Gunnar Rätsch, Gordon Saksena, Matthias Schlesner, Solomon I. Shorser, Charles Short, Heidi J. Sofia, Jonathan Spring, Lincoln D. Stein, Adam J. Struck, Grace Tiao, Nebojsa Tijanic, David Torrents, Peter Van Loo, Miguel Vazquez, David Vicente, Jeremiah A. Wala, Zhining Wang, Joachim Weischenfeldt, Johannes Werner, Ashley Williams, Youngchoon Woo, Adam J. Wright, Qian Xiang, Liming Yang, Denis Yuen, Christina K. Yung, Junjun Zhang, Michael Gertz, Jan O. Korbel, PCAWG Consortium, Lauri A. Aaltonen, Federico Abascal, Adam Abeshouse, Hiroyuki Aburatani, David J. Adams, Nishant Agrawal, Keun Soo Ahn, Sung-Min Ahn, Hiroshi Aikata, Rehan Akbani, Kadir C. Akdemir, Hikmat Al-Ahmadie, Sultan T. Al-Sedairy, Fatima Al-Shahrour, Malik Alawi, Monique Albert, Kenneth Aldape, Ludmil B. Alexandrov, Adrian Ally, Kathryn Alsop, Eva G. Alvarez, Fernanda Amary, Samirkumar B. Amin, Brice Aminou, Ole Ammerpohl, Matthew J. Anderson, Yeng Ang, Davide Antonello, Pavana Anur, Samuel Aparicio, Elizabeth L. Appelbaum, Yasuhito Arai, Axel Aretz, Koji Arihiro, Shun-ichi Ariizumi, Joshua Armenia, Laurent Arnould, Sylvia Asa, Yassen Assenov, Gurnit Atwal, Sietse Aukema, J. Todd Auman, Miriam R. R. Aure, Philip Awadalla, Marta Aymerich, Gary D. Bader, Adrian Baez-Ortega, Matthew H. Bailey, Peter J. Bailey, Miruna Balasundaram, Saianand Balu, Pratiti Bandopadhayay, Rosamonde E. Banks, Stefano Barbi, Andrew P. Barbour, Jonathan Barenboim, Jill Barnholtz-Sloan, Hugh Barr, Elisabet Barrera, John Bartlett, Javier Bartolome, Claudio Bassi, Oliver F. Bathe, Daniel Baumhoer, Prashant Bavi, Stephen B. Baylin, Wojciech Bazant, Duncan Beardsmore, Timothy A. Beck, Sam Behjati, Andreas Behren, Beifang Niu, Cindy Bell, Sergi Beltran, Christopher Benz, Andrew Berchuck, Anke K. Bergmann, Erik N. Bergstrom, Benjamin P. Berman, Daniel M. Berney, Stephan H. Bernhart, Rameen Beroukhim, Mario Berrios, Samantha Bersani, Johanna Bertl, Miguel Betancourt, Vinayak Bhandari, Shriram G. Bhosle, Andrew V. Biankin, Matthias Bieg, Darell Bigner, Hans Binder, Ewan Birney, Michael Birrer, Nidhan K. Biswas, Bodil Bjerkehagen, Tom Bodenheimer, Lori Boice, Giada Bonizzato, Johann S. De Bono, Arnoud Boot, Moiz S. Bootwalla, Ake Borg, Arndt Borkhardt, Keith A. Boroevich, Ivan Borozan, Christoph Borst, Marcus Bosenberg, Mattia Bosio, Jacqueline Boultwood, Guillaume Bourque, Paul C. Boutros, G. Steven Bova, David T. Bowen, Reanne Bowlby, David D. L. Bowtell, Sandrine Boyault, Rich Boyce, Jeffrey Boyd, Alvis Brazma, Paul Brennan, Daniel S. Brewer, Arie B. Brinkman, Robert G. Bristow, Russell R. Broaddus, Jane E. Brock, Malcolm Brock, Annegien Broeks, Angela N. Brooks, Denise Brooks, Benedikt Brors, Søren Brunak, Timothy J. C. Bruxner, Alicia L. Bruzos, Alex Buchanan, Ivo Buchhalter, Christiane Buchholz, Susan Bullman, Hazel Burke, Birgit Burkhardt, Kathleen H. Burns, John Busanovich, Carlos D. Bustamante, Adam P. Butler, Atul J. Butte, Niall J. Byrne, Anne-Lise Børresen-Dale, Samantha J. Caesar-Johnson, Andy Cafferkey, Declan Cahill, Claudia Calabrese, Carlos Caldas, Fabien Calvo, Niedzica Camacho, Peter J. Campbell, Elias Campo, Cinzia Cantù, Shaolong Cao, Thomas E. Carey, Joana Carlevaro-Fita, Rebecca Carlsen, Ivana Cataldo, Mario Cazzola, Jonathan Cebon, Robert Cerfolio, Dianne E. Chadwick, Dimple Chakravarty, Don Chalmers, Calvin Wing Yiu Chan, Kin Chan, Michelle Chan-Seng-Yue, Vishal S. Chandan, David K. Chang, Stephen J. Chanock, Lorraine A. Chantrill, Aurélien Chateigner, Nilanjan Chatterjee, Kazuaki Chayama, Hsiao-Wei Chen, Jieming Chen, Ken Chen, Yiwen Chen, Zhaohong Chen, Andrew D. Cherniack, Jeremy Chien, Yoke-Eng Chiew, Suet-Feung Chin, Juok Cho, Sunghoon Cho, Jung Kyoon Choi, Wan Choi, Christine Chomienne, Zechen Chong, Su Pin Choo, Angela Chou, Angelika N. Christ, Elizabeth L. Christie, Eric Chuah, Carrie Cibulskis, Kristian Cibulskis, Sara Cingarlini, Peter Clapham, Alexander Claviez, Sean Cleary, Nicole Cloonan, Marek Cmero, Colin C. Collins, Ashton A. Connor, Susanna L. Cooke, Colin S. Cooper, Leslie Cope, Vincenzo Corbo, Matthew G. Cordes, Stephen M. Cordner, Isidro Cortés-Ciriano, Kyle Covington, Prue A. Cowin, Brian Craft, David Craft, Chad J. Creighton, Yupeng Cun, Erin Curley, Ioana Cutcutache, Karolina Czajka, Bogdan Czerniak, Rebecca A. Dagg, Ludmila Danilova, Maria Vittoria Davi, Natalie R. Davidson, Helen Davies, Ian J. Davis, Brandi N. Davis-Dusenbery, Kevin J. Dawson, Francisco M. De La Vega, Ricardo De Paoli-Iseppi, Timothy Defreitas, Angelo P. Dei Tos, Olivier Delaneau, John A. Demchok, Jonas Demeulemeester, German M. Demidov, Deniz Demircioğlu, Nening M. Dennis, Robert E. Denroche, Stefan C. Dentro, Nikita Desai, Vikram Deshpande, Amit G. Deshwar, Christine Desmedt, Jordi Deu-Pons, Noreen Dhalla, Neesha C. Dhani, Priyanka Dhingra, Rajiv Dhir, Anthony DiBiase, Klev Diamanti, Li Ding, Shuai Ding, Huy Q. Dinh, Luc Dirix, HarshaVardhan Doddapaneni, Nilgun Donmez, Michelle T. Dow, Ronny Drapkin, Oliver Drechsel, Ruben M. Drews, Serge Serge, Tim Dudderidge, Ana Dueso-Barroso, Andrew J. Dunford, Michael Dunn, Lewis Jonathan Dursi, Fraser R. Duthie, Ken Dutton-Regester, Jenna Eagles, Douglas F. Easton, Stuart Edmonds, Paul A. Edwards, Sandra E. Edwards, Rosalind A. Eeles, Anna Ehinger, Juergen Eils, Roland Eils, Adel El-Naggar, Matthew Eldridge, Kyle Ellrott, Serap Erkek, Georgia Escaramis, Shadrielle M. G. Espiritu, Xavier Estivill, Dariush Etemadmoghadam, Jorunn E. Eyfjord, Bishoy M. Faltas, Daiming Fan, Yu Fan, William C. Faquin, Claudiu Farcas, Matteo Fassan, Aquila Fatima, Francesco Favero, Nodirjon Fayzullaev, Ina Felau, Sian Fereday, Martin L. Ferguson, Vincent Ferretti, Lars Feuerbach, Matthew A. Field, J. Lynn Fink, Gaetano Finocchiaro, Cyril Fisher, Matthew W. Fittall, Anna Fitzgerald, Rebecca C. Fitzgerald, Adrienne M. Flanagan, Neil E. Fleshner, Paul Flicek, John A. Foekens, Kwun M. Fong, Nuno A. Fonseca, Christopher S. Foster, Natalie S. Fox, Michael Fraser, Scott Frazer, Milana Frenkel-Morgenstern, William Friedman, Joan Frigola, Catrina C. Fronick, Akihiro Fujimoto, Masashi Fujita, Masashi Fukayama, Lucinda A. Fulton, Robert S. Fulton, Mayuko Furuta, P. Andrew Futreal, Anja Füllgrabe, Stacey B. Gabriel, Steven Gallinger, Carlo Gambacorti-Passerini, Jianjiong Gao, Shengjie Gao, Levi Garraway, Øystein Garred, Erik Garrison, Dale W. Garsed, Nils Gehlenborg, Josep L. L. Gelpi, Joshy George, Daniela S. Gerhard, Clarissa Gerhauser, Jeffrey E. Gershenwald, Mark Gerstein, Moritz Gerstung, Gad Getz, Mohammed Ghori, Ronald Ghossein, Nasra H. Giama, Richard A. Gibbs, Bob Gibson, Anthony J. Gill, Pelvender Gill, Dilip D. Giri, Dominik Glodzik, Vincent J. Gnanapragasam, Maria Elisabeth Goebler, Mary J. Goldman, Carmen Gomez, Santiago Gonzalez, Abel Gonzalez-Perez, Dmitry A. Gordenin, James Gossage, Kunihito Gotoh, Ramaswamy Govindan, Dorthe Grabau, Janet S. Graham, Robert C. Grant, Anthony R. Green, Eric Green, Liliana Greger, Nicola Grehan, Sonia Grimaldi, Sean M. Grimmond, Robert L. Grossman, Adam Grundhoff, Gunes Gundem, Qianyun Guo, Manaswi Gupta, Shailja Gupta, Ivo G. Gut, Marta Gut, Jonathan Göke, Gavin Ha, Andrea Haake, David Haan, Siegfried Haas, Kerstin Haase, James E. Haber, Nina Habermann, Faraz Hach, Syed Haider, Natsuko Hama, Freddie C. Hamdy, Anne Hamilton, Mark P. Hamilton, Leng Han, George B. Hanna, Martin Hansmann, Nicholas J. Haradhvala, Olivier Harismendy, Ivon Harliwong, Arif O. Harmanci, Eoghan Harrington, Takanori Hasegawa, David Haussler, Steve Hawkins, Shinya Hayami, Shuto Hayashi, D. Neil Hayes, Stephen J. Hayes, Nicholas K. Hayward, Steven Hazell, Yao He, Allison P. Heath, Simon C. Heath, David Hedley, Apurva M. Hegde, David I. Heiman, Michael C. Heinold, Zachary Heins, Lawrence E. Heisler, Eva Hellstrom-Lindberg, Mohamed Helmy, Seong Gu Heo, Austin J. Hepperla, José María Heredia-Genestar, Carl Herrmann, Peter Hersey, Julian M. Hess, Holmfridur Hilmarsdottir, Jonathan Hinton, Satoshi Hirano, Nobuyoshi Hiraoka, Katherine A. Hoadley, Asger Hobolth, Ermin Hodzic, Jessica I. Hoell, Steve Hoffmann, Oliver Hofmann, Andrea Holbrook, Aliaksei Z. Holik, Michael A. Hollingsworth, Oliver Holmes, Robert A. Holt, Chen Hong, Eun Pyo Hong, Jongwhi H. Hong, Gerrit K. Hooijer, Henrik Hornshøj, Fumie Hosoda, Yong Hou, Volker Hovestadt, William Howat, Alan P. Hoyle, Ralph H. Hruban, Jianhong Hu, Taobo Hu, Xing Hua, Kuan-lin Huang, Mei Huang, Mi Ni Huang, Vincent Huang, Yi Huang, Wolfgang Huber, Thomas J. Hudson, Michael Hummel, Jillian A. Hung, David Huntsman, Ted R. Hupp, Jason Huse, Matthew R. Huska, Barbara Hutter, Carolyn M. Hutter, Daniel Hübschmann, Christine A. Iacobuzio-Donahue, Charles David Imbusch, Marcin Imielinski, Seiya Imoto, William B. Isaacs, Keren Isaev, Shumpei Ishikawa, Murat Iskar, S. M. Ashiqul Islam, Michael Ittmann, Sinisa Ivkovic, Jose M. G. Izarzugaza, Jocelyne Jacquemier, Valerie Jakrot, Nigel B. Jamieson, Gun Ho Jang, Se Jin Jang, Joy C. Jayaseelan, Reyka Jayasinghe, Stuart R. Jefferys, Karine Jegalian, Jennifer L. Jennings, Seung-Hyup Jeon, Lara Jerman, Yuan Ji, Wei Jiao, Peter A. Johansson, Amber L. Johns, Jeremy Johns, Rory Johnson, Todd A. Johnson, Clemency Jolly, Yann Joly, Jon G. Jonasson, Corbin D. Jones, David R. Jones, David T. W. Jones, Nic Jones, Steven J. M. Jones, Jos Jonkers, Young Seok Ju, Hartmut Juhl, Jongsun Jung, Malene Juul, Randi Istrup Juul, Sissel Juul, Natalie Jäger, Rolf Kabbe, Andre Kahles, Abdullah Kahraman, Vera B. Kaiser, Hojabr Kakavand, Sangeetha Kalimuthu, Christof von Kalle, Koo Jeong Kang, Katalin Karaszi, Beth Karlan, Rosa Karlić, Dennis Karsch, Katayoon Kasaian, Karin S. Kassahn, Hitoshi Katai, Mamoru Kato, Hiroto Katoh, Yoshiiku Kawakami, Jonathan D. Kay, Stephen H. Kazakoff, Marat D. Kazanov, Maria Keays, Electron Kebebew, Richard F. Kefford, Manolis Kellis, James G. Kench, Catherine J. Kennedy, Jules N. A. Kerssemakers, David Khoo, Vincent Khoo, Narong Khuntikeo, Ekta Khurana, Helena Kilpinen, Hark Kyun Kim, Hyung-Lae Kim, Hyung-Yong Kim, Hyunghwan Kim, Jaegil Kim, Jihoon Kim, Jong K. Kim, Youngwook Kim, Tari A. King, Wolfram Klapper, Kortine Kleinheinz, Leszek J. Klimczak, Stian Knappskog, Michael Kneba, Bartha M. Knoppers, Youngil Koh, Jan Komorowski, Daisuke Komura, Mitsuhiro Komura, Gu Kong, Marcel Kool, Viktoriya Korchina, Andrey Korshunov, Michael Koscher, Roelof Koster, Zsofia Kote-Jarai, Antonios Koures, Milena Kovacevic, Barbara Kremeyer, Helene Kretzmer, Markus Kreuz, Savitri Krishnamurthy, Dieter Kube, Kiran Kumar, Pardeep Kumar, Sushant Kumar, Yogesh Kumar, Ritika Kundra, Kirsten Kübler, Ralf Küppers, Jesper Lagergren, Phillip H. Lai, Peter W. Laird, Sunil R. Lakhani, Christopher M. Lalansingh, Emilie Lalonde, Fabien C. Lamaze, Adam Lambert, Eric Lander, Pablo Landgraf, Luca Landoni, Anita Langerød, Andrés Lanzós, Denis Larsimont, Erik Larsson, Mark Lathrop, Loretta M. S. Lau, Chris Lawerenz, Rita T. Lawlor, Michael S. Lawrence, Alexander J. Lazar, Ana Mijalkovic Lazic, Xuan Le, Darlene Lee, Donghoon Lee, Eunjung Alice Lee, Hee Jin Lee, Jake June-Koo Lee, Jeong-Yeon Lee, Juhee Lee, Ming Ta Michael Lee, Henry Lee-Six, Kjong-Van Lehmann, Hans Lehrach, Dido Lenze, Conrad R. Leonard, Daniel A. Leongamornlert, Ignaty Leshchiner, Louis Letourneau, Ivica Letunic, Douglas A. Levine, Lora Lewis, Tim Ley, Chang Li, Constance H. Li, Haiyan Irene Li, Jun Li, Lin Li, Shantao Li, Siliang Li, Xiaobo Li, Xiaotong Li, Xinyue Li, Yilong Li, Han Liang, Sheng-Ben Liang, Peter Lichter, Pei Lin, Ziao Lin, W. M. Linehan, Ole Christian Lingjærde, Dongbing Liu, Eric Minwei Liu, Fei-Fei Fei Liu, Fenglin Liu, Jia Liu, Xingmin Liu, Julie Livingstone, Dimitri Livitz, Naomi Livni, Lucas Lochovsky, Markus Loeffler, Georgina V. Long, Armando Lopez-Guillermo, Shaoke Lou, David N. Louis, Laurence B. Lovat, Yiling Lu, Yong-Jie Lu, Youyong Lu, Claudio Luchini, Ilinca Lungu, Xuemei Luo, Hayley J. Luxton, Andy G. Lynch, Lisa Lype, Cristina López, Carlos López-Otín, Eric Z. Ma, Yussanne Ma, Gaetan MacGrogan, Shona MacRae, Geoff Macintyre, Tobias Madsen, Kazuhiro Maejima, Andrea Mafficini, Dennis T. Maglinte, Arindam Maitra, Partha P. Majumder, Luca Malcovati, Salem Malikic, Giuseppe Malleo, Graham J. Mann, Luisa Mantovani-Löffler, Kathleen Marchal, Giovanni Marchegiani, Elaine R. Mardis, Adam A. Margolin, Maximillian G. Marin, Florian Markowetz, Julia Markowski, Jeffrey Marks, Tomas Marques-Bonet, Marco A. Marra, Luke Marsden, John W. M. Martens, Sancha Martin, Jose I. Martin-Subero, Iñigo Martincorena, Alexander Martinez-Fundichely, Yosef E. Maruvka, R. Jay Mashl, Charlie E. Massie, Thomas J. Matthew, Lucy Matthews, Erik Mayer, Simon Mayes, Michael Mayo, Faridah Mbabaali, Karen McCune, Ultan McDermott, Patrick D. McGillivray, Michael D. McLellan, John D. McPherson, John R. McPherson, Treasa A. McPherson, Samuel R. Meier, Alice Meng, Shaowu Meng, Andrew Menzies, Neil D. Merrett, Sue Merson, Matthew Meyerson, William Meyerson, Piotr A. Mieczkowski, George L. Mihaiescu, Sanja Mijalkovic, Tom Mikkelsen, Michele Milella, Linda Mileshkin, Christopher A. Miller, David K. Miller, Jessica K. Miller, Gordon B. Mills, Ana Milovanovic, Sarah Minner, Marco Miotto, Gisela Mir Arnau, Lisa Mirabello, Chris Mitchell, Thomas J. Mitchell, Satoru Miyano, Naoki Miyoshi, Shinichi Mizuno, Fruzsina Molnár-Gábor, Malcolm J. Moore, Richard A. Moore, Sandro Morganella, Quaid D. Morris, Carl Morrison, Lisle E. Mose, Catherine D. Moser, Ferran Muiños, Loris Mularoni, Andrew J. Mungall, Karen Mungall, Elizabeth A. Musgrove, Ville Mustonen, David Mutch, Francesc Muyas, Donna M. Muzny, Alfonso Muñoz, Jerome Myers, Ola Myklebost, Peter Möller, Genta Nagae, Adnan M. Nagrial, Hardeep K. Nahal-Bose, Hitoshi Nakagama, Hidewaki Nakagawa, Hiromi Nakamura, Toru Nakamura, Kaoru Nakano, Tannistha Nandi, Jyoti Nangalia, Mia Nastic, Arcadi Navarro, Fabio C. P. Navarro, David E. Neal, Gerd Nettekoven, Felicity Newell, Steven J. Newhouse, Yulia Newton, Alvin Wei Tian Ng, Anthony Ng, Jonathan Nicholson, David Nicol, Yongzhan Nie, G. Petur Nielsen, Morten Muhlig Nielsen, Serena Nik-Zainal, Michael S. Noble, Katia Nones, Paul A. Northcott, Faiyaz Notta, Brian D. O’Connor, Peter O’Donnell, Maria O’Donovan, Sarah O’Meara, Brian Patrick O’Neill, J. Robert O’Neill, David Ocana, Angelica Ochoa, Layla Oesper, Christopher Ogden, Hideki Ohdan, Kazuhiro Ohi, Lucila Ohno-Machado, Karin A. Oien, Akinyemi I. Ojesina, Hidenori Ojima, Takuji Okusaka, Larsson Omberg, Choon Kiat Ong, Stephan Ossowski, German Ott, B. F. Francis Ouellette, Christine P’ng, Marta Paczkowska, Salvatore Paiella, Chawalit Pairojkul, Marina Pajic, Qiang Pan-Hammarström, Elli Papaemmanuil, Irene Papatheodorou, Nagarajan Paramasivam, Ji Wan Park, Joong-Won Park, Keunchil Park, Kiejung Park, Peter J. Park, Joel S. Parker, Simon L. Parsons, Harvey Pass, Danielle Pasternack, Alessandro Pastore, Ann-Marie Patch, Iris Pauporté, Antonio Pea, John V. Pearson, Chandra Sekhar Pedamallu, Jakob Skou Pedersen, Paolo Pederzoli, Martin Peifer, Nathan A. Pennell, Charles M. Perou, Marc D. Perry, Gloria M. Petersen, Myron Peto, Nicholas Petrelli, Robert Petryszak, Stefan M. Pfister, Mark Phillips, Oriol Pich, Hilda A. Pickett, Todd D. Pihl, Nischalan Pillay, Sarah Pinder, Mark Pinese, Andreia V. Pinho, Esa Pitkänen, Xavier Pivot, Elena Piñeiro-Yáñez, Laura Planko, Christoph Plass, Paz Polak, Tirso Pons, Irinel Popescu, Olga Potapova, Aparna Prasad, Shaun R. Preston, Manuel Prinz, Antonia L. Pritchard, Stephenie D. Prokopec, Elena Provenzano, Xose S. Puente, Sonia Puig, Montserrat Puiggròs, Sergio Pulido-Tamayo, Gulietta M. Pupo, Colin A. Purdie, Michael C. Quinn, Raquel Rabionet, Janet S. Rader, Bernhard Radlwimmer, Petar Radovic, Benjamin Raeder, Keiran M. Raine, Manasa Ramakrishna, Kamna Ramakrishnan, Suresh Ramalingam, Benjamin J. Raphael, W. Kimryn Rathmell, Tobias Rausch, Guido Reifenberger, Jüri Reimand, Jorge Reis-Filho, Victor Reuter, Iker Reyes-Salazar, Matthew A. Reyna, Sheila M. Reynolds, Esther Rheinbay, Yasser Riazalhosseini, Andrea L. Richardson, Julia Richter, Matthew Ringel, Markus Ringnér, Yasushi Rino, Karsten Rippe, Jeffrey Roach, Lewis R. Roberts, Nicola D. Roberts, Steven A. Roberts, A. Gordon Robertson, Alan J. Robertson, Javier Bartolomé Rodriguez, Bernardo Rodriguez-Martin, F. Germán Rodríguez-González, Michael H. A. Roehrl, Marius Rohde, Hirofumi Rokutan, Gilles Romieu, Ilse Rooman, Tom Roques, Daniel Rosebrock, Mara Rosenberg, Philip C. Rosenstiel, Andreas Rosenwald, Edward W. Rowe, Romina Royo, Steven G. Rozen, Yulia Rubanova, Mark A. Rubin, Carlota Rubio-Perez, Vasilisa A. Rudneva, Borislav C. Rusev, Andrea Ruzzenente, Gunnar Rätsch, Radhakrishnan Sabarinathan, Veronica Y. Sabelnykova, Sara Sadeghi, S. Cenk Sahinalp, Natalie Saini, Mihoko Saito-Adachi, Gordon Saksena, Adriana Salcedo, Roberto Salgado, Leonidas Salichos, Richard Sallari, Charles Saller, Roberto Salvia, Michelle Sam, Jaswinder S. Samra, Francisco Sanchez-Vega, Chris Sander, Grant Sanders, Rajiv Sarin, Iman Sarrafi, Aya Sasaki-Oku, Torill Sauer, Guido Sauter, Robyn P. M. Saw, Maria Scardoni, Christopher J. Scarlett, Aldo Scarpa, Ghislaine Scelo, Dirk Schadendorf, Jacqueline E. Schein, Markus B. Schilhabel, Matthias Schlesner, Thorsten Schlomm, Heather K. Schmidt, Sarah-Jane Schramm, Stefan Schreiber, Nikolaus Schultz, Steven E. Schumacher, Roland F. Schwarz, Richard A. Scolyer, David Scott, Ralph Scully, Raja Seethala, Ayellet V. Segre, Iris Selander, Colin A. Semple, Yasin Senbabaoglu, Subhajit Sengupta, Elisabetta Sereni, Stefano Serra, Dennis C. Sgroi, Mark Shackleton, Nimish C. Shah, Sagedeh Shahabi, Catherine A. Shang, Ping Shang, Ofer Shapira, Troy Shelton, Ciyue Shen, Hui Shen, Rebecca Shepherd, Ruian Shi, Yan Shi, Yu-Jia Shiah, Tatsuhiro Shibata, Juliann Shih, Eigo Shimizu, Kiyo Shimizu, Seung Jun Shin, Yuichi Shiraishi, Tal Shmaya, Ilya Shmulevich, Solomon I. Shorser, Charles Short, Raunak Shrestha, Suyash S. Shringarpure, Craig Shriver, Shimin Shuai, Nikos Sidiropoulos, Reiner Siebert, Anieta M. Sieuwerts, Lina Sieverling, Sabina Signoretti, Katarzyna O. Sikora, Michele Simbolo, Ronald Simon, Janae V. Simons, Jared T. Simpson, Peter T. Simpson, Samuel Singer, Nasa Sinnott-Armstrong, Payal Sipahimalani, Tara J. Skelly, Marcel Smid, Jaclyn Smith, Karen Smith-McCune, Nicholas D. Socci, Heidi J. Sofia, Matthew G. Soloway, Lei Song, Anil K. Sood, Sharmila Sothi, Christos Sotiriou, Cameron M. Soulette, Paul N. Span, Paul T. Spellman, Nicola Sperandio, Andrew J. Spillane, Oliver Spiro, Jonathan Spring, Johan Staaf, Peter F. Stadler, Peter Staib, Stefan G. Stark, Lucy Stebbings, Ólafur Andri Stefánsson, Oliver Stegle, Lincoln D. Stein, Alasdair Stenhouse, Chip Stewart, Stephan Stilgenbauer, Miranda D. Stobbe, Michael R. Stratton, Jonathan R. Stretch, Adam J. Struck, Joshua M. Stuart, Henk G. Stunnenberg, Hong Su, Xiaoping Su, Ren X. Sun, Stephanie Sungalee, Hana Susak, Akihiro Suzuki, Fred Sweep, Monika Szczepanowski, Holger Sültmann, Takashi Yugawa, Angela Tam, David Tamborero, Benita Kiat Tee Tan, Donghui Tan, Patrick Tan, Hiroko Tanaka, Hirokazu Taniguchi, Tomas J. Tanskanen, Maxime Tarabichi, Roy Tarnuzzer, Patrick Tarpey, Morgan L. Taschuk, Kenji Tatsuno, Simon Tavaré, Darrin F. Taylor, Amaro Taylor-Weiner, Jon W. Teague, Bin Tean Teh, Varsha Tembe, Javier Temes, Kevin Thai, Sarah P. Thayer, Nina Thiessen, Gilles Thomas, Sarah Thomas, Alan Thompson, Alastair M. Thompson, John F. F. Thompson, R. Houston Thompson, Heather Thorne, Leigh B. Thorne, Adrian Thorogood, Grace Tiao, Nebojsa Tijanic, Lee E. Timms, Roberto Tirabosco, Marta Tojo, Stefania Tommasi, Christopher W. Toon, Umut H. Toprak, David Torrents, Giampaolo Tortora, Jörg Tost, Yasushi Totoki, David Townend, Nadia Traficante, Isabelle Treilleux, Jean-Rémi Trotta, Lorenz H. P. Trümper, Ming Tsao, Tatsuhiko Tsunoda, Jose M. C. Tubio, Olga Tucker, Richard Turkington, Daniel J. Turner, Andrew Tutt, Masaki Ueno, Naoto T. Ueno, Christopher Umbricht, Husen M. Umer, Timothy J. Underwood, Lara Urban, Tomoko Urushidate, Tetsuo Ushiku, Liis Uusküla-Reimand, Alfonso Valencia, David J. Van Den Berg, Steven Van Laere, Peter Van Loo, Erwin G. Van Meir, Gert G. Van den Eynden, Theodorus Van der Kwast, Naveen Vasudev, Miguel Vazquez, Ravikiran Vedururu, Umadevi Veluvolu, Shankar Vembu, Lieven P. C. Verbeke, Peter Vermeulen, Clare Verrill, Alain Viari, David Vicente, Caterina Vicentini, K. VijayRaghavan, Juris Viksna, Ricardo E. Vilain, Izar Villasante, Anne Vincent-Salomon, Tapio Visakorpi, Douglas Voet, Paresh Vyas, Ignacio Vázquez-García, Nick M. Waddell, Nicola Waddell, Claes Wadelius, Lina Wadi, Rabea Wagener, Jeremiah A. Wala, Jian Wang, Jiayin Wang, Linghua Wang, Qi Wang, Wenyi Wang, Yumeng Wang, Zhining Wang, Paul M. Waring, Hans-Jörg Warnatz, Jonathan Warrell, Anne Y. Warren, David C. Wedge, Dieter Weichenhan, Paul Weinberger, John N. Weinstein, Joachim Weischenfeldt, Daniel J. Weisenberger, Ian Welch, Michael C. Wendl, Johannes Werner, Justin P. Whalley, David A. Wheeler, Hayley C. Whitaker, Dennis Wigle, Matthew D. Wilkerson, Ashley Williams, James S. Wilmott, Gavin W. Wilson, Julie M. Wilson, Richard K. Wilson, Boris Winterhoff, Jeffrey A. Wintersinger, Maciej Wiznerowicz, Stephan Wolf, Bernice H. Wong, Tina Wong, Winghing Wong, Youngchoon Woo, Scott Wood, Bradly G. Wouters, Adam J. Wright, Derek W. Wright, Mark H. Wright, Chin-Lee Wu, Dai-Ying Wu, Guanming Wu, Jianmin Wu, Kui Wu, Yang Wu, Zhenggang Wu, Liu Xi, Tian Xia, Qian Xiang, Xiao Xiao, Rui Xing, Heng Xiong, Qinying Xu, Yanxun Xu, Hong Xue, Shinichi Yachida, Rui Yamaguchi, Takafumi N. Yamaguchi, Masakazu Yamamoto, Shogo Yamamoto, Hiroki Yamaue, Fan Yang, Huanming Yang, Jean Y. Yang, Liming Yang, Lixing Yang, Shanlin Yang, Tsun-Po Yang, Yang Yang, Xiaotong Yao, Marie-Laure Yaspo, Lucy Yates, Christina Yau, Chen Ye, Kai Ye, Venkata D. Yellapantula, Christopher J. Yoon, Sung-Soo Yoon, Fouad Yousif, Jun Yu, Kaixian Yu, Willie Yu, Yingyan Yu, Ke Yuan, Yuan Yuan, Denis Yuen, Olga Zaikova, Jorge Zamora, Marc Zapatka, Jean C. Zenklusen, Thorsten Zenz, Nikolajs Zeps, Cheng-Zhong Zhang, Fan Zhang, Hailei Zhang, Hongwei Zhang, Hongxin Zhang, Jiashan Zhang, Jing Zhang, Junjun Zhang, Xiuqing Zhang, Xuanping Zhang, Yan Zhang, Zemin Zhang, Zhongming Zhao, Liangtao Zheng, Xiuqing Zheng, Wanding Zhou, Yong Zhou, Bin Zhu, Hongtu Zhu, Jingchun Zhu, Shida Zhu, Lihua Zou, Xueqing Zou, Anna deFazio, Nicholas van As, Carolien H. M. van Deurzen, Marc J. van de Vijver, L. van’t Veer, Christian von Mering

**Affiliations:** 1 European Molecular Biology Laboratory (EMBL), Genome Biology Unit, Heidelberg, Germany; 2 Institute of Computer Science, Heidelberg University, Heidelberg, Germany; 4 EMBL, European Bioinformatics Institute (EMBL-EBI), Hinxton, UK; 6 Present Address: Sophia Genetics SA, Saint Sulpice, Switzerland; 7 Genome Informatics Program, Ontario Institute for Cancer Research, Toronto, Ontario Canada; 8 Barcelona Supercomputing Center (BSC), Barcelona, Spain; 9 Laboratory for Medical Science Mathematics, RIKEN Center for Integrative Medical Sciences, Yokohama, Kanagawa Japan; 10 RIKEN Center for Integrative Medical Sciences, Yokohama, Kanagawa Japan; 11 Broad Institute of MIT and Harvard, Cambridge, MA USA; 12 Department of Medical Oncology, Dana-Farber Cancer Institute, Boston, MA USA; 13 Department of Biomolecular Engineering, University of California Santa Cruz, Santa Cruz, CA USA; 14 UC Santa Cruz Genomics Institute, University of California Santa Cruz, Santa Cruz, CA USA; 15 Biomedical Engineering, Oregon Health and Science University, Portland, OR USA; 16 Division of Theoretical Bioinformatics, German Cancer Research Center (DKFZ), Heidelberg, Germany; 17 Heidelberg Center for Personalized Oncology (DKFZ-HIPO), German Cancer Research Center, Heidelberg, Germany; 18 Institute of Pharmacy and Molecular Biotechnology and BioQuant, Heidelberg University, Heidelberg, Germany; 19 Wellcome Sanger Institute, Wellcome Genome Campus, Hinxton, Cambridge UK; 20 Department of Haematology, University of Cambridge, Cambridge, UK; 21 University of California San Diego, San Diego, CA USA; 22 PDXen Biosystems Inc, Seoul, South Korea; 23 Electronics and Telecommunications Research Institute, Daejeon, South Korea; 24 Seven Bridges Genomics, Charlestown, MA USA; 25 Annai Systems, Inc, Carlsbad, CA USA; 26 Department of Biomedical Data Science, Stanford University School of Medicine, Stanford, CA USA; 27 Department of Genetics, Stanford University School of Medicine, Stanford, CA USA; 28 University of Leuven, Leuven, Belgium; 29 The Francis Crick Institute, London, UK; 30 Computational Biology Program, Ontario Institute for Cancer Research, Toronto, Ontario Canada; 31 The Hospital for Sick Children, Toronto, Ontario Canada; 32 Heidelberg University, Heidelberg, Germany; 33 New BIH Digital Health Center, Berlin Institute of Health (BIH) and Charité – Universitätsmedizin Berlin, Berlin, Germany; 34 Rigshospitalet, Copenhagen, Denmark; 35 Department of Biochemistry and Molecular Medicine, University of Montreal, Montreal, Quebec Canada; 36 CIBIO/InBIO—Research Center in Biodiversity and Genetic Resources, Universidade do Porto, Vairão, Portugal; 37 Department Biochemistry and Molecular Biomedicine, University of Barcelona, Barcelona, Spain; 38 Center for Cancer Research, Massachusetts General Hospital, Boston, MA USA; 39 Department of Pathology, Massachusetts General Hospital, Boston, MA USA; 40 Harvard Medical School, Boston, MA USA; 41 Department of Medicine, Section of Hematology/Oncology, University of Chicago, Chicago, IL USA; 42 Division of Biomedical Informatics, Department of Medicine, & Moores Cancer Center, UC San Diego School of Medicine, San Diego, CA USA; 43 Children’s Hospital of Philadelphia, Philadelphia, PA USA; 44 Massachusetts General Hospital Center for Cancer Research, Charlestown, MA USA; 45 University of Melbourne Centre for Cancer Research, University of Melbourne, Melbourne, Victoria Australia; 46 Syntekabio Inc, Daejeon, South Korea; 47 AbbVie, North Chicago, IL USA; 48 Genomics Program, Ontario Institute for Cancer Research, Toronto, Ontario Canada; 49 German Cancer Consortium (DKTK), Heidelberg, Germany; 50 Heidelberg Center for Personalized Oncology (DKFZ-HIPO), German Cancer Research Center (DKFZ), Heidelberg, Germany; 51 National Center for Tumor Diseases (NCT) Heidelberg, Heidelberg, Germany; 52 National Human Genome Research Institute, National Institutes of Health, Bethesda, MD USA; 53 Department of Pediatric Immunology, Hematology and Oncology, University Hospital, Heidelberg, Germany; 54 German Cancer Research Center (DKFZ), Heidelberg, Germany; 55 Heidelberg Institute for Stem Cell Technology and Experimental Medicine (HI-STEM), Heidelberg, Germany; 56 Institute of Medical Science, University of Tokyo, Tokyo, Japan; 57 Genome Integration Data Center, Syntekabio, Inc, Daejeon, South Korea; 58 Computational Biology Center, Memorial Sloan Kettering Cancer Center, New York, NY USA; 59 ETH Zurich, Department of Biology, Zurich, Switzerland; 60 ETH Zurich, Department of Computer Science, Zurich, Switzerland; 61 SIB Swiss Institute of Bioinformatics, Lausanne, Switzerland; 62 University Hospital Zurich, Zurich, Switzerland; 63 Department of Biochemistry, College of Medicine, Ewha Womans University, Seoul, South Korea; 64 Health Sciences Department of Biomedical Informatics, University of California San Diego, La Jolla, CA USA; 65 Department of Health Sciences and Technology, Sungkyunkwan University School of Medicine, Seoul, South Korea; 66 Samsung Genome Institute, Seoul, South Korea; 67 Functional and Structural Genomics, German Cancer Research Center (DKFZ), Heidelberg, Germany; 68 Leidos Biomedical Research, Inc, McLean, VA USA; 69 Center for Biomolecular Science and Engineering, University of California Santa Cruz, Santa Cruz, CA USA; 70 Sage Bionetworks, Seattle, WA USA; 71 Department of Cell and Systems Biology, University of Toronto, Toronto, Ontario Canada; 72 Department of Radiation Oncology, University of California San Francisco, San Francisco, CA USA; 73 CSRA Incorporated, Fairfax, VA USA; 74 Massachusetts General Hospital, Boston, MA USA; 75 Department of Biology, ETH Zurich, Zurich, Switzerland; 76 Department of Computer Science, ETH Zurich, Zurich, Switzerland; 77 Weill Cornell Medical College, New York, NY USA; 78 Bioinformatics and Omics Data Analytics, German Cancer Research Center (DKFZ), Heidelberg, Germany; 79 Institute for Genomics and Systems Biology, University of Chicago, Chicago, IL USA; 80 Department of Molecular Genetics, University of Toronto, Toronto, Ontario Canada; 81 Computational Biology Program, School of Medicine, Oregon Health and Science University, Portland, OR USA; 82 Institució Catalana de Recerca i Estudis Avançats (ICREA), Barcelona, Spain; 83 Department of Clinical and Molecular Medicine, Faculty of Medicine and Health Sciences, Norwegian University of Science and Technology, Trondheim, Norway; 84 National Cancer Institute, National Institutes of Health, Bethesda, MD USA; 85 Finsen Laboratory and Biotech Research & Innovation Centre (BRIC), University of Copenhagen, Copenhagen, Denmark; 86 Department of Urology, Charité Universitätsmedizin Berlin, Berlin, Germany; 87 Department of Biological Oceanography, Leibniz Institute of Baltic Sea Research, Rostock, Germany; 88 Applied Tumor Genomics Research Program, Research Programs Unit, University of Helsinki, Helsinki, Finland; 89 Wellcome Sanger Institute, Wellcome Genome Campus, Hinxton, UK; 90 Memorial Sloan Kettering Cancer Center, New York, NY USA; 91 Genome Science Division, Research Center for Advanced Science and Technology, University of Tokyo, Tokyo, Japan; 92 Department of Surgery, University of Chicago, Chicago, IL USA; 93 Department of Surgery, Division of Hepatobiliary and Pancreatic Surgery, School of Medicine, Keimyung University Dongsan Medical Center, Daegu, South Korea; 94 Department of Oncology, Gil Medical Center, Gachon University, Incheon, South Korea; 95 Hiroshima University, Hiroshima, Japan; 96 Department of Bioinformatics and Computational Biology, The University of Texas MD Anderson Cancer Center, Houston, TX USA; 97 University of Texas MD Anderson Cancer Center, Houston, TX USA; 98 King Faisal Specialist Hospital and Research Centre, Al Maather, Riyadh, Saudi Arabia; 99 Bioinformatics Unit, Spanish National Cancer Research Centre (CNIO), Madrid, Spain; 100 Bioinformatics Core Facility, University Medical Center Hamburg, Hamburg, Germany; 101 Heinrich Pette Institute, Leibniz Institute for Experimental Virology, Hamburg, Germany; 102 Ontario Tumour Bank, Ontario Institute for Cancer Research, Toronto, ON Canada; 103 Department of Pathology, The University of Texas MD Anderson Cancer Center, Houston, TX USA; 104 Laboratory of Pathology, Center for Cancer Research, National Cancer Institute, Bethesda, MD USA; 105 Department of Cellular and Molecular Medicine and Department of Bioengineering, University of California San Diego, La Jolla, CA USA; 106 UC San Diego Moores Cancer Center, San Diego, CA USA; 107 Canada’s Michael Smith Genome Sciences Centre, BC Cancer, Vancouver, BC Canada; 108 Sir Peter MacCallum Department of Oncology, Peter MacCallum Cancer Centre, University of Melbourne, Melbourne, VIC Australia; 109 Centre for Research in Molecular Medicine and Chronic Diseases (CiMUS), Universidade de Santiago de Compostela, Santiago de Compostela, Spain; 110 Department of Zoology, Genetics and Physical Anthropology, (CiMUS), Universidade de Santiago de Compostela, Santiago de Compostela, Spain; 111 The Biomedical Research Centre (CINBIO), Universidade de Vigo, Vigo, Spain; 112 Royal National Orthopaedic Hospital - Bolsover, London, UK; 113 Department of Genomic Medicine, The University of Texas MD Anderson Cancer Center, Houston, TX USA; 114 Quantitative and Computational Biosciences Graduate Program, Baylor College of Medicine, Houston, TX USA; 115 The Jackson Laboratory for Genomic Medicine, Farmington, CT USA; 116 Genome Informatics Program, Ontario Institute for Cancer Research, Toronto, ON Canada; 117 Institute of Human Genetics, Christian-Albrechts-University, Kiel, Germany; 118 Institute of Human Genetics, Ulm University and Ulm University Medical Center, Ulm, Germany; 119 Queensland Centre for Medical Genomics, Institute for Molecular Bioscience, University of Queensland, St. Lucia, Brisbane, QLD Australia; 120 Salford Royal NHS Foundation Trust, Salford, UK; 121 Department of Surgery, Pancreas Institute, University and Hospital Trust of Verona, Verona, Italy; 122 Molecular and Medical Genetics, OHSU Knight Cancer Institute, Oregon Health and Science University, Portland, OR USA; 123 Department of Molecular Oncology, BC Cancer Research Centre, Vancouver, BC Canada; 124 The McDonnell Genome Institute at Washington University, St. Louis, MO USA; 125 University College London, London, UK; 126 Division of Cancer Genomics, National Cancer Center Research Institute, National Cancer Center, Tokyo, Japan; 127 DLR Project Management Agency, Bonn, Germany; 128 Tokyo Women’s Medical University, Tokyo, Japan; 129 Center for Molecular Oncology, Memorial Sloan Kettering Cancer Center, New York, NY USA; 130 Los Alamos National Laboratory, Los Alamos, NM USA; 131 Department of Pathology, University Health Network, Toronto General Hospital, Toronto, ON Canada; 132 Nottingham University Hospitals NHS Trust, Nottingham, UK; 133 Epigenomics and Cancer Risk Factors, German Cancer Research Center (DKFZ), Heidelberg, Germany; 134 Computational Biology Program, Ontario Institute for Cancer Research, Toronto, ON Canada; 135 Department of Molecular Genetics, University of Toronto, Toronto, ON Canada; 136 Vector Institute, Toronto, ON Canada; 137 Hematopathology Section, Institute of Pathology, Christian-Albrechts-University, Kiel, Germany; 138 Department of Pathology and Laboratory Medicine, School of Medicine, University of North Carolina at Chapel Hill, Chapel Hill, NC USA; 139 Department of Cancer Genetics, Institute for Cancer Research, Oslo University Hospital, The Norwegian Radium Hospital, Oslo, Norway; 140 Pathology, Hospital Clinic, Institut d’Investigacions Biomèdiques August Pi i Sunyer (IDIBAPS), University of Barcelona, Barcelona, Spain; 141 Department of Veterinary Medicine, Transmissible Cancer Group, University of Cambridge, Cambridge, UK; 142 Alvin J. Siteman Cancer Center, Washington University School of Medicine, St. Louis, MO USA; 143 Wolfson Wohl Cancer Research Centre, Institute of Cancer Sciences, University of Glasgow, Glasgow, UK; 144 Lineberger Comprehensive Cancer Center, University of North Carolina at Chapel Hill, Chapel Hill, NC USA; 145 Broad Institute of MIT and Harvard, Cambridge, MA USA; 146 Dana-Farber/Boston Children’s Cancer and Blood Disorders Center, Boston, MA USA; 147 Department of Pediatrics, Harvard Medical School, Boston, MA USA; 148 Leeds Institute of Medical Research @ St. James’s, University of Leeds, St. James’s University Hospital, Leeds, UK; 149 Department of Pathology and Diagnostics, University and Hospital Trust of Verona, Verona, Italy; 150 Department of Surgery, Princess Alexandra Hospital, Brisbane, QLD Australia; 151 Surgical Oncology Group, Diamantina Institute, University of Queensland, Brisbane, QLD Australia; 152 Department of Population and Quantitative Health Sciences, Case Western Reserve University School of Medicine, Cleveland, OH USA; 153 Research Health Analytics and Informatics, University Hospitals Cleveland Medical Center, Cleveland, OH USA; 154 Gloucester Royal Hospital, Gloucester, UK; 155 European Molecular Biology Laboratory, European Bioinformatics Institute (EMBL-EBI), Cambridge, UK; 156 Diagnostic Development, Ontario Institute for Cancer Research, Toronto, ON Canada; 157 Barcelona Supercomputing Center (BSC), Barcelona, Spain; 158 Arnie Charbonneau Cancer Institute, University of Calgary, Calgary, AB Canada; 159 Departments of Surgery and Oncology, University of Calgary, Calgary, AB Canada; 160 Department of Pathology, Oslo University Hospital, The Norwegian Radium Hospital, Oslo, Norway; 161 PanCuRx Translational Research Initiative, Ontario Institute for Cancer Research, Toronto, ON Canada; 162 Department of Oncology, Sidney Kimmel Comprehensive Cancer Center at Johns Hopkins University School of Medicine, Baltimore, MD USA; 163 University Hospital Southampton NHS Foundation Trust, Southampton, UK; 164 Royal Stoke University Hospital, Stoke-on-Trent, UK; 165 Genome Sequence Informatics, Ontario Institute for Cancer Research, Toronto, ON Canada; 166 Human Longevity Inc, San Diego, CA USA; 167 Olivia Newton-John Cancer Research Institute, La Trobe University, Heidelberg, VIC Australia; 168 Computer Network Information Center, Chinese Academy of Sciences, Beijing, China; 169 Genome Canada, Ottawa, ON Canada; 170 CNAG-CRG, Centre for Genomic Regulation (CRG), Barcelona Institute of Science and Technology (BIST), Barcelona, Spain; 171 Universitat Pompeu Fabra (UPF), Barcelona, Spain; 172 Buck Institute for Research on Aging, Novato, CA USA; 173 Duke University Medical Center, Durham, NC USA; 174 Department of Human Genetics, Hannover Medical School, Hannover, Germany; 175 Center for Bioinformatics and Functional Genomics, Cedars-Sinai Medical Center, Los Angeles, CA USA; 176 Department of Biomedical Sciences, Cedars-Sinai Medical Center, Los Angeles, CA USA; 177 The Hebrew University Faculty of Medicine, Jerusalem, Israel; 178 Barts Cancer Institute, Barts and the London School of Medicine and Dentistry, Queen Mary University of London, London, UK; 179 Department of Computer Science, Bioinformatics Group, University of Leipzig, Leipzig, Germany; 180 Interdisciplinary Center for Bioinformatics, University of Leipzig, Leipzig, Germany; 181 Transcriptome Bioinformatics, LIFE Research Center for Civilization Diseases, University of Leipzig, Leipzig, Germany; 182 Department of Medical Oncology, Dana-Farber Cancer Institute, Boston, MA USA; 183 Department of Cancer Biology, Dana-Farber Cancer Institute, Boston, MA USA; 184 Harvard Medical School, Boston, MA USA; 185 USC Norris Comprehensive Cancer Center, University of Southern California, Los Angeles, CA USA; 186 Department of Diagnostics and Public Health, University and Hospital Trust of Verona, Verona, Italy; 187 Department of Mathematics, Aarhus University, Aarhus, Denmark; 188 Department of Molecular Medicine (MOMA), Aarhus University Hospital, Aarhus N, Denmark; 189 Instituto Carlos Slim de la Salud, Mexico City, Mexico; 190 Department of Medical Biophysics, University of Toronto, Toronto, ON Canada; 191 Cancer Division, Garvan Institute of Medical Research, Kinghorn Cancer Centre, University of New South Wales (UNSW Sydney), Sydney, NSW Australia; 192 South Western Sydney Clinical School, Faculty of Medicine, University of New South Wales (UNSW Sydney), Liverpool, NSW Australia; 193 West of Scotland Pancreatic Unit, Glasgow Royal Infirmary, Glasgow, UK; 194 Center for Digital Health, Berlin Institute of Health and Charitè - Universitätsmedizin Berlin, Berlin, Germany; 195 Heidelberg Center for Personalized Oncology (DKFZ-HIPO), German Cancer Research Center (DKFZ), Heidelberg, Germany; 196 The Preston Robert Tisch Brain Tumor Center, Duke University Medical Center, Durham, NC USA; 197 Massachusetts General Hospital, Boston, MA USA; 198 National Institute of Biomedical Genomics, Kalyani, West Bengal India; 199 Institute of Clinical Medicine and Institute of Oral Biology, University of Oslo, Oslo, Norway; 200 University of North Carolina at Chapel Hill, Chapel Hill, NC USA; 201 ARC-Net Centre for Applied Research on Cancer, University and Hospital Trust of Verona, Verona, Italy; 202 The Institute of Cancer Research, London, UK; 203 Centre for Computational Biology, Duke-NUS Medical School, Singapore, Singapore; 204 Programme in Cancer and Stem Cell Biology, Duke-NUS Medical School, Singapore, Singapore; 205 Division of Oncology and Pathology, Department of Clinical Sciences Lund, Lund University, Lund, Sweden; 206 Department of Pediatric Oncology, Hematology and Clinical Immunology, Heinrich-Heine-University, Düsseldorf, Germany; 207 Laboratory for Medical Science Mathematics, RIKEN Center for Integrative Medical Sciences, Yokohama, Japan; 208 RIKEN Center for Integrative Medical Sciences, Yokohama, Japan; 209 Department of Internal Medicine/Hematology, Friedrich-Ebert-Hospital, Neumünster, Germany; 210 Departments of Dermatology and Pathology, Yale University, New Haven, CT USA; 211 Centre for Genomic Regulation (CRG), The Barcelona Institute of Science and Technology, Barcelona, Spain; 212 Radcliffe Department of Medicine, University of Oxford, Oxford, UK; 213 Canadian Center for Computational Genomics, McGill University, Montreal, QC Canada; 214 Department of Human Genetics, McGill University, Montreal, QC Canada; 215 Department of Human Genetics, University of California Los Angeles, Los Angeles, CA USA; 216 Department of Pharmacology, University of Toronto, Toronto, ON Canada; 217 Faculty of Medicine and Health Technology, Tampere University and Tays Cancer Center, Tampere University Hospital, Tampere, Finland; 218 Haematology, Leeds Teaching Hospitals NHS Trust, Leeds, UK; 219 Translational Research and Innovation, Centre Léon Bérard, Lyon, France; 220 Fox Chase Cancer Center, Philadelphia, PA USA; 221 International Agency for Research on Cancer, World Health Organization, Lyon, France; 222 Earlham Institute, Norwich, UK; 223 Norwich Medical School, University of East Anglia, Norwich, UK; 224 Department of Molecular Biology, Faculty of Science, Radboud Institute for Molecular Life Sciences, Radboud University, Nijmegen, HB The Netherlands; 225 CRUK Manchester Institute and Centre, Manchester, UK; 226 Department of Radiation Oncology, University of Toronto, Toronto, ON Canada; 227 Division of Cancer Sciences, Manchester Cancer Research Centre, University of Manchester, Manchester, UK; 228 Radiation Medicine Program, Princess Margaret Cancer Centre, Toronto, ON Canada; 229 Department of Pathology, Brigham and Women’s Hospital, Harvard Medical School, Boston, MA USA; 230 Department of Surgery, Division of Thoracic Surgery, The Johns Hopkins University School of Medicine, Baltimore, MD USA; 231 Division of Molecular Pathology, The Netherlands Cancer Institute, Oncode Institute, Amsterdam, CX The Netherlands; 232 Department of Biomolecular Engineering, University of California Santa Cruz, Santa Cruz, CA USA; 233 UC Santa Cruz Genomics Institute, University of California Santa Cruz, Santa Cruz, CA USA; 234 Division of Applied Bioinformatics, German Cancer Research Center (DKFZ), Heidelberg, Germany; 235 German Cancer Consortium (DKTK), German Cancer Research Center (DKFZ), Heidelberg, Germany; 236 National Center for Tumor Diseases (NCT) Heidelberg, Heidelberg, Germany; 237 Center for Biological Sequence Analysis, Department of Bio and Health Informatics, Technical University of Denmark, Lyngby, Denmark; 238 Novo Nordisk Foundation Center for Protein Research, University of Copenhagen, Copenhagen, Denmark; 239 Institute for Molecular Bioscience, University of Queensland, St. Lucia, Brisbane, QLD Australia; 240 Biomedical Engineering, Oregon Health and Science University, Portland, OR USA; 241 Division of Theoretical Bioinformatics, German Cancer Research Center (DKFZ), Heidelberg, Germany; 242 Institute of Pharmacy and Molecular Biotechnology and BioQuant, Heidelberg University, Heidelberg, Germany; 243 Federal Ministry of Education and Research, Berlin, Germany; 244 Melanoma Institute Australia, University of Sydney, Sydney, NSW Australia; 245 Pediatric Hematology and Oncology, University Hospital Muenster, Muenster, Germany; 246 Department of Pathology, Johns Hopkins University School of Medicine, Baltimore, MD USA; 247 McKusick-Nathans Institute of Genetic Medicine, Sidney Kimmel Comprehensive Cancer Center at Johns Hopkins University School of Medicine, Baltimore, MD USA; 248 Foundation Medicine, Inc, Cambridge, MA USA; 249 Department of Biomedical Data Science, Stanford University School of Medicine, Stanford, CA USA; 250 Department of Genetics, Stanford University School of Medicine, Stanford, CA USA; 251 Bakar Computational Health Sciences Institute and Department of Pediatrics, University of California, San Francisco, CA USA; 252 Institute of Clinical Medicine, Faculty of Medicine, University of Oslo, Oslo, Norway; 253 National Cancer Institute, National Institutes of Health, Bethesda, MD USA; 254 Royal Marsden NHS Foundation Trust, London and Sutton, UK; 255 Genome Biology Unit, European Molecular Biology Laboratory (EMBL), Heidelberg, Germany; 256 Department of Oncology, University of Cambridge, Cambridge, UK; 257 Li Ka Shing Centre, Cancer Research UK Cambridge Institute, University of Cambridge, Cambridge, UK; 258 Institut Gustave Roussy, Villejuif, France; 259 Cambridge University Hospitals NHS Foundation Trust, Cambridge, UK; 260 Department of Haematology, University of Cambridge, Cambridge, UK; 261 Anatomia Patológica, Hospital Clinic, Institut d’Investigacions Biomèdiques August Pi i Sunyer (IDIBAPS), University of Barcelona, Barcelona, Spain; 262 Spanish Ministry of Science and Innovation, Madrid, Spain; 263 University of Michigan Comprehensive Cancer Center, Ann Arbor, MI USA; 264 Department for BioMedical Research, University of Bern, Bern, Switzerland; 265 Department of Medical Oncology, Inselspital, University Hospital and University of Bern, Bern, Switzerland; 266 Graduate School for Cellular and Biomedical Sciences, University of Bern, Bern, Switzerland; 267 University of Pavia, Pavia, Italy; 268 University of Alabama at Birmingham, Birmingham, AL USA; 269 UHN Program in BioSpecimen Sciences, Toronto General Hospital, Toronto, ON Canada; 270 Department of Urology, Icahn School of Medicine at Mount Sinai, New York, NY USA; 271 Centre for Law and Genetics, University of Tasmania, Sandy Bay Campus, Hobart, TAS Australia; 272 Faculty of Biosciences, Heidelberg University, Heidelberg, Germany; 273 Department of Biochemistry, Microbiology and Immunology, Faculty of Medicine, University of Ottawa, Ottawa, ON Canada; 274 Division of Anatomic Pathology, Mayo Clinic, Rochester, MN USA; 275 Division of Cancer Epidemiology and Genetics, National Cancer Institute, National Institutes of Health, Bethesda, MD USA; 276 Illawarra Shoalhaven Local Health District L3 Illawarra Cancer Care Centre, Wollongong Hospital, Wollongong, NSW Australia; 277 BioForA, French National Institute for Agriculture, Food, and Environment (INRAE), ONF, Orléans, France; 278 Department of Biostatistics, Bloomberg School of Public Health, Johns Hopkins University, Baltimore, MD USA; 279 University of California San Diego, San Diego, CA USA; 280 Division of Experimental Pathology, Mayo Clinic, Rochester, MN USA; 281 Centre for Cancer Research, The Westmead Institute for Medical Research, University of Sydney, Sydney, NSW Australia; 282 Department of Gynaecological Oncology, Westmead Hospital, Sydney, NSW Australia; 283 PDXen Biosystems Inc, Seoul, South Korea; 284 Korea Advanced Institute of Science and Technology, Daejeon, South Korea; 285 Electronics and Telecommunications Research Institute, Daejeon, South Korea; 286 Institut National du Cancer (INCA), Boulogne-Billancourt, France; 287 Department of Genetics, Informatics Institute, University of Alabama at Birmingham, Birmingham, AL USA; 288 Division of Medical Oncology, National Cancer Centre, Singapore, Singapore; 289 Medical Oncology, University and Hospital Trust of Verona, Verona, Italy; 290 Department of Pediatrics, University Hospital Schleswig-Holstein, Kiel, Germany; 291 Hepatobiliary/Pancreatic Surgical Oncology Program, University Health Network, Toronto, ON Canada; 292 School of Biological Sciences, University of Auckland, Auckland, New Zealand; 293 Department of Surgery, University of Melbourne, Parkville, VIC Australia; 294 The Murdoch Children’s Research Institute, Royal Children’s Hospital, Parkville, VIC Australia; 295 Walter and Eliza Hall Institute, Parkville, VIC Australia; 296 Vancouver Prostate Centre, Vancouver, Canada; 297 Lunenfeld-Tanenbaum Research Institute, Mount Sinai Hospital, Toronto, ON Canada; 298 University of East Anglia, Norwich, UK; 299 Norfolk and Norwich University Hospital NHS Trust, Norwich, UK; 300 Victorian Institute of Forensic Medicine, Southbank, VIC Australia; 301 Department of Biomedical Informatics, Harvard Medical School, Boston, MA USA; 302 Department of Chemistry, Centre for Molecular Science Informatics, University of Cambridge, Cambridge, UK; 303 Ludwig Center at Harvard Medical School, Boston, MA USA; 304 Human Genome Sequencing Center, Baylor College of Medicine, Houston, TX USA; 305 Peter MacCallum Cancer Centre, University of Melbourne, Melbourne, VIC Australia; 306 Physics Division, Optimization and Systems Biology Lab, Massachusetts General Hospital, Boston, MA USA; 307 Department of Medicine, Baylor College of Medicine, Houston, TX USA; 308 University of Cologne, Cologne, Germany; 309 International Genomics Consortium, Phoenix, AZ USA; 310 Genomics Research Program, Ontario Institute for Cancer Research, Toronto, ON Canada; 311 Barking Havering and Redbridge University Hospitals NHS Trust, Romford, UK; 312 Children’s Hospital at Westmead, University of Sydney, Sydney, NSW Australia; 313 Department of Medicine, Section of Endocrinology, University and Hospital Trust of Verona, Verona, Italy; 314 Computational Biology Center, Memorial Sloan Kettering Cancer Center, New York, NY USA; 315 Department of Biology, ETH Zurich, Zürich, Switzerland; 316 Department of Computer Science, ETH Zurich, Zurich, Switzerland; 317 SIB Swiss Institute of Bioinformatics, Lausanne, Switzerland; 318 Weill Cornell Medical College, New York, NY USA; 319 Academic Department of Medical Genetics, University of Cambridge, Addenbrooke’s Hospital, Cambridge, UK; 320 MRC Cancer Unit, University of Cambridge, Cambridge, UK; 321 Departments of Pediatrics and Genetics, University of North Carolina at Chapel Hill, Chapel Hill, NC USA; 322 Seven Bridges Genomics, Charlestown, MA USA; 323 Annai Systems, Inc, Carlsbad, CA USA; 324 Department of Pathology, General Hospital of Treviso, Department of Medicine, University of Padua, Treviso, Italy; 325 Department of Computational Biology, University of Lausanne, Lausanne, Switzerland; 326 Department of Genetic Medicine and Development, University of Geneva Medical School, Geneva, CH Switzerland; 327 Swiss Institute of Bioinformatics, University of Geneva, Geneva, CH Switzerland; 328 The Francis Crick Institute, London, UK; 329 University of Leuven, Leuven, Belgium; 330 Institute of Medical Genetics and Applied Genomics, University of Tübingen, Tübingen, Germany; 331 Computational and Systems Biology, Genome Institute of Singapore, Singapore, Singapore; 332 School of Computing, National University of Singapore, Singapore, Singapore; 333 Big Data Institute, Li Ka Shing Centre, University of Oxford, Oxford, UK; 334 Biomedical Data Science Laboratory, Francis Crick Institute, London, UK; 335 Bioinformatics Group, Department of Computer Science, University College London, London, UK; 336 The Edward S. Rogers Sr. Department of Electrical and Computer Engineering, University of Toronto, Toronto, ON Canada; 337 Breast Cancer Translational Research Laboratory JC Heuson, Institut Jules Bordet, Brussels, Belgium; 338 Department of Oncology, Laboratory for Translational Breast Cancer Research, KU Leuven, Leuven, Belgium; 339 Institute for Research in Biomedicine (IRB Barcelona), The Barcelona Institute of Science and Technology, Barcelona, Spain; 340 Research Program on Biomedical Informatics, Universitat Pompeu Fabra, Barcelona, Spain; 341 Division of Medical Oncology, Princess Margaret Cancer Centre, Toronto, ON Canada; 342 Department of Physiology and Biophysics, Weill Cornell Medicine, New York, NY USA; 343 Institute for Computational Biomedicine, Weill Cornell Medicine, New York, NY USA; 344 Department of Pathology, UPMC Shadyside, Pittsburgh, PA USA; 345 Independent Consultant, Wellesley, USA; 346 Department of Cell and Molecular Biology, Science for Life Laboratory, Uppsala University, Uppsala, Sweden; 347 Department of Medicine and Department of Genetics, Washington University School of Medicine, St. Louis, St. Louis, MO USA; 348 Hefei University of Technology, Anhui, China; 349 Translational Cancer Research Unit, GZA Hospitals St.-Augustinus, Center for Oncological Research, Faculty of Medicine and Health Sciences, University of Antwerp, Antwerp, Belgium; 350 Simon Fraser University, Burnaby, BC Canada; 351 University of Pennsylvania, Philadelphia, PA USA; 352 Faculty of Science and Technology, University of Vic—Central University of Catalonia (UVic-UCC), Vic, Spain; 353 The Wellcome Trust, London, UK; 354 The Hospital for Sick Children, Toronto, ON Canada; 355 Department of Pathology, Queen Elizabeth University Hospital, Glasgow, UK; 356 Department of Genetics and Computational Biology, QIMR Berghofer Medical Research Institute, Brisbane, QLD Australia; 357 Department of Oncology, Centre for Cancer Genetic Epidemiology, University of Cambridge, Cambridge, UK; 358 Department of Public Health and Primary Care, Centre for Cancer Genetic Epidemiology, University of Cambridge, Cambridge, UK; 359 Prostate Cancer Canada, Toronto, ON Canada; 360 University of Cambridge, Cambridge, UK; 361 Department of Laboratory Medicine, Translational Cancer Research, Lund University Cancer Center at Medicon Village, Lund University, Lund, Sweden; 362 Heidelberg University, Heidelberg, Germany; 363 New BIH Digital Health Center, Berlin Institute of Health (BIH) and Charité - Universitätsmedizin Berlin, Berlin, Germany; 364 CIBER Epidemiología y Salud Pública (CIBERESP), Madrid, Spain; 365 Research Group on Statistics, Econometrics and Health (GRECS), UdG, Barcelona, Spain; 366 Quantitative Genomics Laboratories (qGenomics), Barcelona, Spain; 367 Icelandic Cancer Registry, Icelandic Cancer Society, Reykjavik, Iceland; 368 State Key Laboratory of Cancer Biology, and Xijing Hospital of Digestive Diseases, Fourth Military Medical University, Shaanxi, China; 369 Department of Medicine (DIMED), Surgical Pathology Unit, University of Padua, Padua, Italy; 370 Rigshospitalet, Copenhagen, Denmark; 371 Center for Cancer Genomics, National Cancer Institute, National Institutes of Health, Bethesda, MD USA; 372 Department of Biochemistry and Molecular Medicine, University of Montreal, Montreal, QC Canada; 373 Australian Institute of Tropical Health and Medicine, James Cook University, Douglas, QLD Australia; 374 Department of Neuro-Oncology, Istituto Neurologico Besta, Milano, Italy; 375 Bioplatforms Australia, North Ryde, NSW Australia; 376 Department of Pathology (Research), University College London Cancer Institute, London, UK; 377 Department of Surgical Oncology, Princess Margaret Cancer Centre, Toronto, ON Canada; 378 Department of Medical Oncology, Josephine Nefkens Institute and Cancer Genomics Centre, Erasmus Medical Center, Rotterdam, CN The Netherlands; 379 The University of Queensland Thoracic Research Centre, The Prince Charles Hospital, Brisbane, QLD Australia; 380 CIBIO/InBIO - Research Center in Biodiversity and Genetic Resources, Universidade do Porto, Vairão, Portugal; 381 HCA Laboratories, London, UK; 382 University of Liverpool, Liverpool, UK; 383 The Azrieli Faculty of Medicine, Bar-Ilan University, Safed, Israel; 384 Department of Neurosurgery, University of Florida, Gainesville, FL USA; 385 Department of Pathology, Graduate School of Medicine, University of Tokyo, Tokyo, Japan; 386 University of Milano Bicocca, Monza, Italy; 387 BGI-Shenzhen, Shenzhen, China; 388 Department of Pathology, Oslo University Hospital Ulleval, Oslo, Norway; 389 Center for Biomedical Informatics, Harvard Medical School, Boston, MA USA; 390 Department Biochemistry and Molecular Biomedicine, University of Barcelona, Barcelona, Spain; 391 Office of Cancer Genomics, National Cancer Institute, National Institutes of Health, Bethesda, MD USA; 392 Cancer Epigenomics, German Cancer Research Center (DKFZ), Heidelberg, Germany; 393 Department of Cancer Biology, The University of Texas MD Anderson Cancer Center, Houston, TX USA; 394 Department of Surgical Oncology, The University of Texas MD Anderson Cancer Center, Houston, TX USA; 395 Department of Computer Science, Yale University, New Haven, CT USA; 396 Department of Molecular Biophysics and Biochemistry, Yale University, New Haven, CT USA; 397 Program in Computational Biology and Bioinformatics, Yale University, New Haven, CT USA; 398 Center for Cancer Research, Massachusetts General Hospital, Boston, MA USA; 399 Department of Pathology, Massachusetts General Hospital, Boston, MA USA; 400 Department of Pathology, Memorial Sloan Kettering Cancer Center, New York, NY USA; 401 Division of Gastroenterology and Hepatology, Mayo Clinic, Rochester, MN USA; 402 University of Sydney, Sydney, NSW Australia; 403 University of Oxford, Oxford, UK; 404 Department of Surgery, Academic Urology Group, University of Cambridge, Cambridge, UK; 405 Department of Medicine II, University of Würzburg, Wuerzburg, Germany; 406 Sylvester Comprehensive Cancer Center, University of Miami, Miami, FL USA; 407 Institut Hospital del Mar d’Investigacions Mèdiques (IMIM), Barcelona, Spain; 408 Genome Integrity and Structural Biology Laboratory, National Institute of Environmental Health Sciences (NIEHS), Durham, NC USA; 409 St. Thomas’s Hospital, London, UK; 410 Osaka International Cancer Center, Osaka, Japan; 411 Department of Pathology, Skåne University Hospital, Lund University, Lund, Sweden; 412 Department of Medical Oncology, Beatson West of Scotland Cancer Centre, Glasgow, UK; 413 National Human Genome Research Institute, National Institutes of Health, Bethesda, MD USA; 414 Centre for Cancer Research, Victorian Comprehensive Cancer Centre, University of Melbourne, Melbourne, VIC Australia; 415 Department of Medicine, Section of Hematology/Oncology, University of Chicago, Chicago, IL USA; 416 German Center for Infection Research (DZIF), Partner Site Hamburg-Borstel-Lübeck-Riems, Hamburg, Germany; 417 Bioinformatics Research Centre (BiRC), Aarhus University, Aarhus, Denmark; 418 Department of Biotechnology, Ministry of Science and Technology, Government of India, New Delhi, Delhi India; 419 National Cancer Centre Singapore, Singapore, Singapore; 420 Brandeis University, Waltham, MA USA; 421 Department of Urologic Sciences, University of British Columbia, Vancouver, BC Canada; 422 Department of Internal Medicine, Stanford University, Stanford, CA USA; 423 The University of Texas Health Science Center at Houston, Houston, TX USA; 424 Imperial College NHS Trust, Imperial College, London, INY UK; 425 Senckenberg Institute of Pathology, University of Frankfurt Medical School, Frankfurt, Germany; 426 Department of Medicine, Division of Biomedical Informatics, UC San Diego School of Medicine, San Diego, CA USA; 427 Center for Precision Health, School of Biomedical Informatics, The University of Texas Health Science Center, Houston, TX USA; 428 Oxford Nanopore Technologies, New York, NY USA; 429 Institute of Medical Science, University of Tokyo, Tokyo, Japan; 430 Howard Hughes Medical Institute, University of California Santa Cruz, Santa Cruz, CA USA; 431 Wakayama Medical University, Wakayama, Japan; 432 Department of Internal Medicine, Division of Medical Oncology, Lineberger Comprehensive Cancer Center, University of North Carolina at Chapel Hill, Chapel Hill, NC USA; 433 University of Tennessee Health Science Center for Cancer Research, Memphis, TN USA; 434 Department of Histopathology, Salford Royal NHS Foundation Trust, Salford, UK; 435 Faculty of Biology, Medicine and Health, University of Manchester, Manchester, UK; 436 BIOPIC, ICG and College of Life Sciences, Peking University, Beijing, China; 437 Peking-Tsinghua Center for Life Sciences, Peking University, Beijing, China; 438 Children’s Hospital of Philadelphia, Philadelphia, PA USA; 439 Department of Bioinformatics and Computational Biology and Department of Systems Biology, The University of Texas MD Anderson Cancer Center, Houston, TX USA; 440 Karolinska Institute, Stockholm, Sweden; 441 The Donnelly Centre, University of Toronto, Toronto, ON Canada; 442 Department of Medical Genetics, College of Medicine, Hallym University, Chuncheon, South Korea; 443 Department of Experimental and Health Sciences, Institute of Evolutionary Biology (UPF-CSIC), Universitat Pompeu Fabra, Barcelona, Spain; 444 Health Data Science Unit, University Clinics, Heidelberg, Germany; 445 Massachusetts General Hospital Center for Cancer Research, Charlestown, MA USA; 446 Hokkaido University, Sapporo, Japan; 447 Department of Pathology and Clinical Laboratory, National Cancer Center Hospital, Tokyo, Japan; 448 Department of Genetics, University of North Carolina at Chapel Hill, Chapel Hill, NC USA; 449 Computational Biology, Leibniz Institute on Aging - Fritz Lipmann Institute (FLI), Jena, Germany; 450 University of Melbourne Centre for Cancer Research, Melbourne, VIC Australia; 451 University of Nebraska Medical Center, Omaha, NE USA; 452 Syntekabio Inc, Daejeon, South Korea; 453 Department of Pathology, Academic Medical Center, Amsterdam, AZ The Netherlands; 454 China National GeneBank-Shenzhen, Shenzhen, China; 455 Division of Molecular Genetics, German Cancer Research Center (DKFZ), Heidelberg, Germany; 456 Division of Life Science and Applied Genomics Center, Hong Kong University of Science and Technology, Clear Water Bay, Hong Kong, China; 457 Icahn School of Medicine at Mount Sinai, New York, NY USA; 458 Geneplus-Shenzhen, Shenzhen, China; 459 School of Computer Science and Technology, Xi’an Jiaotong University, Xi’an, China; 460 AbbVie, North Chicago, IL USA; 461 Institute of Pathology, Charité – University Medicine Berlin, Berlin, Germany; 462 Centre for Translational and Applied Genomics, British Columbia Cancer Agency, Vancouver, BC Canada; 463 Edinburgh Royal Infirmary, Edinburgh, UK; 464 Berlin Institute for Medical Systems Biology, Max Delbrück Center for Molecular Medicine, Berlin, Germany; 465 Department of Pediatric Immunology, Hematology and Oncology, University Hospital, Heidelberg, Germany; 466 German Cancer Research Center (DKFZ), Heidelberg, Germany; 467 Heidelberg Institute for Stem Cell Technology and Experimental Medicine (HI-STEM), Heidelberg, Germany; 468 Institute for Computational Biomedicine, Weill Cornell Medical College, New York, NY USA; 469 New York Genome Center, New York, NY USA; 470 Department of Urology, James Buchanan Brady Urological Institute, Johns Hopkins University School of Medicine, Baltimore, MD USA; 471 Department of Preventive Medicine, Graduate School of Medicine, The University of Tokyo, Tokyo, Japan; 472 Department of Molecular and Cellular Biology, Baylor College of Medicine, Houston, TX USA; 473 Department of Pathology and Immunology, Baylor College of Medicine, Houston, TX USA; 474 Michael E. DeBakey Veterans Affairs Medical Center, Houston, TX USA; 475 Technical University of Denmark, Lyngby, Denmark; 476 Department of Pathology, College of Medicine, Hanyang University, Seoul, South Korea; 477 Academic Unit of Surgery, School of Medicine, College of Medical, Veterinary and Life Sciences, University of Glasgow, Glasgow Royal Infirmary, Glasgow, UK; 478 Department of Pathology, Asan Medical Center, College of Medicine, Ulsan University, Songpa-gu, Seoul South Korea; 479 Science Writer, Garrett Park, MD USA; 480 International Cancer Genome Consortium (ICGC)/ICGC Accelerating Research in Genomic Oncology (ARGO) Secretariat, Ontario Institute for Cancer Research, Toronto, ON Canada; 481 University of Ljubljana, Ljubljana, Slovenia; 482 Department of Public Health Sciences, University of Chicago, Chicago, IL USA; 483 Research Institute, NorthShore University HealthSystem, Evanston, IL USA; 484 Department for Biomedical Research, University of Bern, Bern, Switzerland; 485 Centre of Genomics and Policy, McGill University and Génome Québec Innovation Centre, Montreal, QC Canada; 486 Carolina Center for Genome Sciences, University of North Carolina at Chapel Hill, Chapel Hill, NC USA; 487 Hopp Children’s Cancer Center (KiTZ), Heidelberg, Germany; 488 Pediatric Glioma Research Group, German Cancer Research Center (DKFZ), Heidelberg, Germany; 489 Cancer Research UK, London, UK; 490 Indivumed GmbH, Hamburg, Germany; 491 Genome Integration Data Center, Syntekabio, Inc, Daejeon, South Korea; 492 University Hospital Zurich, Zurich, Switzerland; 493 Clinical Bioinformatics, Swiss Institute of Bioinformatics, Geneva, Switzerland; 494 Institute for Pathology and Molecular Pathology, University Hospital Zurich, Zurich, Switzerland; 495 Institute of Molecular Life Sciences, University of Zurich, Zurich, Switzerland; 496 MRC Human Genetics Unit, MRC IGMM, University of Edinburgh, Edinburgh, UK; 497 Women’s Cancer Program at the Samuel Oschin Comprehensive Cancer Institute, Cedars-Sinai Medical Center, Los Angeles, CA USA; 498 Department of Biology, Bioinformatics Group, Division of Molecular Biology, Faculty of Science, University of Zagreb, Zagreb, Croatia; 499 Department for Internal Medicine II, University Hospital Schleswig-Holstein, Kiel, Germany; 500 Genetics and Molecular Pathology, SA Pathology, Adelaide, SA Australia; 501 Department of Gastric Surgery, National Cancer Center Hospital, Tokyo, Japan; 502 Department of Bioinformatics, Division of Cancer Genomics, National Cancer Center Research Institute, Tokyo, Japan; 503 A.A. Kharkevich Institute of Information Transmission Problems, Moscow, Russia; 504 Oncology and Immunology, Dmitry Rogachev National Research Center of Pediatric Hematology, Moscow, Russia; 505 Skolkovo Institute of Science and Technology, Moscow, Russia; 506 Department of Surgery, The George Washington University, School of Medicine and Health Science, Washington, DC USA; 507 Endocrine Oncology Branch, Center for Cancer Research, National Cancer Institute, National Institutes of Health, Bethesda, MD USA; 508 Melanoma Institute Australia, Macquarie University, Sydney, NSW Australia; 509 MIT Computer Science and Artificial Intelligence Laboratory, Massachusetts Institute of Technology, Cambridge, MA USA; 510 Tissue Pathology and Diagnostic Oncology, Royal Prince Alfred Hospital, Sydney, NSW Australia; 511 Cholangiocarcinoma Screening and Care Program and Liver Fluke and Cholangiocarcinoma Research Centre, Faculty of Medicine, Khon Kaen University, Khon Kaen, Thailand; 512 Controlled Department and Institution, New York, NY USA; 513 Englander Institute for Precision Medicine, Weill Cornell Medicine, New York, NY USA; 514 National Cancer Center, Gyeonggi, South Korea; 515 Department of Biochemistry, College of Medicine, Ewha Womans University, Seoul, South Korea; 516 Health Sciences Department of Biomedical Informatics, University of California San Diego, La Jolla, CA USA; 517 Research Core Center, National Cancer Centre Korea, Goyang-si, South Korea; 518 Department of Health Sciences and Technology, Sungkyunkwan University School of Medicine, Seoul, South Korea; 519 Samsung Genome Institute, Seoul, South Korea; 520 Breast Oncology Program, Dana-Farber/Brigham and Women’s Cancer Center, Boston, MA USA; 521 Department of Surgery, Memorial Sloan Kettering Cancer Center, New York, NY USA; 522 Division of Breast Surgery, Brigham and Women’s Hospital, Boston, MA USA; 523 Integrative Bioinformatics Support Group, National Institute of Environmental Health Sciences (NIEHS), Durham, NC USA; 524 Department of Clinical Science, University of Bergen, Bergen, Norway; 525 Center For Medical Innovation, Seoul National University Hospital, Seoul, South Korea; 526 Department of Internal Medicine, Seoul National University Hospital, Seoul, South Korea; 527 Institute of Computer Science, Polish Academy of Sciences, Warsawa, Poland; 528 Functional and Structural Genomics, German Cancer Research Center (DKFZ), Heidelberg, Germany; 529 Laboratory of Translational Genomics, Division of Cancer Epidemiology and Genetics, National Cancer Institute, , National Institutes of Health, Bethesda, MD USA; 530 Institute for Medical Informatics Statistics and Epidemiology, University of Leipzig, Leipzig, Germany; 531 Morgan Welch Inflammatory Breast Cancer Research Program and Clinic, The University of Texas MD Anderson Cancer Center, Houston, TX USA; 532 Department of Hematology and Oncology, Georg-Augusts-University of Göttingen, Göttingen, Germany; 533 Institute of Cell Biology (Cancer Research), University of Duisburg-Essen, Essen, Germany; 534 King’s College London and Guy’s and St. Thomas’ NHS Foundation Trust, London, UK; 535 Center for Epigenetics, Van Andel Research Institute, Grand Rapids, MI USA; 536 The University of Queensland Centre for Clinical Research, Royal Brisbane and Women’s Hospital, Herston, QLD Australia; 537 Department of Pediatric Oncology and Hematology, University of Cologne, Cologne, Germany; 538 University of Düsseldorf, Düsseldorf, Germany; 539 Department of Pathology, Institut Jules Bordet, Brussels, Belgium; 540 Institute of Biomedicine, Sahlgrenska Academy at University of Gothenburg, Gothenburg, Sweden; 541 Children’s Medical Research Institute, Sydney, NSW Australia; 542 ILSbio, LLC Biobank, Chestertown, MD USA; 543 Division of Genetics and Genomics, Boston Children’s Hospital, Harvard Medical School, Boston, MA USA; 544 Institute for Bioengineering and Biopharmaceutical Research (IBBR), Hanyang University, Seoul, South Korea; 545 Department of Statistics, University of California Santa Cruz, Santa Cruz, CA USA; 546 National Genotyping Center, Institute of Biomedical Sciences, Academia Sinica, Taipei, Taiwan; 547 Department of Vertebrate Genomics/Otto Warburg Laboratory Gene Regulation and Systems Biology of Cancer, Max Planck Institute for Molecular Genetics, Berlin, Germany; 548 McGill University and Genome Quebec Innovation Centre, Montreal, QC Canada; 549 biobyte solutions GmbH, Heidelberg, Germany; 550 Gynecologic Oncology, NYU Laura and Isaac Perlmutter Cancer Center, New York University, New York, NY USA; 551 Division of Oncology, Stem Cell Biology Section, Washington University School of Medicine, St. Louis, MO USA; 552 Department of Systems Biology, The University of Texas MD Anderson Cancer Center, Houston, TX USA; 553 Harvard University, Cambridge, MA USA; 554 Urologic Oncology Branch, Center for Cancer Research, National Cancer Institute, National Institutes of Health, Bethesda, MD USA; 555 University of Oslo, Oslo, Norway; 556 University of Toronto, Toronto, ON Canada; 557 Peking University, Beijing, China; 558 School of Life Sciences, Peking University, Beijing, China; 559 Leidos Biomedical Research, Inc, McLean, VA USA; 560 Hematology, Hospital Clinic, Institut d’Investigacions Biomèdiques August Pi i Sunyer (IDIBAPS), University of Barcelona, Barcelona, Spain; 561 Second Military Medical University, Shanghai, China; 562 Chinese Cancer Genome Consortium, Shenzhen, China; 563 Department of Medical Oncology, Beijing Hospital, Beijing, China; 564 Laboratory of Molecular Oncology, Key Laboratory of Carcinogenesis and Translational Research (Ministry of Education), Peking University Cancer Hospital and Institute, Beijing, China; 565 School of Medicine/School of Mathematics and Statistics, University of St. Andrews, St, Andrews, Fife UK; 566 Institute for Systems Biology, Seattle, WA USA; 567 Department of Biochemistry and Molecular Biology, Faculty of Medicine, University Institute of Oncology-IUOPA, Oviedo, Spain; 568 Institut Bergonié, Bordeaux, France; 569 Cancer Unit, MRC University of Cambridge, Cambridge, UK; 570 Department of Pathology and Laboratory Medicine, Center for Personalized Medicine, Children’s Hospital Los Angeles, Los Angeles, CA USA; 571 John Curtin School of Medical Research, Canberra, ACT Australia; 572 MVZ Department of Oncology, PraxisClinic am Johannisplatz, Leipzig, Germany; 573 Department of Information Technology, Ghent University, Ghent, Belgium; 574 Department of Plant Biotechnology and Bioinformatics, Ghent University, Ghent, Belgium; 575 Institute for Genomic Medicine, Nationwide Children’s Hospital, Columbus, OH USA; 576 Computational Biology Program, School of Medicine, Oregon Health and Science University, Portland, OR USA; 577 Department of Surgery, Duke University, Durham, NC USA; 578 Institució Catalana de Recerca i Estudis Avançats (ICREA), Barcelona, Spain; 579 Institut Català de Paleontologia Miquel Crusafont, Universitat Autònoma de Barcelona, Barcelona, Spain; 580 University of Glasgow, Glasgow, UK; 581 Institut d’Investigacions Biomèdiques August Pi i Sunyer (IDIBAPS), Barcelona, Spain; 582 Division of Oncology, Washington University School of Medicine, St. Louis, MO USA; 583 Department of Surgery and Cancer, Imperial College, London, INY UK; 584 Applications Department, Oxford Nanopore Technologies, Oxford, UK; 585 Department of Obstetrics, Gynecology and Reproductive Services, University of California San Francisco, San Francisco, CA USA; 586 Department of Biochemistry and Molecular Medicine, University California at Davis, Sacramento, CA USA; 587 STTARR Innovation Facility, Princess Margaret Cancer Centre, Toronto, ON Canada; 588 Discipline of Surgery, Western Sydney University, Penrith, NSW Australia; 589 Yale School of Medicine, Yale University, New Haven, CT USA; 590 Department of Genetics, Lineberger Comprehensive Cancer Center, University of North Carolina at Chapel Hill, Chapel Hill, NC USA; 591 Departments of Neurology and Neurosurgery, Henry Ford Hospital, Detroit, MI USA; 592 Precision Oncology, OHSU Knight Cancer Institute, Oregon Health and Science University, Portland, OR USA; 593 Institute of Pathology, University Medical Center Hamburg-Eppendorf, Hamburg, Germany; 594 Department of Health Sciences, Faculty of Medical Sciences, Kyushu University, Fukuoka, Japan; 595 Heidelberg Academy of Sciences and Humanities, Heidelberg, Germany; 596 Department of Clinical Pathology, University of Melbourne, Melbourne, VIC, Australia; 597 Department of Pathology, Roswell Park Cancer Institute, Buffalo, NY USA; 598 Department of Computer Science, University of Helsinki, Helsinki, Finland; 599 Institute of Biotechnology, University of Helsinki, Helsinki, Finland; 600 Organismal and Evolutionary Biology Research Programme, University of Helsinki, Helsinki, Finland; 601 Department of Obstetrics and Gynecology, Division of Gynecologic Oncology, Washington University School of Medicine, St. Louis, MO USA; 602 Penrose St. Francis Health Services, Colorado Springs, CO USA; 603 Institute of Pathology, Ulm University and University Hospital of Ulm, Ulm, Germany; 604 National Cancer Center, Tokyo, Japan; 605 Genome Institute of Singapore, Singapore, Singapore; 606 32Program in Computational Biology and Bioinformatics, Yale University, New Haven, CT USA; 607 German Cancer Aid, Bonn, Germany; 608 Programme in Cancer and Stem Cell Biology, Centre for Computational Biology, Duke-NUS Medical School, Singapore, Singapore; 609 The Chinese University of Hong Kong, Shatin, NT, Hong Kong China; 610 Fourth Military Medical University, Shaanxi, China; 611 The University of Cambridge School of Clinical Medicine, Cambridge, UK; 612 St. Jude Children’s Research Hospital, Memphis, TN USA; 613 University Health Network, Princess Margaret Cancer Centre, Toronto, ON Canada; 614 Center for Biomolecular Science and Engineering, University of California Santa Cruz, Santa Cruz, CA USA; 615 Department of Medicine, University of Chicago, Chicago, IL USA; 616 Department of Neurology, Mayo Clinic, Rochester, MN USA; 617 Cambridge Oesophagogastric Centre, Cambridge University Hospitals NHS Foundation Trust, Cambridge, UK; 618 Department of Computer Science, Carleton College, Northfield, MN USA; 619 Institute of Cancer Sciences, College of Medical Veterinary and Life Sciences, University of Glasgow, Glasgow, UK; 620 Department of Epidemiology, University of Alabama at Birmingham, Birmingham, AL USA; 621 HudsonAlpha Institute for Biotechnology, Huntsville, AL USA; 622 O’Neal Comprehensive Cancer Center, University of Alabama at Birmingham, Birmingham, AL USA; 623 Department of Pathology, Keio University School of Medicine, Tokyo, Japan; 624 Department of Hepatobiliary and Pancreatic Oncology, National Cancer Center Hospital, Tokyo, Japan; 625 Sage Bionetworks, Seattle, WA USA; 626 Lymphoma Genomic Translational Research Laboratory, National Cancer Centre, Singapore, Singapore; 627 Department of Clinical Pathology, Robert-Bosch-Hospital, Stuttgart, Germany; 628 Department of Cell and Systems Biology, University of Toronto, Toronto, ON Canada; 629 Department of Biosciences and Nutrition, Karolinska Institutet, Stockholm, Sweden; 630 Center for Liver Cancer, Research Institute and Hospital, National Cancer Center, Gyeonggi, South Korea; 631 Division of Hematology-Oncology, Samsung Medical Center, Sungkyunkwan University School of Medicine, Seoul, South Korea; 632 Samsung Advanced Institute for Health Sciences and Technology, Sungkyunkwan University School of Medicine, Seoul, South Korea; 633 Cheonan Industry-Academic Collaboration Foundation, Sangmyung University, Cheonan, South Korea; 634 NYU Langone Medical Center, New York, NY USA; 635 Department of Hematology and Medical Oncology, Cleveland Clinic, Cleveland, OH USA; 636 Department of Radiation Oncology, University of California San Francisco, San Francisco, CA USA; 637 Department of Health Sciences Research, Mayo Clinic, Rochester, MN USA; 638 Helen F. Graham Cancer Center at Christiana Care Health Systems, Newark, DE USA; 639 Heidelberg University Hospital, Heidelberg, Germany; 640 CSRA Incorporated, Fairfax, VA USA; 641 Research Department of Pathology, University College London Cancer Institute, London, UK; 642 Department of Research Oncology, Guy’s Hospital, King’s Health Partners AHSC, King’s College London School of Medicine, London, UK; 643 Faculty of Medicine and Health Sciences, Macquarie University, Sydney, NSW Australia; 644 University Hospital of Minjoz, INSERM UMR 1098, Besançon, France; 645 Spanish National Cancer Research Centre, Madrid, Spain; 646 Center of Digestive Diseases and Liver Transplantation, Fundeni Clinical Institute, Bucharest, Romania; 647 Cureline, Inc, South San Francisco, CA USA; 648 St. Luke’s Cancer Centre, Royal Surrey County Hospital NHS Foundation Trust, Guildford, UK; 649 Cambridge Breast Unit, Addenbrooke’s Hospital, Cambridge University Hospital NHS Foundation Trust and NIHR Cambridge Biomedical Research Centre, Cambridge, UK; 650 East of Scotland Breast Service, Ninewells Hospital, Aberdeen, UK; 651 Department of Genetics, Microbiology and Statistics, University of Barcelona, IRSJD, IBUB, Barcelona, Spain; 652 Department of Obstetrics and Gynecology, Medical College of Wisconsin, Milwaukee, WI USA; 653 Hematology and Medical Oncology, Winship Cancer Institute of Emory University, Atlanta, GA USA; 654 Department of Computer Science, Princeton University, Princeton, NJ USA; 655 Vanderbilt Ingram Cancer Center, Vanderbilt University, Nashville, TN USA; 656 Ohio State University College of Medicine and Arthur G. James Comprehensive Cancer Center, Columbus, OH USA; 657 Department of Surgery, Yokohama City University Graduate School of Medicine, Kanagawa, Japan; 658 Division of Chromatin Networks, German Cancer Research Center (DKFZ) and BioQuant, Heidelberg, Germany; 659 Research Computing Center, University of North Carolina at Chapel Hill, Chapel Hill, NC USA; 660 School of Molecular Biosciences and Center for Reproductive Biology, Washington State University, Pullman, WA USA; 661 Finsen Laboratory and Biotech Research and Innovation Centre (BRIC), University of Copenhagen, Copenhagen, Denmark; 662 Department of Laboratory Medicine and Pathobiology, University of Toronto, Toronto, ON Canada; 663 Department of Pathology, Human Oncology and Pathogenesis Program, Memorial Sloan Kettering Cancer Center, New York, NY USA; 664 University Hospital Giessen, Pediatric Hematology and Oncology, Giessen, Germany; 665 Oncologie Sénologie, ICM Institut Régional du Cancer, Montpellier, France; 666 Institute of Clinical Molecular Biology, Christian-Albrechts-University, Kiel, Germany; 667 Institute of Pathology, University of Wuerzburg, Wuerzburg, Germany; 668 Department of Urology, North Bristol NHS Trust, Bristol, UK; 669 SingHealth, Duke-NUS Institute of Precision Medicine, National Heart Centre Singapore, Singapore, Singapore; 670 Department of Computer Science, University of Toronto, Toronto, ON Canada; 671 Bern Center for Precision Medicine, University Hospital of Bern, University of Bern, Bern, Switzerland; 672 Englander Institute for Precision Medicine, Weill Cornell Medicine and New York Presbyterian Hospital, New York, NY USA; 673 Meyer Cancer Center, Weill Cornell Medicine, New York, NY USA; 674 Pathology and Laboratory, Weill Cornell Medical College, New York, NY USA; 675 Vall d’Hebron Institute of Oncology: VHIO, Barcelona, Spain; 676 General and Hepatobiliary-Biliary Surgery, Pancreas Institute, University and Hospital Trust of Verona, Verona, Italy; 677 National Centre for Biological Sciences, Tata Institute of Fundamental Research, Bangalore, India; 678 Indiana University, Bloomington, IN USA; 679 Department of Pathology, GZA-ZNA Hospitals, Antwerp, Belgium; 680 Analytical Biological Services, Inc, Wilmington, DE USA; 681 Sydney Medical School, University of Sydney, Sydney, NSW Australia; 682 cBio Center, Dana-Farber Cancer Institute, Harvard Medical School, Boston, MA USA; 683 Department of Cell Biology, Harvard Medical School, Boston, MA USA; 684 Advanced Centre for Treatment Research and Education in Cancer, Tata Memorial Centre, Navi Mumbai, Maharashtra India; 685 School of Environmental and Life Sciences, Faculty of Science, The University of Newcastle, Ourimbah, NSW Australia; 686 Department of Dermatology, University Hospital of Essen, Essen, Germany; 687 Bioinformatics and Omics Data Analytics, German Cancer Research Center (DKFZ), Heidelberg, Germany; 688 Department of Urology, Charité Universitätsmedizin Berlin, Berlin, Germany; 689 Martini-Clinic, Prostate Cancer Center, University Medical Center Hamburg-Eppendorf, Hamburg, Germany; 690 Department of General Internal Medicine, University of Kiel, Kiel, Germany; 691 German Cancer Consortium (DKTK), Partner site Berlin, Berlin, Germany; 692 Cancer Research Institute, Beth Israel Deaconess Medical Center, Boston, MA USA; 693 University of Pittsburgh, Pittsburgh, PA USA; 694 Department of Ophthalmology and Ocular Genomics Institute, Massachusetts Eye and Ear, Harvard Medical School, Boston, MA USA; 695 Center for Psychiatric Genetics, NorthShore University HealthSystem, Evanston, IL USA; 696 Van Andel Research Institute, Grand Rapids, MI USA; 697 Laboratory of Molecular Medicine, Human Genome Center, Institute of Medical Science, University of Tokyo, Tokyo, Japan; 698 Japan Agency for Medical Research and Development, Tokyo, Japan; 699 Korea University, Seoul, South Korea; 700 Murtha Cancer Center, Walter Reed National Military Medical Center, Bethesda, MD USA; 701 Human Genetics, University of Kiel, Kiel, Germany; 702 Department of Oncologic Pathology, Dana-Farber Cancer Institute, Harvard Medical School, Boston, MA USA; 703 Oregon Health and Science University, Portland, OR USA; 704 Center for RNA Interference and Noncoding RNA, The University of Texas MD Anderson Cancer Center, Houston, TX USA; 705 Department of Experimental Therapeutics, The University of Texas MD Anderson Cancer Center, Houston, TX USA; 706 Department of Gynecologic Oncology and Reproductive Medicine, The University of Texas MD Anderson Cancer Center, Houston, TX USA; 707 University Hospitals Coventry and Warwickshire NHS Trust, Coventry, UK; 708 Department of Radiation Oncology, Radboud University Nijmegen Medical Centre, Nijmegen, GA The Netherlands; 709 Institute for Genomics and Systems Biology, University of Chicago, Chicago, IL USA; 710 Clinic for Hematology and Oncology, St.-Antonius-Hospital, Eschweiler, Germany; 711 Computational and Systems Biology Program, Memorial Sloan Kettering Cancer Center, New York, NY USA; 712 University of Iceland, Reykjavik, Iceland; 713 Division of Computational Genomics and Systems Genetics, German Cancer Research Center (DKFZ), Heidelberg, Germany; 714 Dundee Cancer Centre, Ninewells Hospital, Dundee, UK; 715 Department for Internal Medicine III, University of Ulm and University Hospital of Ulm, Ulm, Germany; 716 Institut Curie, INSERM Unit 830, Paris, France; 717 Department of Gastroenterology and Hepatology, Yokohama City University Graduate School of Medicine, Kanagawa, Japan; 718 Department of Laboratory Medicine, Radboud University Nijmegen Medical Centre, Nijmegen, GA The Netherlands; 719 Division of Cancer Genome Research, German Cancer Research Center (DKFZ), Heidelberg, Germany; 720 Department of General Surgery, Singapore General Hospital, Singapore, Singapore; 721 Cancer Science Institute of Singapore, National University of Singapore, Singapore, Singapore; 722 Department of Medical and Clinical Genetics, Genome-Scale Biology Research Program, University of Helsinki, Helsinki, Finland; 723 East Anglian Medical Genetics Service, Cambridge University Hospitals NHS Foundation Trust, Cambridge, UK; 724 Irving Institute for Cancer Dynamics, Columbia University, New York, NY USA; 725 Institute of Molecular and Cell Biology, Singapore, Singapore; 726 Laboratory of Cancer Epigenome, Division of Medical Science, National Cancer Centre Singapore, Singapore, Singapore; 727 Universite Lyon, INCa-Synergie, Centre Léon Bérard, Lyon, France; 728 Department of Urology, Mayo Clinic, Rochester, MN USA; 729 Royal National Orthopaedic Hospital - Stanmore, Stanmore, Middlesex UK; 730 Department of Biochemistry, Genetics and Immunology, University of Vigo, Vigo, Spain; 731 Giovanni Paolo II / I.R.C.C.S. Cancer Institute, Bari, BA Italy; 732 Neuroblastoma Genomics, German Cancer Research Center (DKFZ), Heidelberg, Germany; 733 Fondazione Policlinico Universitario Gemelli IRCCS, Rome, Italy, Rome, Italy; 734 University of Verona, Verona, Italy; 735 Centre National de Génotypage, CEA - Institute de Génomique, Evry, France; 736 CAPHRI Research School, Maastricht University, Maastricht, ER The Netherlands; 737 Department of Biopathology, Centre Léon Bérard, Lyon, France; 738 Université Claude Bernard Lyon 1, Villeurbanne, France; 739 Core Research for Evolutional Science and Technology (CREST), JST, Tokyo, Japan; 740 Department of Biological Sciences, Laboratory for Medical Science Mathematics, Graduate School of Science, University of Tokyo, Yokohama, Japan; 741 Department of Medical Science Mathematics, Medical Research Institute, Tokyo Medical and Dental University (TMDU), Tokyo, Japan; 742 Cancer Ageing and Somatic Mutation Programme, Wellcome Sanger Institute, Hinxton, UK; 743 University Hospitals Birmingham NHS Foundation Trust, Birmingham, UK; 744 Centre for Cancer Research and Cell Biology, Queen’s University, Belfast, UK; 745 Breast Medical Oncology, The University of Texas MD Anderson Cancer Center, Houston, TX USA; 746 Department of Surgery, Johns Hopkins University School of Medicine, Baltimore, MD USA; 747 Department of Oncology-Pathology, Science for Life Laboratory, Karolinska Institute, Stockholm, Sweden; 748 School of Cancer Sciences, Faculty of Medicine, University of Southampton, Southampton, UK; 749 Department of Gene Technology, Tallinn University of Technology, Tallinn, Estonia; 750 Genetics and Genome Biology Program, SickKids Research Institute, The Hospital for Sick Children, Toronto, ON Canada; 751 Departments of Neurosurgery and Hematology and Medical Oncology, Winship Cancer Institute and School of Medicine, Emory University, Atlanta, GA USA; 752 Department of Clinical and Molecular Medicine, Faculty of Medicine and Health Sciences, Norwegian University of Science and Technology, Trondheim, Norway; 753 Argmix Consulting, North Vancouver, BC Canada; 754 Department of Information Technology, Ghent University, Interuniversitair Micro-Electronica Centrum (IMEC), Ghent, Belgium; 755 Nuffield Department of Surgical Sciences, John Radcliffe Hospital, University of Oxford, Oxford, UK; 756 Institute of Mathematics and Computer Science, University of Latvia, Riga, LV Latvia; 757 Discipline of Pathology, Sydney Medical School, University of Sydney, Sydney, NSW Australia; 758 Department of Applied Mathematics and Theoretical Physics, Centre for Mathematical Sciences, University of Cambridge, Cambridge, UK; 759 Department of Epidemiology and Biostatistics, Memorial Sloan Kettering Cancer Center, New York, NY USA; 760 Department of Statistics, Columbia University, New York, NY USA; 761 Department of Immunology, Genetics and Pathology, Science for Life Laboratory, Uppsala University, Uppsala, Sweden; 762 School of Electronic and Information Engineering, Xi’an Jiaotong University, Xi’an, China; 763 Department of Histopathology, Cambridge University Hospitals NHS Foundation Trust, Cambridge, UK; 764 Oxford NIHR Biomedical Research Centre, University of Oxford, Oxford, UK; 765 Georgia Regents University Cancer Center, Augusta, GA USA; 766 Wythenshawe Hospital, Manchester, UK; 767 Department of Genetics, Washington University School of Medicine, St.Louis, MO USA; 768 Department of Biological Oceanography, Leibniz Institute of Baltic Sea Research, Rostock, Germany; 769 Wellcome Centre for Human Genetics, University of Oxford, Oxford, UK; 770 Department of Molecular and Human Genetics, Baylor College of Medicine, Houston, TX USA; 771 Thoracic Oncology Laboratory, Mayo Clinic, Rochester, MN USA; 772 Institute for Genomic Medicine, Nationwide Children’s Hospital, Columbus, OH USA; 773 Department of Obstetrics and Gynecology, Division of Gynecologic Oncology, Mayo Clinic, Rochester, MN USA; 774 International Institute for Molecular Oncology, Poznań, Poland; 775 Poznan University of Medical Sciences, Poznań, Poland; 776 Genomics and Proteomics Core Facility High Throughput Sequencing Unit, German Cancer Research Center (DKFZ), Heidelberg, Germany; 777 NCCS-VARI Translational Research Laboratory, National Cancer Centre Singapore, Singapore, Singapore; 778 Edison Family Center for Genome Sciences and Systems Biology, Washington University, St. Louis, MO USA; 779 MRC-University of Glasgow Centre for Virus Research, Glasgow, UK; 780 Department of Medical Informatics and Clinical Epidemiology, Division of Bioinformatics and Computational Biology, OHSU Knight Cancer Institute, Oregon Health and Science University, Portland, OR USA; 781 School of Electronic Information and Communications, Huazhong University of Science and Technology, Wuhan, China; 782 Department of Applied Mathematics and Statistics, Johns Hopkins University, Baltimore, MD USA; 783 Department of Cancer Genome Informatics, Graduate School of Medicine, Osaka University, Osaka, Japan; 784 School of Mathematics and Statistics, University of Sydney, Sydney, NSW Australia; 785 Ben May Department for Cancer Research, University of Chicago, Chicago, IL USA; 786 Department of Human Genetics, University of Chicago, Chicago, IL USA; 787 Tri-Institutional PhD Program in Computational Biology and Medicine, Weill Cornell Medicine, New York, NY USA; 788 The First Affiliated Hospital, Xi’an Jiaotong University, Xi’an, China; 789 Department of Medicine and Therapeutics, The Chinese University of Hong Kong, Shatin, NT, Hong Kong China; 790 Department of Biostatistics, The University of Texas MD Anderson Cancer Center, Houston, TX USA; 791 Duke-NUS Medical School, Singapore, Singapore; 792 Department of Surgery, Ruijin Hospital, Shanghai Jiaotong University School of Medicine, Shanghai, China; 793 School of Computing Science, University of Glasgow, Glasgow, UK; 794 Division of Orthopaedic Surgery, Oslo University Hospital, Oslo, Norway; 795 Eastern Clinical School, Monash University, Melbourne, VIC Australia; 796 Epworth HealthCare, Richmond, VIC Australia; 797 Department of Biostatistics and Computational Biology, Dana-Farber Cancer Institute and Harvard Medical School, Boston, MA USA; 798 Department of Biomedical Informatics, College of Medicine, The Ohio State University, Columbus, OH USA; 799 The Ohio State University Comprehensive Cancer Center (OSUCCC – James), Columbus, OH USA; 800 The University of Texas School of Biomedical Informatics (SBMI) at Houston, Houston, TX USA; 801 Department of Biostatistics, University of North Carolina at Chapel Hill, Chapel Hill, NC USA; 802 Department of Biochemistry and Molecular Genetics, Feinberg School of Medicine, Northwestern University, Chicago, IL USA; 803 Faculty of Medicine and Health, University of Sydney, Sydney, NSW Australia; 804 Department of Pathology, Erasmus Medical Center Rotterdam, Rotterdam, GD The Netherlands; 805 Division of Molecular Carcinogenesis, The Netherlands Cancer Institute, Amsterdam, CX The Netherlands; 806 Institute of Molecular Life Sciences and Swiss Institute of Bioinformatics, University of Zurich, Zurich, Switzerland

**Keywords:** Hardware and infrastructure, Computational platforms and environments, Data processing, Genome informatics

## Abstract

We present Butler, a computational tool that facilitates large-scale genomic analyses on public and academic clouds. Butler includes innovative anomaly detection and self-healing functions that improve the efficiency of data processing and analysis by 43% compared with current approaches. Butler enabled processing of a 725-terabyte cancer genome dataset from the Pan-Cancer Analysis of Whole Genomes (PCAWG) project in a time-efficient and uniform manner.

## Abstract (web summary)

Efficient, large-scale genomic analysis is facilitated on the cloud by a computational tool with error-diagnosing and self-healing capabilities.

## Main

Cloud computing offers easy and economical access to computational capacity at a scale that had previously been available to only the largest research institutions. To take advantage, large biological datasets are increasingly analyzed on various cloud computing platforms, using public, private and hybrid clouds^1^ with the aid of workflow systems. When employed in global projects, such systems must be flexible in their ability to operate in different environments, including academic clouds, to allow researchers to bring their computational pipelines to the data, especially in cases where the raw data themselves cannot be moved. The recently developed cloud-based scientific workflow frameworks Nextflow^2^, Toil^3^ and GenomeVIP^4^ focus their support largely on individual commercial cloud computing environments—mostly Amazon Web Services—and lack complete functionality for other major providers. This limits their use in studies that require multi-cloud operation due to practical and regulatory requirements^5,6^. Butler, in contrast, provides full support for operation on OpenStack-based commercial and academic clouds, Amazon Web Services, Microsoft Azure and Google Compute Platform, and can thus enable international collaborations involving the analysis of hundreds of thousands of samples where distributed cloud-based computation is pursued in different jurisdictions^5–7^.

**Fig. 1:**
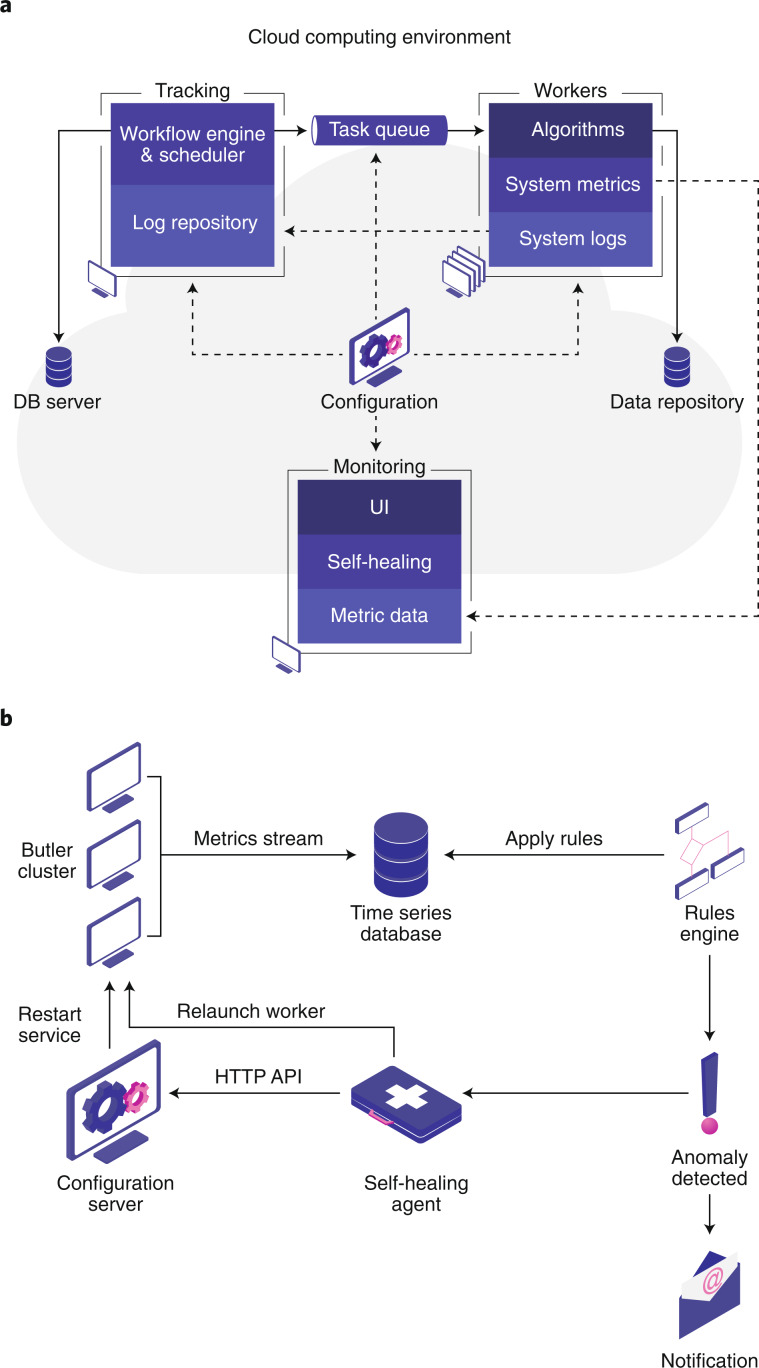
Butler framework architecture. **a**, The framework consists of several interconnected components, each running on a separate virtual machine (VM). See Methods and Supplementary Note 1 for details. **b**, Metrics flow from all VMs into a time series database. The self-healing agent detects anomalies and takes appropriate action. See Supplementary Note 1 for details. Solid arrows indicate information flow; dashed arrows indicate metrics flow; dashed-and-dotted arrows indicate configuration instructions.

A key lesson learned from large-scale projects including the PCAWG project^7^, which has pursued a study of 2,658 cancer genomes sequenced by the International Cancer Genome Consortium and the Cancer Genome Atlas, is that analysis of biological data of heterogeneous quality, generated at multiple locations with varying standard operating procedures, frequently suffers from artifacts that lead to many failures of computational jobs and that can considerably limit a project’s progress. Sequencing library artifacts, sample contamination and nonuniform sequencing coverage^8^ can cause data and software anomalies that challenge current workflows. Delays in recognizing and resolving these failures can notably affect data processing rate and increase project duration and costs. In contrast to previous tools, Butler provides an operational management toolkit that quickly discovers and resolves expected and unexpected failures (Fig. 1a,b and Supplementary Note 1).

The toolkit functions at two levels of granularity: host level and application level. Host-level operational management is facilitated via a health metrics system that collects system measurements at regular intervals from all deployed virtual machines (VMs). These metrics are aggregated and stored in a time-series database within Butler’s monitoring server. A set of graphical dashboards reports system health to users while supporting advanced querying capabilities for in-depth troubleshooting (Supplementary Fig. 8). Application-level monitoring is facilitated via systematic log collection (Supplementary Fig. 4) and extraction wherein the logs are stored in a queryable search index^9^. These tools provide multidimensional visibility into operational bottlenecks and error conditions as they occur, in a manner that is aggregated across hundreds of VMs. On top of these data, a rule-based anomaly detection engine defines normal operating conditions that, when breached, trigger handling routines that can notify the user by sending e-mail, Slack or Telegram messages, and enables automated restarting of offending workflows, underlying services or entire VMs, allowing the cluster to self-heal (Fig. 1b).

These monitoring and operational management capabilities set Butler apart from current scientific workflow frameworks^2–4,10^ (Supplementary Table 1), which do not contain anomaly detection modules and are therefore unable to automatically resolve key issues that frequently occur during large-scale analyses. For example, Butler’s operational modules are able to identify and resolve failures of the cloud workflow scheduler, workflows that run perpetually and never finish (indicative of underlying problems), and crashed and unresponsive VMs that, in practice, may prevent workflows from setting a failed status and thus would prevent triggering of error handling logic in other workflow systems.

These capabilities indeed enable highly efficient data processing in studies, such as PCAWG, where analyses are run by multiple groups at different times and on different clouds. Butler can invoke a variety of analysis algorithms, including genome alignment, variant calling and execution of R scripts. These can either be preinstalled or run as Docker^11^ images or Common Workflow Language (CWL)^12^ tools and workflows. Butler’s workflows accept parameters via JavaScript Object Notation (JSON) configuration files, which are stored in a database to maintain reproducibility. Workflow tasks scheduled for execution are deposited into a distributed task queue from which available worker nodes will pick them up, allowing analyses to be distributed over thousands of computing nodes. It is worth noting that for some small-scale projects executed over relatively short timelines, the increased complexity of setting up and running these monitoring systems may render Butler less practicable than simpler workflows.

We assessed Butler’s ability to facilitate large-scale analyses of patient genomes in the context of the PCAWG study, where Butler was deployed on 1,500 CPU cores, 5.5 terabytes of random access memory (RAM), 1 petabyte of shared storage and 40 terabytes of local solid-state drive storage. Using Butler, we implemented and successfully tested a genomic alignment workflow using BWA^13^, germline variant calling workflows based on FreeBayes^14^ (Supplementary Fig. 5) and Delly^15^, as well as several tools for somatic mutation calling, including Pindel^16^ and BRASS^17^. We carried out whole-genome variant discovery and joint genotyping of 90 million germline genetic variants (single nucleotide polymorphisms (SNPs), indels and structural variants) across a 725-terabyte dataset comprising the full PCAWG cohort (including samples that were later blacklisted) of 2,834 cancer patients^7^. Additionally, we performed sequence alignment and called both germline and somatic variants on 232 high-coverage prostate cancer tumor–normal sample pairs in the context of the PanProstate Cancer Group (PPCG) Consortium. We executed and successfully completed over 2.5 million computational jobs using 546,552 CPU hours. The management overhead of employing Butler for these analyses was less than 2% of the overall computational cost.

**Fig. 2:**
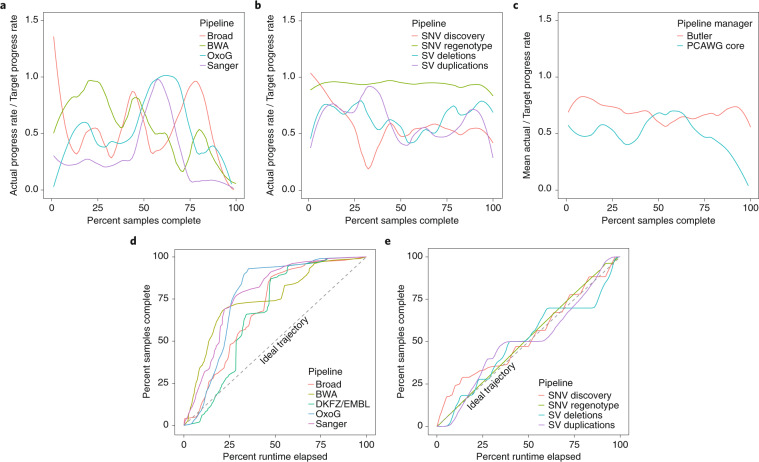
Butler performance comparison. **a**,**b**, Comparing the ratio of actual to target progress rates for core PCAWG pipelines (**a**) vs. Butler pipelines (**b**). See Methods for details. **c**, Mean actual/target progress rate ratio across pipelines for core PCAWG (mean 0.49) vs. Butler (mean 0.7) pipelines, each of which were run once over the entirety of PCAWG samples available to us. **d**,**e**, Progress rate uniformity of core PCAWG pipelines (**d**) vs. Butler (**e**). See Methods for details. In all panels the samples are arranged by their completion date. Runtime includes time spent on failed attempts. Comparison between Butler and core pipelines was facilitated in the context of the PCAWG. Similar comparison between Butler and other frameworks is presently impractical at this scale due to the high costs and complexity involved.

To assess Butler performance in the field, in comparison to other large-scale workflow systems, we compare the actually observed historical performance of Butler, recorded during PCAWG, against the performance of the ‘core’ somatic PCAWG consortium pipelines (Fig. 2), which represent the current state of the art in the field in terms of cloud software^7^ (on the basis of recency of development, scale of deployment, dataset size and analysis duration)—achieving nearly complete feature parity with several available cloud-based scientific workflow frameworks^2–4,10^ (Supplementary Table 1). These PCAWG pipelines used the same information technology infrastructure and computed over the same samples, but did not use Butler. Our metric to estimate the highest achievable processing rate for an analysis is defined as the smallest proportion of time required for processing 5% of all samples, which we refer to as the ‘target processing rate’. This is measured on the basis of the difference between the calendar completion date and time of the samples and the analysis start date, thus taking into account the time spent on failed and repeated runs and cluster downtime, which are major contributors to analysis duration. To establish how well a pipeline performs compared to its potential, we calculated the ratio of the actual processing rate to the target processing rate (Fig. 2a,b). Butler-operated pipelines were markedly closer to the target processing rate (mean actual/target rate ratio 0.696) than the core PCAWG pipelines (mean actual/target rate ratio 0.490) (Fig. 2c). Consequently, Butler-based analyses showed a duration 1.43 times the ideal target duration while core PCAWG pipelines showed a duration of 2.04 times the ideal target duration—43% longer. Additionally, core PCAWG pipelines exhibited a highly nonuniform processing rate (Fig. 2d) deviating 23.1% on average (minimum 0.0%, maximum 57.8%, s.d. 15.0%) from the ideally uniform trajectory of processing 1% of samples in 1% of analysis time, while Butler-based pipelines (Fig. 2e) performed in a substantially more uniform manner, deviating only 4.0% (minimum 0.0%, maximum 15.6%, s.d. 3.7%) over the same sample set on average (Methods). These timesaving and controlled execution abilities resulted in the adoption of Butler for genomics-oriented analyses in the context of the European Open Science Cloud (EOSC) Pilot (http://eoscpilot.eu) and its further adoption within PPGC (http://melbournebioinformatics.org.au/project/ppgc).

Butler can be generally applied to any large-scale analysis and could, for example, readily extend to studies such as GTEx (http://gtexportal.org), ENCODE (http://encodeproject.org) and the Human Cell Atlas Project (http://humancellatlas.org). A standard Butler workflow generically parallelizes R script execution across thousands of VMs, which will facilitate its use for other research contexts and other data types (including single-cell ‘omics’ data and microbiomes, for example).

We have developed Butler to meet the challenges of working with diverse cloud computing environments in the context of large-scale scientific data analyses. The operational management tools provided with Butler help overcome the key challenge that impacts analysis duration—the ability to autonomously detect, diagnose and address issues in a timely manner—thus allowing researchers to spend less time focusing on error conditions and considerably reduce analysis duration and cost. The comprehensive nature of the Butler toolkit sets it apart from current scientific workflow managers^2–4,10^ (Supplementary Table 1) by offering an efficient and scalable solution for modern global cloud-based big data analyses.

## Methods

### The Butler system

Overall, the Butler system is composed of four distinct subsystems. The Cluster Lifecycle Management is the first subsystem and deals with the task of creating and tearing down clusters on various clouds, including defining VMs, storage devices, network topology and network security rules. The second subsystem, Cluster Configuration Management, deals with configuration and software installation of all VMs in the cluster. The Workflow System is responsible for allowing users to define and run scientific workflows on the cloud. Finally, the Operational Management subsystem provides tools for ensuring continuous successful operation of the cluster, as well as for troubleshooting error conditions. Supplementary Note 1 contains an in-depth description of each of these subsystems and how they work within Butler, while the Installation Guide (http://butler.readthedocs.io/en/latest/installation.html) provides detailed instructions for how to set up the software.

### Butler deployment

Butler has been validated for production use on the EMBL-EBI Embassy Cloud (http://www.embassycloud.org), an academic cloud computing center that runs an OpenStack-based environment (Fig. 1). The Embassy Cloud has played a key role in the PCAWG project by donating substantial storage and cloud computing capacity over the course of 3 years. The total amount of resources dedicated to the project by the Embassy Cloud was as follows:

- 1 PB Isilon storage shared over NFS
- 1,500 computational cores
- 5.5 TB RAM
- 40 TB local solid-state drive storage
- 10-gigabit network

These resources have been used to host one of the six PCAWG data repositories that exist worldwide, as well as performing scientific analyses for the project. We have used Butler extensively on the Embassy Cloud to carry out the analyses for the PCAWG Germline Working Group. To deploy Butler on the 1,500-core cluster, we set up five different profiles of VMs, each playing several different roles (Supplementary Table 2).

Each profile was defined separately via Terraform and uses Saltstack roles for configuration. Users can check out the Butler github repository to their local machine, and once they install Terraform locally, they can fully commandeer the provisioning process from the local machine via Terraform.

The cluster is bootstrapped via the Salt-master VM. This VM is started first whenever the cluster needs to be recreated from scratch. The monitoring-server role is responsible for installing and configuring InfluxDB and other monitoring components, as well as registering them with Consul so that metrics can start being recorded. We also attach a 1-TB block storage volume for the metrics database so that it can survive cluster crashes and teardowns. If the monitoring server needs to be recreated, the block storage volume simply needs to be reattached to the new Monitoring Server VM.

The tracker VM is responsible for running various Airflow components, such as the Scheduler, Webserver and Flower. Additionally, we deploy the Butler tracker module to this VM, and thus the tracker VM acts as the main control point of the system from which analyses are launched and monitored. This VM additionally has the Elasticsearch role that designates it as the location of the Logstash and Elasticsearch components. To persist the search index, we attach an additional 1-TB block storage volume.

The job queue VM is responsible for hosting the RabbitMQ server, which holds all of the in-flight workflow tasks. Because the resources of the job queue are heavily taxed by communication with all of the worker VMs in the cluster, we do not assign any additional roles to this host.

The db-server is responsible for hosting most of the databases used by Butler. This VM runs an instance of PostgreSQL Server and hosts the Run Tracking DB, Airflow DB and Sample Tracking DB. The 1-TB block storage volume serves as the backing storage mechanism.

The worker VMs are the workhorses of the Butler cluster. For analyses by the PCAWG Germline Working Group, we employed 175 eight-core worker machines dedicated to running Butler workflows. The worker role ensures that Airflow client modules are installed and loaded on each worker. The germline role also loads the workflows and analyses that are relevant to the PCAWG Germline Working Group.

Because of the comprehensive nature of the Butler framework, which covers far more scope than a traditional workflow framework (provisioning, configuration management, operations management, anomaly detection, etc.), the setup and deployment of a Butler system are more complex than those of other workflow frameworks because multiple VMs need to be successfully set up and configured to interact with each other in a secure environment that is fit for sensitive information handling. Even though Butler features comprehensive documentation (http://butler.readthedocs.io), usage examples and automated deployment and configuration scripts, we recommend that the prospective user should ideally have a working understanding of cloud computing, server administration, networking, security, and other development operations (dev ops) concepts to make full use of the system. And while smaller-scale projects may benefit less from Butler’s state-of-the-art feature set owing to its increased complexity and learning curve, this feature set is imperative for enabling the success of current and future generations of large-scale bioinformatics computing on the cloud.

### PCAWG germline analyses

To assess Butler’s performance on real data, we carried out several large-scale data analyses using Butler on the Embassy Cloud and over the entirety of the 725 TB of raw PCAWG data, including the following:

- discovery of germline single nucleotide variants (SNVs) and small indels in normal genomes.
- genotyping of common SNVs occurring at minor allele frequency (MAF) >1% in the 1000 Genomes Project^18^.
- genotyping of germline SNVs and small indels in tumor and normal genomes (Supplementary Fig. 6).
- discovery and genotyping of structural variant deletions in tumor and normal genomes (Supplementary Fig. 7).
- discovery and genotyping of structural variant duplications in tumor and normal genomes (Supplementary Fig. 7).

Overall, most Butler workflows that carry out an analysis follow a similar structure (Supplementary Fig. 1): an analysis run is started, access to the sample is validated, the analysis steps are carried out (possibly with branching), and the analysis run is completed. Because of the largely common structure between workflows a large degree of code reuse is possible, and thus most of the methods reside in the workflow_common submodule of the Analysis Tracker and are invoked for each workflow.

Common variant genotyping was performed across the PCAWG cohort using a site list of 12 million variants occurring with at least 1% minor allele frequency within the 1000 Genomes Project^18^ phase 3 cohort, interrogating 34 billion sites overall. 130,152 computing hours were used to complete 70,850 workflow tasks for this analysis, with an additional 2,688 CPU hours used for cluster management overhead. Thus, management overhead accounted for 2% of the overall computational resource costs for this analysis. Using 1,000 cores, this analysis took less than 6 d to complete. Supplementary Fig. 2 shows a distribution of job runtimes by chromosome (runtimes highly correlate with chromosome length, *r* = 0.92). Using a site list of 60 million variants obtained from the FreeBayes Variant Discovery analysis, we used the Butler FreeBayes Workflow in genotyping mode to calculate genotypes at 170 billion genomic positions. 76,518 workflow tasks were completed using 302,071 CPU hours over the course of the analysis (10 d wall time), of which 5,040 CPU hours were cluster management overhead, accounting for 1.6% of total resource utilization.

244,889 deletions were evaluated across 5,668 samples (tumor and normal) for a total of 1,388,030,852 genomic sites genotyped. Overall wall time was 13 d, using 265,200 CPU hours with 6,240 CPU hours going to cluster management overhead—an overhead of 2.2%. 217,433 duplications were genotyped for each sample across 5,668 samples, for a total of 1,232,410,244 genomic variants genotyped. The wall time for this analysis was only 4.5 d, using 151,200 CPU hours during this time, with a management overhead of 2,160 h, for a total overhead of 1.4%. The comparatively low cluster management overhead has been accomplished by scaling up the cluster to 1,400 cores without the need for more management resources. Supplementary Fig. 3 shows a distribution of workflow run durations.

We carried out several analyses on a 725-TB dataset of 2,834 cancer patients’ genomic samples, consuming a total of 546,552 CPU hours. Each analysis took no longer than 2 weeks to complete and used only 1.5%–2.2% of the overall computing capacity for management overhead. On several occasions we were able detect large-scale cluster instability and program crashes using the Operational Management system and take corrective action with a minimal impact on overall productivity.

### Comparing Butler with the core PCAWG somatic pipelines

We evaluate the relative effectiveness of Butler-based pipelines in comparison to a set of pipelines operating under similar conditions and over the same dataset, namely the ‘core’ PCAWG somatic pipelines that have been used to accomplish genome alignment and somatic variant calling for the PCAWG Technical Working Group^7^. The core PCAWG pipeline set consists of five pipelines—BWA, Sanger, Broad, DKFZ/EMBL and OxoG detection—run over the course of 18 months over all samples in PCAWG. The Butler-based pipeline set consists of two pipelines—FreeBayes and Delly, used to accomplish four analyses: germline SNV discovery, germline SNV genotyping, germline structural variant deletion genotyping and germline structural variant duplication genotyping—also running over all samples in PCAWG (725 TB in total). We assessed and compared pipeline performance with respect to an estimated optimal performance (based on available hardware), as well as with respect to analysis progress uniformity in time.

For core PCAWG pipelines, we used the date of data upload to the official data repository as the most reliable sample completion date. However, approximately 25% of the DKFZ/EMBL pipeline results were uploaded in two batches on two separate days, and thus do not accurately represent the real analysis progress rate. For this reason, we excluded this pipeline from the optimal performance analysis. Butler sample completion dates are based on timestamps collected in Butler’s analysis tracking database.

Our assessment of pipeline performance is based on establishing an ‘optimal’ progress rate for a pipeline given a hardware allocation. We divided the sample set into 20 bins based on their completion time (each bin comprising 5% of all samples) and defined the optimal progress rate for each pipeline to be the smallest proportion of overall analysis time required to process all samples of a bin (scaled to a 1% rate).

$$r_{{\mathrm{opt}}} = \mathop {{{\mathrm{min}}}}\limits_{b \in {\mathrm{bins}}} \left\{ {{\mathrm{duration}}_b/{\mathrm{duration}}_{{\mathrm{total}}}/5} \right\}$$

We observed that the mean *r*_opt_ was significantly higher for Butler-based pipelines at 0.46 than for the core PCAWG pipelines at 0.13 (Supplementary Table 3). For each pipeline and each 1% of the samples under analysis, we then computed a metric *e* (for effectiveness) defined as the proportion of *r*_opt_ actually achieved.

$$e = \frac{{r_{{\mathrm{act}}}}}{{r_{{\mathrm{opt}}}}}$$

Comparing the core PCAWG and Butler pipelines with respect to *e* (Fig. 2a–c), we observed that effectiveness was on average lower for PCAWG pipelines (${\mu _{e_{\mathrm{PCAWG}}}} = {0.49}$) than for Butler pipelines ($\mu _{e_{\mathrm{Butler}}} = 0.70$). Assessing the expected analysis duration for the two sets of pipelines, we observed

$$d_{{\mathrm{PCAWG}}} = \frac{{100}}{{\mu _{e_{{\mathrm{PCAWG}}}}}} = 2.04d_{{\mathrm{opt}}}$$

$$d_{{\mathrm{Butler}}} = \frac{{100}}{{\mu _{e_{{\mathrm{Butler}}}}}} = 1.43d_{{\mathrm{opt}}}$$

$$d_{{\mathrm{PCAWG}}} = 1.43d_{{\mathrm{Butler}}}$$

Thus, the estimated duration for PCAWG pipelines was 43% longer than that for Butler-based pipelines.

We further compared core PCAWG pipelines with Butler pipelines on the basis of uniformity of rate of progress through an analysis. Given a constant resource allocation, an ideal analysis execution processes 1% of all samples in 1% of the analysis runtime. We divided the sample set into 100 equal-size bins and measured the percentage of overall analysis time spent processing each bin (Fig. 2d,e). Deviations from the diagonal indicate inefficiencies in data processing. Measuring this deviation, we observed that PCAWG pipelines deviated 23.1% from the diagonal on average (minimum 0.0%, maximum 57.8%, s.d. 15.0%) while Butler pipelines over the same sample set only deviated 4.0% (minimum 0.0%, maximum 15.6%, s.d. 3.7%) from the diagonal on average. This indicates that Butler pipelines are considerably less affected by various causes that slow an analysis (for example, job and infrastructure failures).

### Adapting Butler to new projects and domains

Butler is a highly general workflow framework, built on top of generic open source components that in principle can work with any data in any scientific domain, deploy onto over 20 cloud types, and work on any operating system, and it comprises a rich set of tools for installing and configuring software. Adapting Butler to a new application is straightforward. This process is described below.

Butler has a prebuilt library of workflows that focus on handling genomic data and can support a large variety of studies that are based on next-generation sequencing applications, such as variant discovery, common and rare variant association studies, cancer genome analysis, and expression quantitative trait locus (eQTL) mapping. Using one of these workflows is simply a matter of providing configuration values in JSON format for the underlying tools (such as, for example, FreeBayes, Delly, samtools^19^ or bcftools). Notably, Butler also supplies a generic workflow that allows execution of arbitrary R scripts across the entire Butler cluster. This powerful functionality can be used to facilitate a broad range of studies across disciplines, communities and analysis types, given the wide cross-community usage of R.

If the prebuilt workflows do not meet the users’ requirements as-is, they can be customized to adapt to arbitrary needs or entirely new workflows can be written. Each Butler workflow is a Python program, which typically contains only 100–200 lines of code. There are three principal avenues of developing new workflows that are suitable to a wide variety of users’ needs.

The easiest involves adapting tools that are already available as Docker images. Butler has prebuilt configurations for setting up all the infrastructure necessary to run Docker containers. The user only needs to wrap the Docker command line within existing boilerplate code that sets up access to the data that need to be analyzed. Once appropriate configuration parameters are supplied, Butler will be able to run the workflow seamlessly.

Only slightly more sophisticated is the setup of workflows that use CWL (Common Workflow Language) as a description language. Butler already has built-in functionality for installing and configuring cwl-runner, which is the reference implementation of CWL. To set up a new workflow that uses CWL within Butler, users need to prepare an appropriate JSON parameter file according to the CWL definition. This is accomplished via Butler’s configuration functionality. The genome alignment and somatic variant calling workflows that accompany the Butler framework already provide full functionality in this regard and can be used as examples by new users. Because a number of workflows from varying scientific fields have already been described with CWL, this approach opens up a relatively straightforward avenue for adopting Butler in a wide variety of additional studies.

Potentially the most complex, but also the most powerful, way of authoring new workflows is writing them using the native constructs of the underlying Apache Airflow workflow framework. This approach provides the users with all of the power of the Python language and extended library, as well as the prebuilt Airflow components for interacting with a wide variety of distributed systems and engines, such as HDFS, Apache Spark, Apache Cassandra, various databases such as PostgreSQL and SQLite, email engines and many more. Several of the prebuilt Butler workflows, such as the FreeBayes, Delly and R workflow, use this approach, and users can employ these as templates for new workflows built in this style.

Because of the wide variety of workflow authoring and customization styles available, the existing examples, and the generic nature of the underlying open source components, applying Butler to new projects and analysis domains can be accomplished with minimal efforts and at a complexity level that is matched to the requirements of the project. Individual steps of the workflow can be easily debugged and tested on the local machine without the need to deploy to any cloud, using Python’s extensive testing and debugging functionality. The typical life cycle for developing a new workflow is a few hours to a few days long and is usually much shorter than a week. Because new projects frequently require the installation and configuration of new software packages, Butler has integrated a full-featured configuration management solution called Saltstack that is used to set up and configure Butler internals and also any additional software required by the user for their project. Recipes for configuring dozens of software packages are already included with the Butler system, and hundreds more are available as community contributions to the Saltstack project. Arbitrary new configurations can be defined by the user to meet their custom requirements. To support this the user would typically set up a new Github repository that acts as a customization layer on top of the core Butler configurations. Within this custom repository, users can define new configuration recipes or override the behavior of the pre-existing Butler settings depending on the needs of their scientific project. We provide several examples of such repositories under ‘Code availability’ to help users become familiar with Butler.

### Statistics

No formal sample size and power calculations were performed as we made use of all 5,668 of the samples available to us via the PCAWG consortium. The analyses in Fig. 2, performed over the entirety of PCAWG samples available to us, were run once (rather than multiple times) owing to the multi-year nature and high costs of the PCAWG project.

### Ethical compliance

The authors have complied with all of the relevant ethical regulations with regards to the subjects described in this manuscript.

### Reporting Summary

Further information on research design is available in the Nature Research Reporting Summary linked to this article.

## Online content

Any methods, additional references, Nature Research reporting summaries, source data, extended data, supplementary information, acknowledgements, peer review information; details of author contributions and competing interests; and statements of data and code availability are available at 10.1038/s41587-019-0360-3.

## Supplementary Information

### Integrated supplementary information

Supplementary Figure 1 Freebayes workflow. Freebayes workflow can be used for small variant discovery and genotyping and splits into tasks by chromosome, where each task can run in parallel (not all tasks are visible in figure to save space). Workflow is started and ended by standard start_analysis_run and end_analysis_run that keep track of Analysis state. validate_sample makes sure that access to the data is available.

Supplementary Figure 2 Freebayes task durations. Boxplot of freebayes task durations during the SNV genotyping stage across 5668 samples. Durations are highly correlated with chromosome length (Pearson’s r=0.92). n=5668 biologically independent samples Boxplot center line corresponds to the median, lower and upper hinges to the 25%th and 75%th percentiles, and whiskers to +- 1.5 Interquartile range from the hinges. The experiment was performed once.

Supplementary Figure 3 Delly workflow durations. (**a**) Distribution of Delly workflow durations for genotyping of 244,889 germline deletions across 5668 PCAWG samples. (**b**) Distribution of Delly workflow durations for genotyping of 217,433 germline duplications across 5668 PCAWG samples. n=5668 biologically independent samples. The experiment was performed once.

Supplementary Figure 4 Analysis Tracker UML diagram. The Analysis Tracker consists of four entities that are necessary for keeping track of the state of scientific analyses run in Butler. The Workflow object keeps a registry of known workflows and their attributes. The Analysis object keeps track of analyses that are being performed. An Analysis Run represents an instance of running a particular workflow under a particular analysis on a particular sample. Configuration objects keep track of the parameters supplied to the workflow invocation.

Supplementary Figure 5 Analysis Run state transitions. Each Analysis Run keeps track of its state and has a set of rules governing allowable state transitions. A Run is created in the Ready state from which it may be scheduled for execution. Once the corresponding workflow task is picked up for execution it is transitioned to In-Progress. Upon successful completion it is marked Completed. At any point a failure may put this run in an Error state from which it can recover only to the Ready state to initiate a re-execution of the corresponding workflow.

Supplementary Figure 6 Hierarchical tri-level configuration. Configuration can be applied at three levels of granularity within Butler - Workflow, Analysis, and Analysis Run. Each higher level configuration may override and augment the configurations supplied at lower levels. At runtime all three levels of configuration are resolved into an “effective configuration”, which is then applied for execution.

Supplementary Figure 7 Butler compute cluster performance metrics during germline deletion genotyping for PCAWG. (**a**) Overall load per VM that is part of the Butler cluster - shows no load prior to analysis kick-off, then steady load throughout the analysis, and drop-off in load at the end when VMs start running out of work. (**b**) CPU profile shows highly variable CPU utilization that is typical of Delly executions. (**c**) Memory profile is stable and similar between all VMs that are running the analysis. Similar measurements have been observed over the other 5 analyses performed with Butler during PCAWG, although the exact pattern of CPU and Memory utilization is dependent on the algorithms that comprise the workflow being executed.

Supplementary Figure 8 SQL Database state monitoring dashboard. SQL Database health can be ascertained from logs harvested on the database server. (**a**) 75th, 99th, and 99.5th percentile of query response times. (**b**) Count queries by type. (**c**) Database READ and WRITE counts. (**d**) Data throughput in and out. These measurements were collected over a single 2-hour run of the software and serve as an example of visualization capabilities, not an indication of typical database performance.

### Supplementary information

Supplementary Materials Supplementary Figures 1–8, Supplementary Tables 1–3 and Supplementary Note 1

Reporting Summary

## Data Availability

PCAWG’s final callsets, somatic and germline variant calls, mutational signatures, subclonal reconstructions, transcript abundance, splice calls and other core data generated by the ICGC/TCGA Pan-cancer Analysis of Whole Genomes Consortium is described in ref. ^7^ and available for download at https://dcc.icgc.org/releases/PCAWG. Additional information on accessing the data, including raw read files, can be found at https://docs.icgc.org/pcawg/data/. In accordance with the data access policies of the ICGC and TCGA projects, most molecular, clinical and specimen data are in an open tier that does not require access approval. To access potentially identifying information, such as germline alleles and underlying sequencing data, researchers will need to apply to the TCGA Data Access Committee (DAC) via dbGaP (https://dbgap.ncbi.nlm.nih.gov/aa/wga.cgi?page=login) for access to the TCGA portion of the dataset and to the ICGC Data Access Compliance Office (DACO; http://icgc.org/daco) for access to the ICGC portion. In addition, to access somatic single nucleotide variants derived from TCGA donors, researchers will also need to obtain dbGaP authorization.

## Code availability

The source code for Butler is freely available at http://github.com/llevar/butler under the GPL v3.0 license.

The project-specific deployment settings, configurations, analysis definitions, and workflows are available at the following:

PCAWG Germline Project: https://github.com/llevar/pcawg-germline

EOSC Pilot: https://github.com/llevar/eosc_pilot

Pan-Prostate Cancer Group: https://github.com/llevar/pan-prostate

The R source code for the analysis is available at https://github.com/llevar/butler_perf_analysis.

The core computational pipelines used by the PCAWG Consortium for alignment, quality control and variant calling are available to the public at https://dockstore.org/search?search=pcawg under the GNU General Public License v3.0, which allows for reuse and distribution.
