# Publisher Correction: Butler enables rapid cloud-based analysis of thousands of human genomes

**DOI:** 10.1038/s41587-020-0448-9

**Published:** 2020-02-12

**Authors:** Sergei Yakneen, Sebastian M. Waszak, PCAWG Technical Working Group, Sergei Yakneen, Brice Aminou, Javier Bartolome, Keith A. Boroevich, Rich Boyce, Angela N. Brooks, Alex Buchanan, Ivo Buchhalter, Adam P. Butler, Niall J. Byrne, Andy Cafferkey, Peter J. Campbell, Zhaohong Chen, Sunghoon Cho, Wan Choi, Peter Clapham, Brandi N. Davis-Dusenbery, Francisco M. De La Vega, Jonas Demeulemeester, Michelle T. Dow, Lewis Jonathan Dursi, Juergen Eils, Roland Eils, Kyle Ellrott, Claudiu Farcas, Francesco Favero, Nodirjon Fayzullaev, Vincent Ferretti, Paul Flicek, Nuno A. Fonseca, Josep Ll. Gelpi, Gad Getz, Bob Gibson, Robert L. Grossman, Olivier Harismendy, Allison P. Heath, Michael C. Heinold, Julian M. Hess, Oliver Hofmann, Jongwhi H. Hong, Thomas J. Hudson, Barbara Hutter, Carolyn M. Hutter, Daniel Hübschmann, Seiya Imoto, Sinisa Ivkovic, Seung-Hyup Jeon, Wei Jiao, Jongsun Jung, Rolf Kabbe, Andre Kahles, Jules N. A. Kerssemakers, Hyung-Lae Kim, Hyunghwan Kim, Jihoon Kim, Youngwook Kim, Kortine Kleinheinz, Michael Koscher, Antonios Koures, Milena Kovacevic, Chris Lawerenz, Ignaty Leshchiner, Jia Liu, Dimitri Livitz, George L. Mihaiescu, Sanja Mijalkovic, Ana Mijalkovic Lazic, Satoru Miyano, Naoki Miyoshi, Hardeep K. Nahal-Bose, Hidewaki Nakagawa, Mia Nastic, Steven J. Newhouse, Jonathan Nicholson, Brian D. O’Connor, David Ocana, Kazuhiro Ohi, Lucila Ohno-Machado, Larsson Omberg, B. F Francis Ouellette, Nagarajan Paramasivam, Marc D. Perry, Todd D. Pihl, Manuel Prinz, Montserrat Puiggròs, Petar Radovic, Keiran M. Raine, Esther Rheinbay, Mara Rosenberg, Romina Royo, Gunnar Rätsch, Gordon Saksena, Matthias Schlesner, Solomon I. Shorser, Charles Short, Heidi J. Sofia, Jonathan Spring, Lincoln D. Stein, Adam J. Struck, Grace Tiao, Nebojsa Tijanic, David Torrents, Peter Van Loo, Miguel Vazquez, David Vicente, Jeremiah A. Wala, Zhining Wang, Sebastian M. Waszak, Joachim Weischenfeldt, Johannes Werner, Ashley Williams, Youngchoon Woo, Adam J. Wright, Qian Xiang, Liming Yang, Denis Yuen, Christina K. Yung, Junjun Zhang, Jan O. Korbel, Michael Gertz, Jan O. Korbel, PCAWG Consortium

**Affiliations:** 1 European Molecular Biology Laboratory (EMBL), Genome Biology Unit, Heidelberg, Germany; 2 Institute of Computer Science, Heidelberg University, Heidelberg, Germany; 3 Institute of Computer Science, Heidelberg University, Heidelberg, Germany; 4 EMBL, European Bioinformatics Institute (EMBL-EBI), Hinxton, UK; 5 Institute of Computer Science, Heidelberg University, Heidelberg, Germany; 6 Present Address: Sophia Genetics SA, Saint Sulpice, Switzerland; 7 Genome Informatics Program, Ontario Institute for Cancer Research, Toronto, Ontario Canada; 8 Barcelona Supercomputing Center (BSC), Barcelona, Spain; 9 Laboratory for Medical Science Mathematics, RIKEN Center for Integrative Medical Sciences, Yokohama, Kanagawa Japan; 10 RIKEN Center for Integrative Medical Sciences, Yokohama, Kanagawa Japan; 11 Broad Institute of MIT and Harvard, Cambridge, MA USA; 12 Dana-Farber Cancer Institute, Boston, MA USA; 13 University of California Santa Cruz, Santa Cruz, CA USA; 14 Oregon Health and Science University, Portland, OR USA; 15 Division of Theoretical Bioinformatics, German Cancer Research Center (DKFZ), Heidelberg, Germany; 16 Heidelberg Center for Personalized Oncology (DKFZ-HIPO), German Cancer Research Center, Heidelberg, Germany; 17 Institute of Pharmacy and Molecular Biotechnology and BioQuant, Heidelberg University, Heidelberg, Germany; 18 Wellcome Sanger Institute, Wellcome Genome Campus, Hinxton, Cambridge UK; 19 Department of Haematology, University of Cambridge, Cambridge, UK; 20 University of California San Diego, San Diego, CA USA; 21 PDXen Biosystems Inc, Seoul, South Korea; 22 Electronics and Telecommunications Research Institute, Daejeon, South Korea; 23 Seven Bridges Genomics, Charlestown, MA USA; 24 Annai Systems, Inc, Carlsbad, CA USA; 25 Department of Biomedical Data Science, Stanford University School of Medicine, Stanford, CA USA; 26 Department of Genetics, Stanford University School of Medicine, Stanford, CA USA; 27 Departments of Genetics and Biomedical Data Science, Stanford University School of Medicine, Stanford, CA USA; 28 University of Leuven, Leuven, Belgium; 29 The Francis Crick Institute, London, UK; 30 Computational Biology Program, Ontario Institute for Cancer Research, Toronto, Ontario Canada; 31 The Hospital for Sick Children, Toronto, Ontario Canada; 32 Heidelberg University, Heidelberg, Germany; 33 New BIH Digital Health Center, Berlin Institute of Health (BIH) and Charité – Universitätsmedizin Berlin, Berlin, Germany; 34 Rigshospitalet, Copenhagen, Denmark; 35 Department of Biochemistry and Molecular Medicine, University of Montreal, Montreal, Quebec Canada; 36 CIBIO/InBIO— Research Center in Biodiversity and Genetic Resources, Universidade do Porto, Vairão, Portugal; 37 Department Biochemistry and Molecular Biomedicine, University of Barcelona, Barcelona, Spain; 38 Center for Cancer Research, Massachusetts General Hospital, Boston, MA USA; 39 Department of Pathology, Massachusetts General Hospital, Boston, MA USA; 40 Harvard Medical School, Boston, MA USA; 41 University of Chicago, Chicago, IL USA; 42 Division of Biomedical Informatics, Department of Medicine, & Moores Cancer Center, UC San Diego School of Medicine, San Diego, CA USA; 43 Children’s Hospital of Philadelphia, Philadelphia, PA USA; 44 Massachusetts General Hospital Center for Cancer Research, Charlestown, MA USA; 45 University of Melbourne Centre for Cancer Research, University of Melbourne, Melbourne, Victoria Australia; 46 Syntekabio Inc, Daejeon, South Korea; 47 AbbVie, North Chicago, IL USA; 48 Genomics Program, Ontario Institute for Cancer Research, Toronto, Ontario Canada; 49 German Cancer Consortium (DKTK), Heidelberg, Germany; 50 Heidelberg Center for Personalized Oncology (DKFZ-HIPO), German Cancer Research Center (DKFZ), Heidelberg, Germany; 51 National Center for Tumor Diseases (NCT) Heidelberg, Heidelberg, Germany; 52 National Human Genome Research Institute, National Institutes of Health, Bethesda, MD USA; 53 Department of Pediatric Immunology, Hematology and Oncology, University Hospital, Heidelberg, Germany; 54 German Cancer Research Center (DKFZ), Heidelberg, Germany; 55 Heidelberg Institute for Stem Cell Technology and Experimental Medicine (HI-STEM), Heidelberg, Germany; 56 Institute of Medical Science, University of Tokyo, Tokyo, Japan; 57 Seven Bridges, Charlestown, MA USA; 58 Genome Integration Data Center, Syntekabio, Inc, Daejeon, South Korea; 59 Computational Biology Center, Memorial Sloan Kettering Cancer Center, New York, NY USA; 60 ETH Zurich, Department of Biology, Zurich, Switzerland; 61 ETH Zurich, Department of Computer Science, Zurich, Switzerland; 62 SIB Swiss Institute of Bioinformatics, Lausanne, Switzerland; 63 University Hospital Zurich, Zurich, Switzerland; 64 Department of Biochemistry, College of Medicine, Ewha Womans University, Seoul, South Korea; 65 Health Sciences Department of Biomedical Informatics, University of California San Diego, La Jolla, CA USA; 66 Department of Health Sciences and Technology, Sungkyunkwan University School of Medicine, Seoul, South Korea; 67 Samsung Genome Institute, Seoul, South Korea; 68 Functional and Structural Genomics, German Cancer Research Center (DKFZ), Heidelberg, Germany; 69 Leidos Biomedical Research, Inc, McLean, VA USA; 70 Sage Bionetworks, Seattle, WA USA; 71 Genome Informatics, Ontario Institute for Cancer Research, Toronto, Ontario Canada; 72 Department of Cell and Systems Biology, University of Toronto, Toronto, Ontario Canada; 73 Department of Radiation Oncology, University of California San Francisco, San Francisco, CA USA; 74 CSRA Incorporated, Fairfax, VA USA; 75 Barcelona Supercomputing Center, Barcelona, Spain; 76 Massachusetts General Hospital, Boston, MA USA; 77 Department of Biology, ETH Zurich, Zurich, Switzerland; 78 Department of Computer Science, ETH Zurich, Zurich, Switzerland; 79 Weill Cornell Medical College, New York, NY USA; 80 Bioinformatics and Omics Data Analytics, German Cancer Research Center (DKFZ), Heidelberg, Germany; 81 Department of Molecular Genetics, University of Toronto, Toronto, Ontario Canada; 82 Institució Catalana de Recerca i Estudis Avançats (ICREA), Barcelona, Spain; 83 National Cancer Institute, National Institutes of Health, Bethesda, MD USA; 84 Finsen Laboratory and Biotech Research & Innovation Centre (BRIC), University of Copenhagen, Copenhagen, Denmark; 85 Department of Urology, Charité Universitätsmedizin Berlin, Berlin, Germany; 86 Department of Biological Oceanography, Leibniz Institute of Baltic Sea Research, Rostock, Germany; 87 Ontario Institute for Cancer Research, Toronto, Ontario Canada

**Keywords:** Hardware and infrastructure, Computational platforms and environments, Data processing, Genome informatics

Correction to: *Nature Biotechnology* 10.1038/s41587-019-0360-3, published online 5 February 2020.

This paper was originally published under standard Springer Nature copyright (© The Author(s), under exclusive licence to Springer Nature America, Inc.). It is now available as an open-access paper under a Creative Commons Attribution 4.0 International license.

**PCAWG Technical Working Group**

Sergei Yakneen, Brice Aminou, Javier Bartolome, Keith A. Boroevich, Rich Boyce, Angela N. Brooks, Alex Buchanan, Ivo Buchhalter, Adam P. Butler, Niall J. Byrne, Andy Cafferkey, Peter J. Campbell, Zhaohong Chen, Sunghoon Cho, Wan Choi, Peter Clapham, Brandi N. Davis-Dusenbery, Francisco M. De La Vega, Jonas Demeulemeester, Michelle T. Dow, Lewis Jonathan Dursi, Juergen Eils, Roland Eils, Kyle Ellrott, Claudiu Farcas, Francesco Favero, Nodirjon Fayzullaev, Vincent Ferretti, Paul Flicek, Nuno A. Fonseca, Josep Ll. Gelpi, Gad Getz, Bob Gibson, Robert L. Grossman, Olivier Harismendy, Allison P. Heath, Michael C. Heinold, Julian M. Hess, Oliver Hofmann, Jongwhi H. Hong, Thomas J. Hudson, Barbara Hutter, Carolyn M. Hutter, Daniel Hübschmann, Seiya Imoto, Sinisa Ivkovic, Seung-Hyup Jeon, Wei Jiao, Jongsun Jung, Rolf Kabbe, Andre Kahles, Jules N. A. Kerssemakers, Hyung-Lae Kim, Hyunghwan Kim, Jihoon Kim, Youngwook Kim, Kortine Kleinheinz, Michael Koscher, Antonios Koures, Milena Kovacevic, Chris Lawerenz, Ignaty Leshchiner, Jia Liu, Dimitri Livitz, George L. Mihaiescu, Sanja Mijalkovic, Ana Mijalkovic Lazic, Satoru Miyano, Naoki Miyoshi, Hardeep K. Nahal-Bose, Hidewaki Nakagawa, Mia Nastic, Steven J. Newhouse, Jonathan Nicholson, Brian D. O’Connor, David Ocana, Kazuhiro Ohi, Lucila Ohno-Machado, Larsson Omberg, B. F Francis Ouellette, Nagarajan Paramasivam, Marc D. Perry, Todd D. Pihl, Manuel Prinz, Montserrat Puiggròs, Petar Radovic, Keiran M. Raine, Esther Rheinbay, Mara Rosenberg, Romina Royo, Gunnar Rätsch, Gordon Saksena, Matthias Schlesner, Solomon I. Shorser, Charles Short, Heidi J. Sofia, Jonathan Spring, Lincoln D. Stein, Adam J. Struck, Grace Tiao, Nebojsa Tijanic, David Torrents, Peter Van Loo, Miguel Vazquez, David Vicente, Jeremiah A. Wala, Zhining Wang, Sebastian M. Waszak, Joachim Weischenfeldt, Johannes Werner, Ashley Williams, Youngchoon Woo, Adam J. Wright, Qian Xiang, Liming Yang, Denis Yuen, Christina K. Yung, Junjun Zhang and Jan O. Korbel

