# Supplementary figures and images for: Butler enables rapid cloud-based analysis of thousands of human genomes
Source: Nat Biotechnol. 2020 Feb 5;38(3):288–92. doi: 10.1038/s41587-019-0360-3 (PMC7062635; doi:10.1038/s41587-019-0360-3)

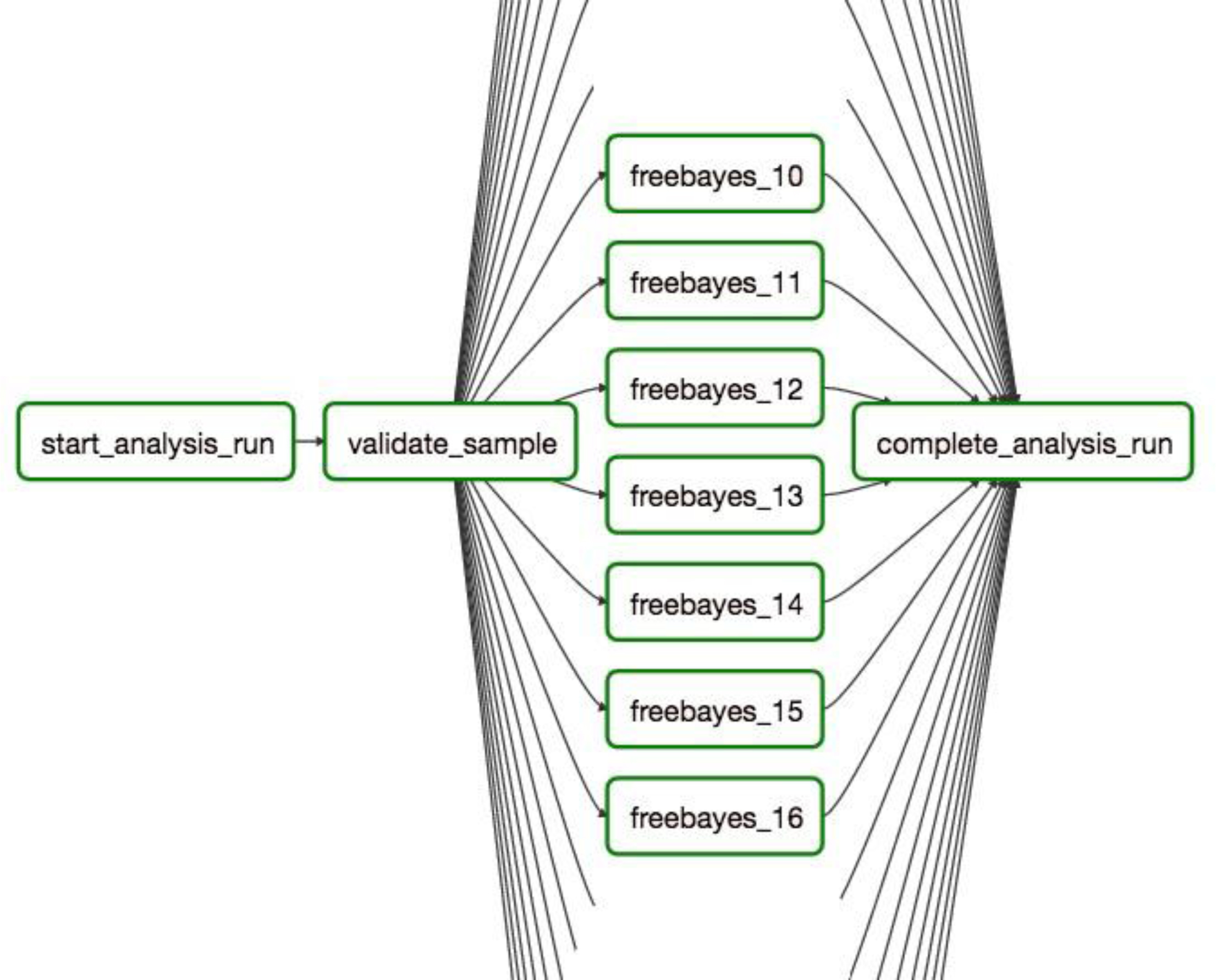

Supplement: Freebayes workflow. — Freebayes workflow can be used for small variant discovery and genotyping and splits into tasks by chromosome, where each task can run in parallel (not all tasks are visible in figure to save space). Workflow is started and ended by standard start_analysis_run and end_analysis_run that keep track of Analysis state. validate_sample makes sure that access to the data is available. [file 41587_2019_360_Fig3_ESM.jpg]

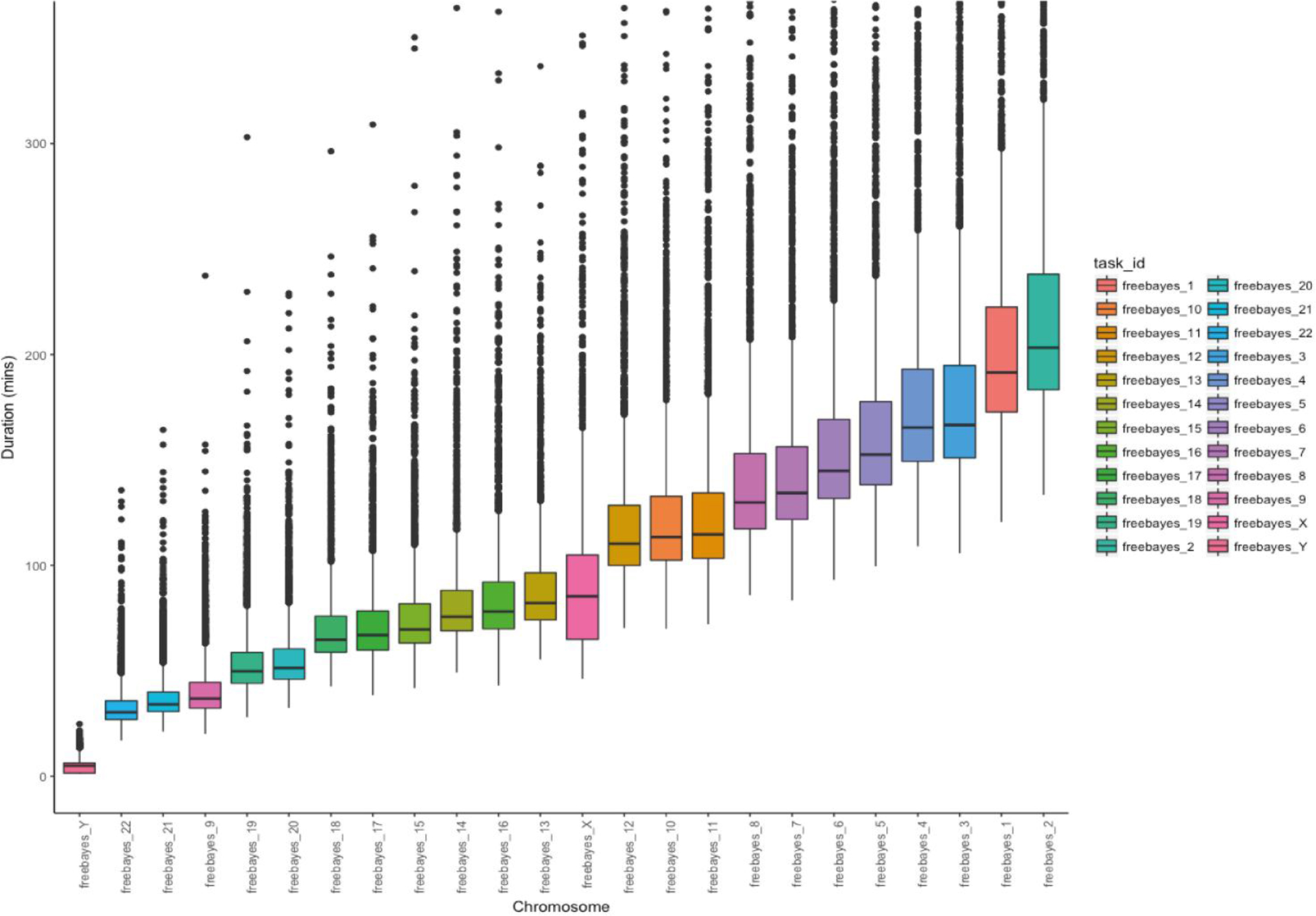

Supplement: Freebayes task durations. — Boxplot of freebayes task durations during the SNV genotyping stage across 5668 samples. Durations are highly correlated with chromosome length (Pearson’s r=0.92). n=5668 biologically independent samples Boxplot center line corresponds to the median, lower and upper hinges to the 25%th and 75%th percentiles, and whiskers to +- 1.5 Interquartile range from the hinges. The experiment was performed once. [file 41587_2019_360_Fig4_ESM.jpg]

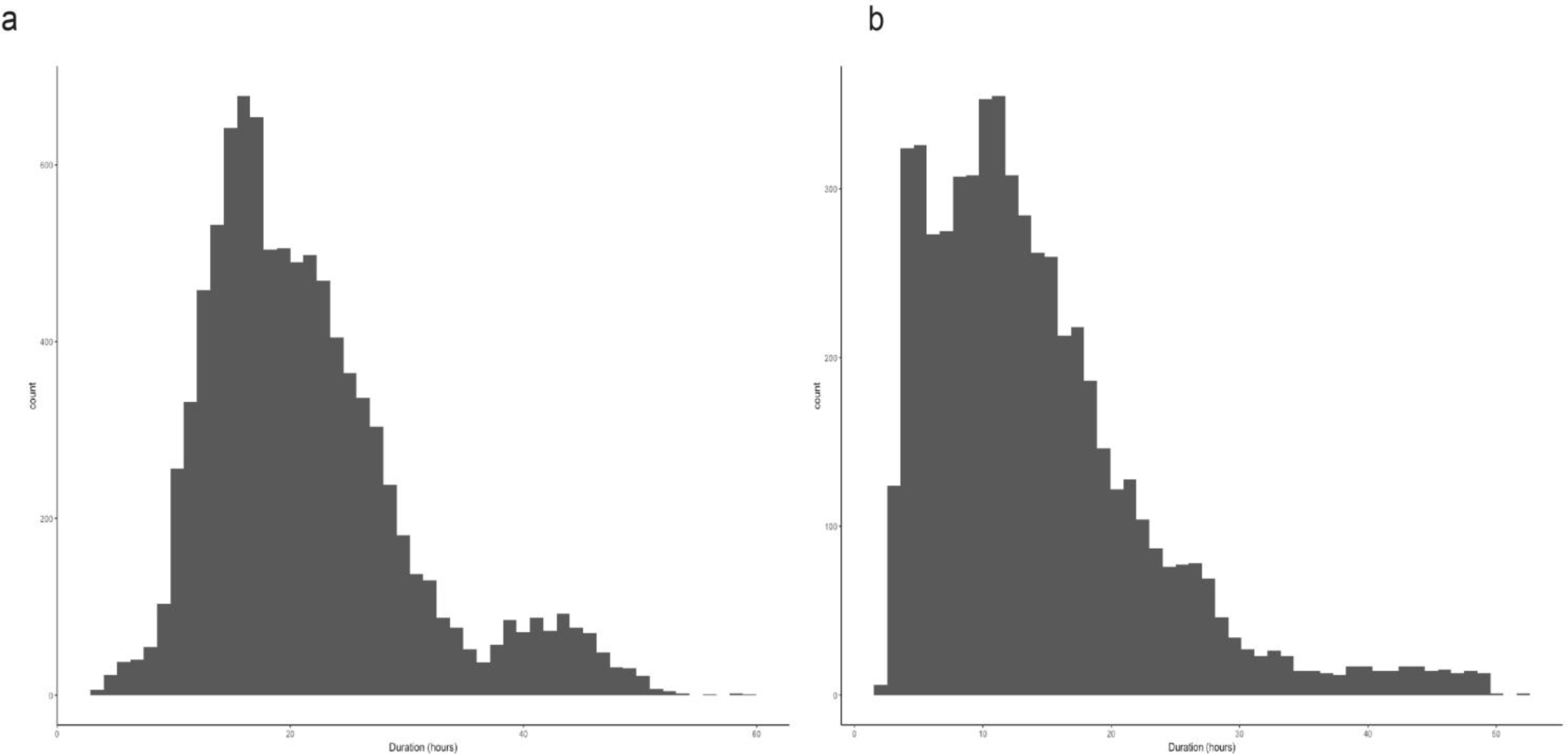

Supplement: Delly workflow durations. — (a) Distribution of Delly workflow durations for genotyping of 244,889 germline deletions across 5668 PCAWG samples. (b) Distribution of Delly workflow durations for genotyping of 217,433 germline duplications across 5668 PCAWG samples. n=5668 biologically independent samples. The experiment was performed once. [file 41587_2019_360_Fig5_ESM.jpg]

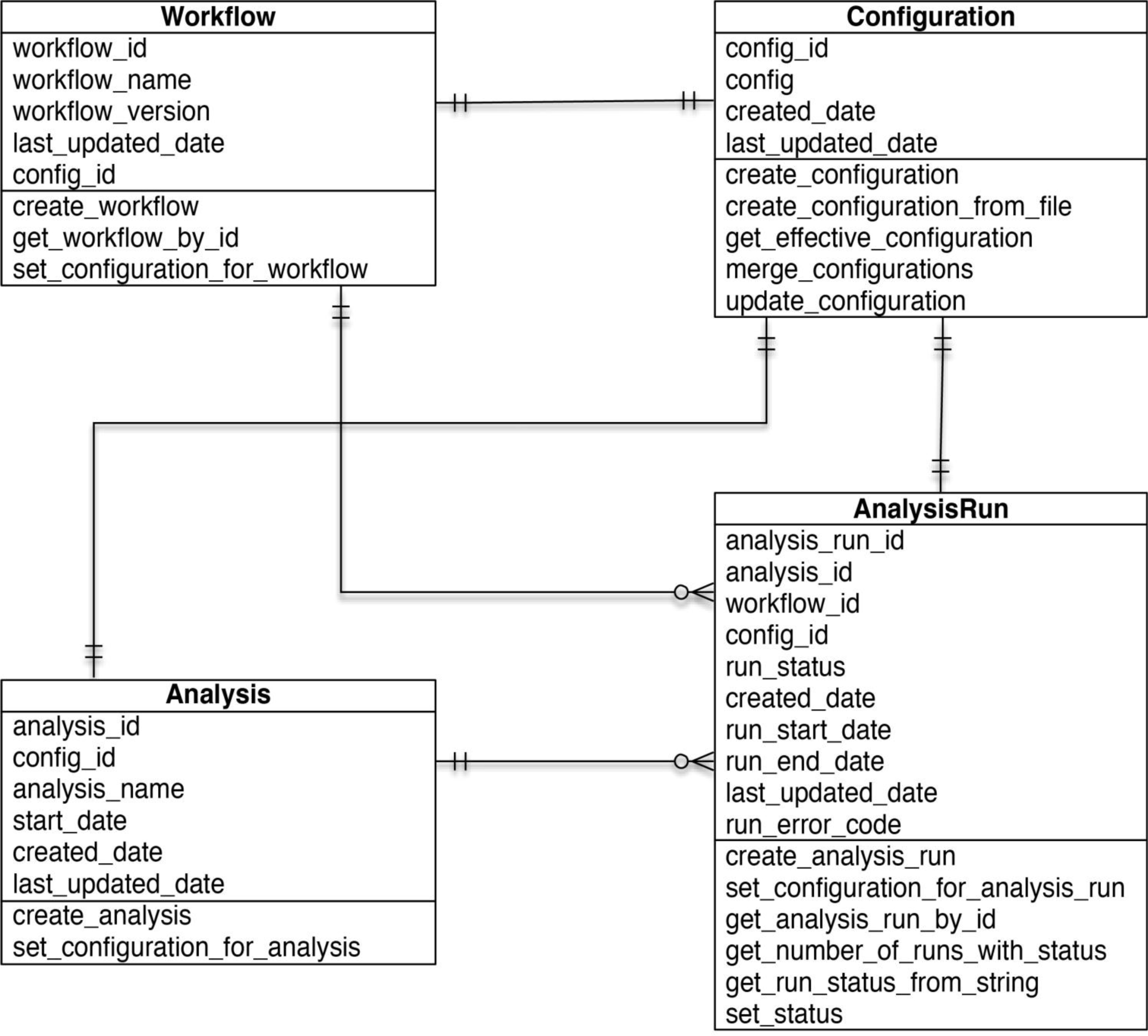

Supplement: Analysis Tracker UML diagram. — The Analysis Tracker consists of four entities that are necessary for keeping track of the state of scientific analyses run in Butler. The Workflow object keeps a registry of known workflows and their attributes. The Analysis object keeps track of analyses that are being performed. An Analysis Run represents an instance of running a particular workflow under a particular analysis on a particular sample. Configuration objects keep track of the parameters supplied to the workflow invocation. [file 41587_2019_360_Fig6_ESM.jpg]

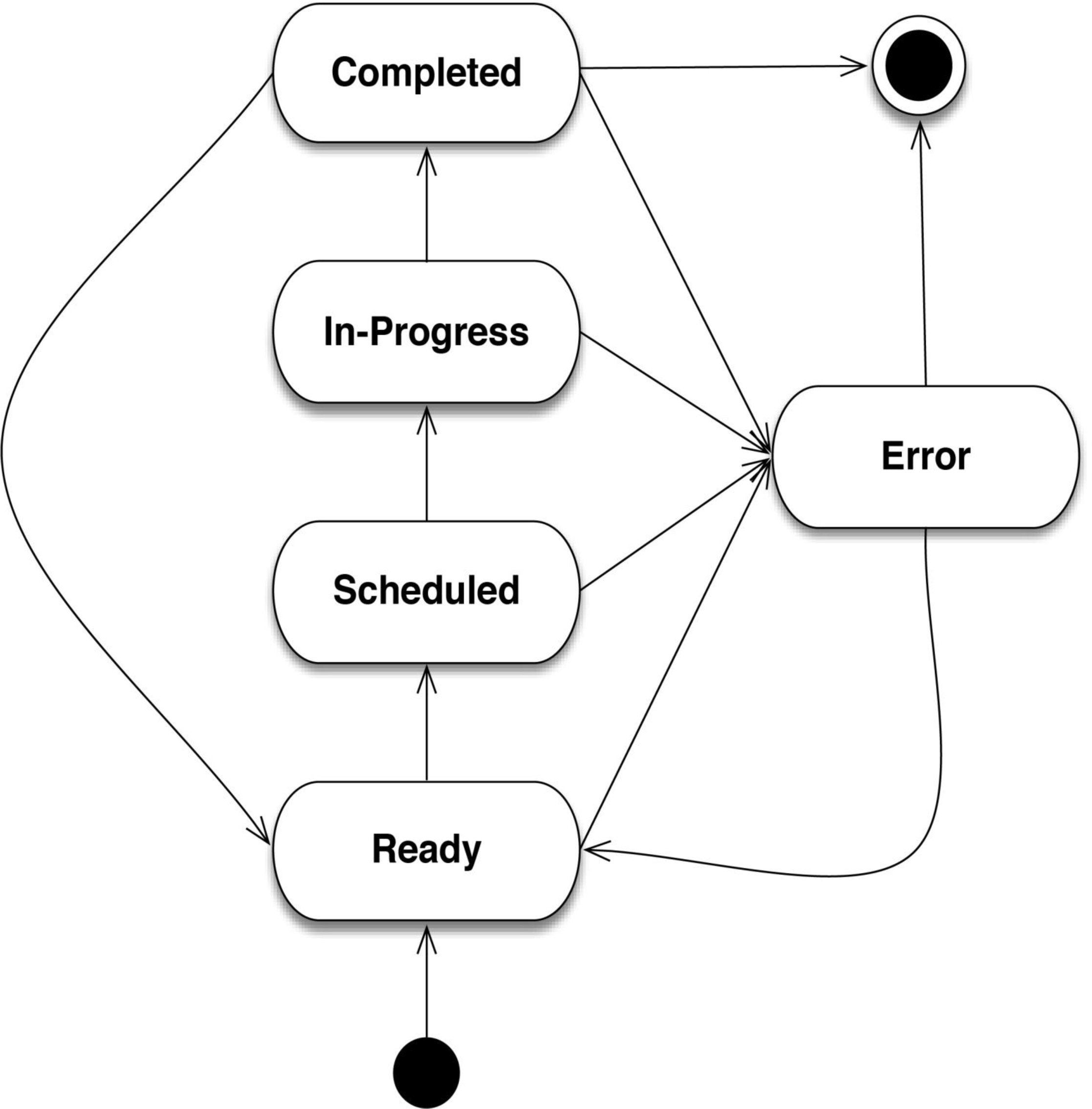

Supplement: Analysis Run state transitions. — Each Analysis Run keeps track of its state and has a set of rules governing allowable state transitions. A Run is created in the Ready state from which it may be scheduled for execution. Once the corresponding workflow task is picked up for execution it is transitioned to In-Progress. Upon successful completion it is marked Completed. At any point a failure may put this run in an Error state from which it can recover only to the Ready state to initiate a re-execution of the corresponding workflow. [file 41587_2019_360_Fig7_ESM.jpg]

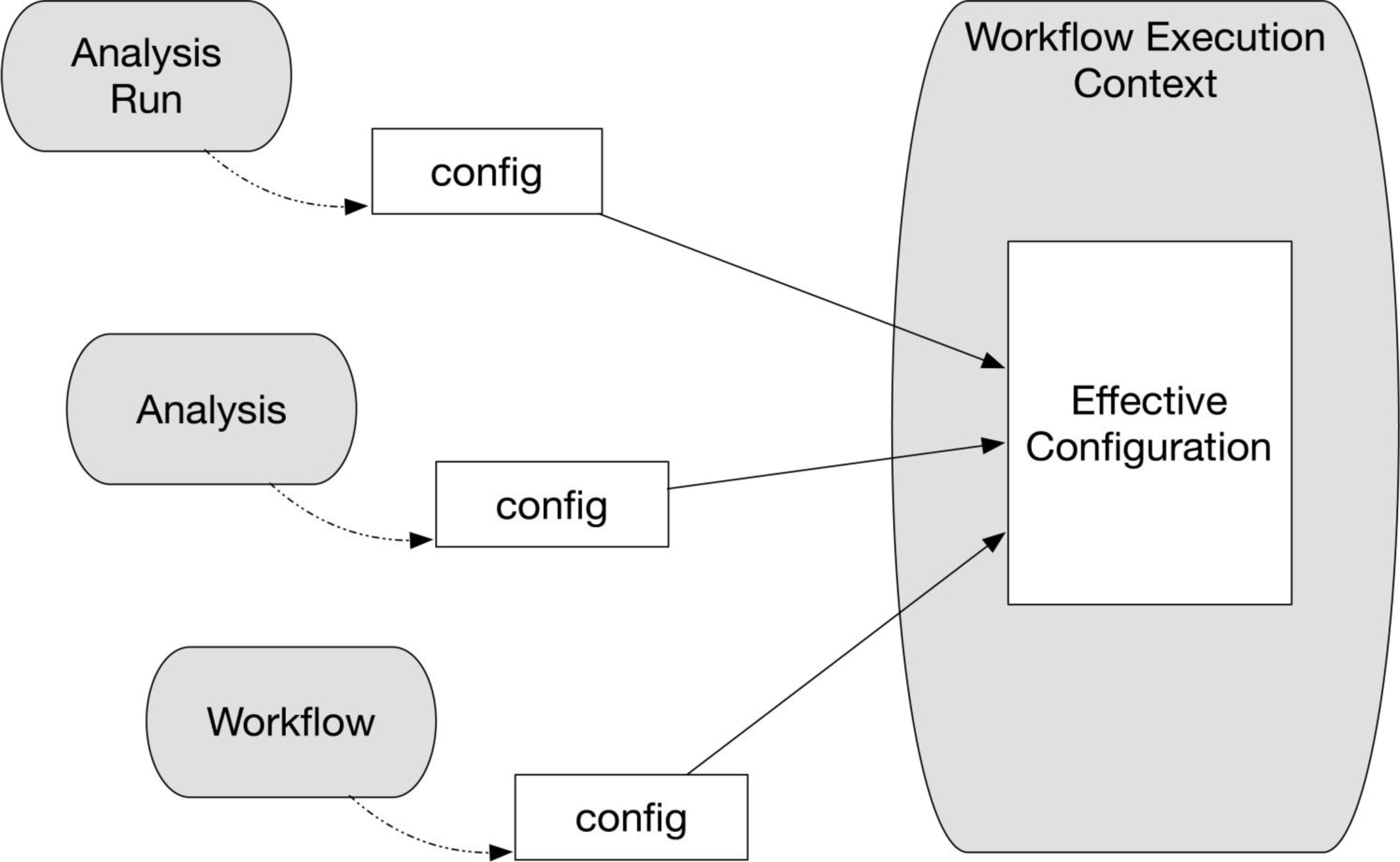

Supplement: Hierarchical tri-level configuration. — Configuration can be applied at three levels of granularity within Butler - Workflow, Analysis, and Analysis Run. Each higher level configuration may override and augment the configurations supplied at lower levels. At runtime all three levels of configuration are resolved into an “effective configuration”, which is then applied for execution. [file 41587_2019_360_Fig8_ESM.jpg]

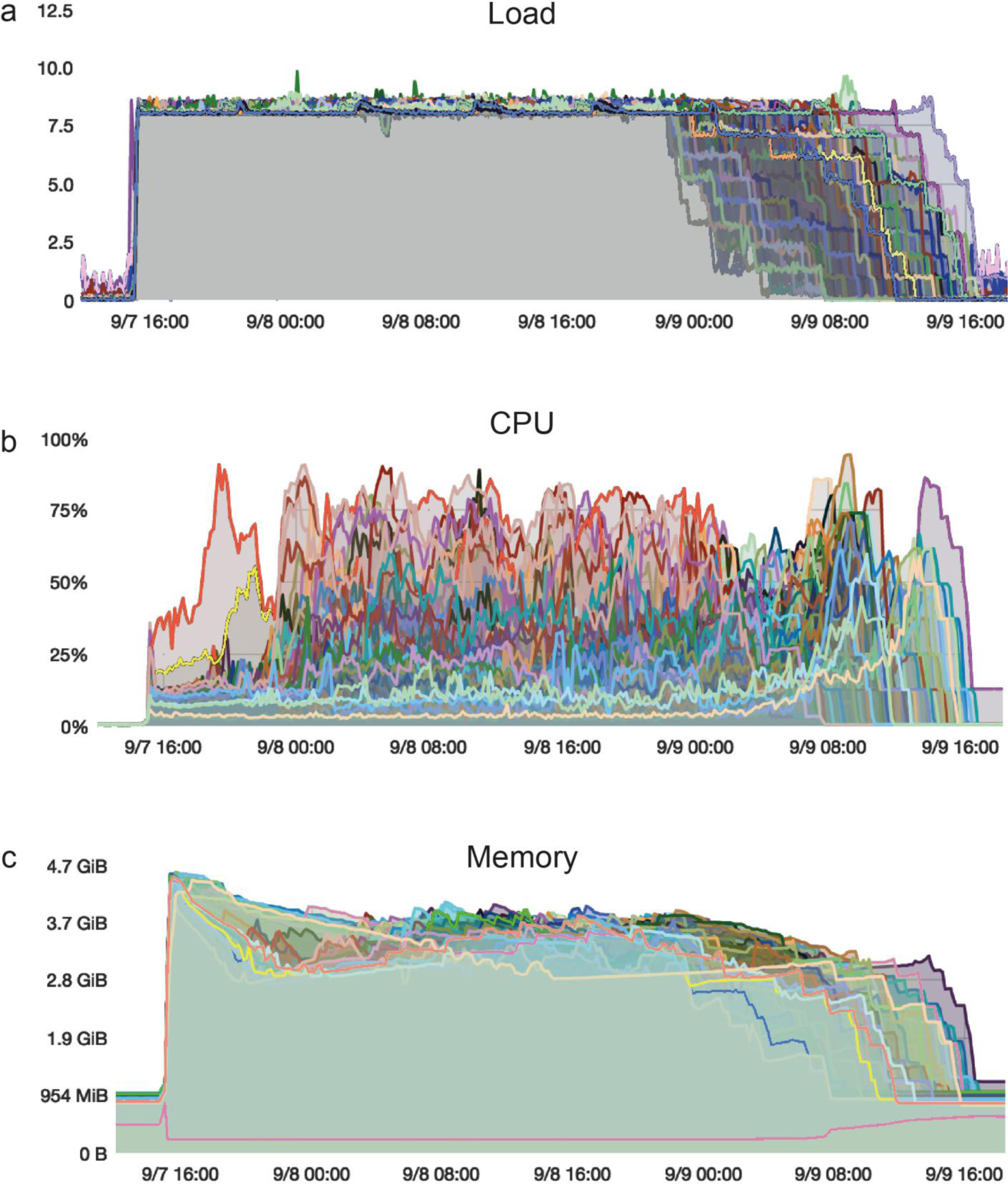

Supplement: Butler compute cluster performance metrics during germline deletion genotyping for PCAWG. — (a) Overall load per VM that is part of the Butler cluster - shows no load prior to analysis kick-off, then steady load throughout the analysis, and drop-off in load at the end when VMs start running out of work. (b) CPU profile shows highly variable CPU utilization that is typical of Delly executions. (c) Memory profile is stable and similar between all VMs that are running the analysis. Similar measurements have been observed over the other 5 analyses performed with Butler during PCAWG, although the exact pattern of CPU and Memory utilization is dependent on the algorithms that comprise the workflow being executed. [file 41587_2019_360_Fig9_ESM.jpg]

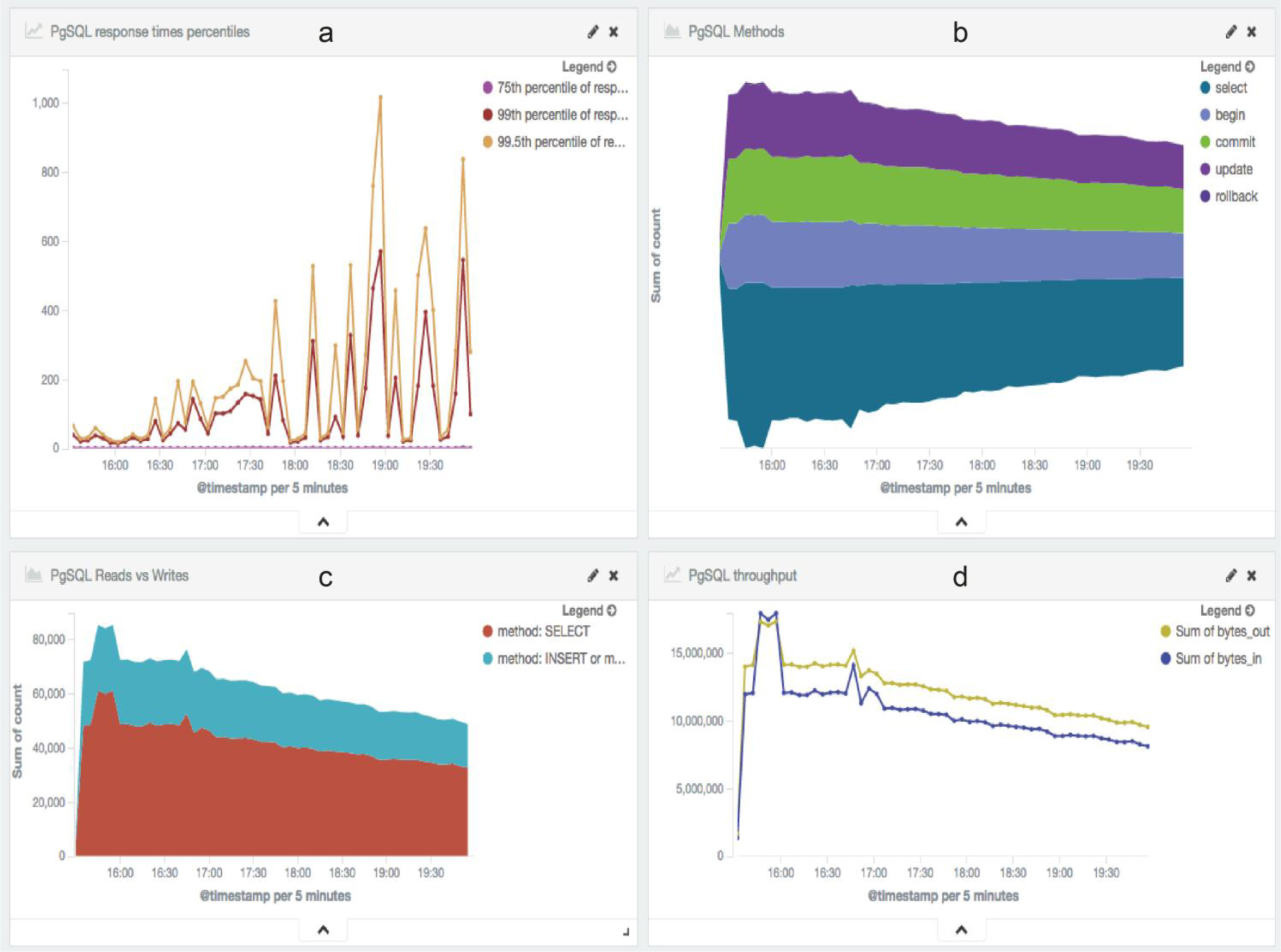

Supplement: SQL Database state monitoring dashboard. — SQL Database health can be ascertained from logs harvested on the database server. (a) 75th, 99th, and 99.5th percentile of query response times. (b) Count queries by type. (c) Database READ and WRITE counts. (d) Data throughput in and out. These measurements were collected over a single 2-hour run of the software and serve as an example of visualization capabilities, not an indication of typical database performance. [file 41587_2019_360_Fig10_ESM.jpg]
