# Supplementary material for: Butler enables rapid cloud-based analysis of thousands of human genomes
Source: Nat Biotechnol. 2020 Feb 5;38(3):288–92. doi: 10.1038/s41587-019-0360-3 (PMC7062635; doi:10.1038/s41587-019-0360-3)
Supplement: Supplementary file 9 — Supplementary Figures 1–8, Supplementary Tables 1–3 and Supplementary Note 1 [file 41587_2019_360_MOESM1_ESM.pdf]

nature portfolio

https://doi.org/10.1038/s41587-019-0360-3

Supplementary information

Butler enables rapid cloud-based analysis of thousands of human genomes

In the format provided by the authors and unedited

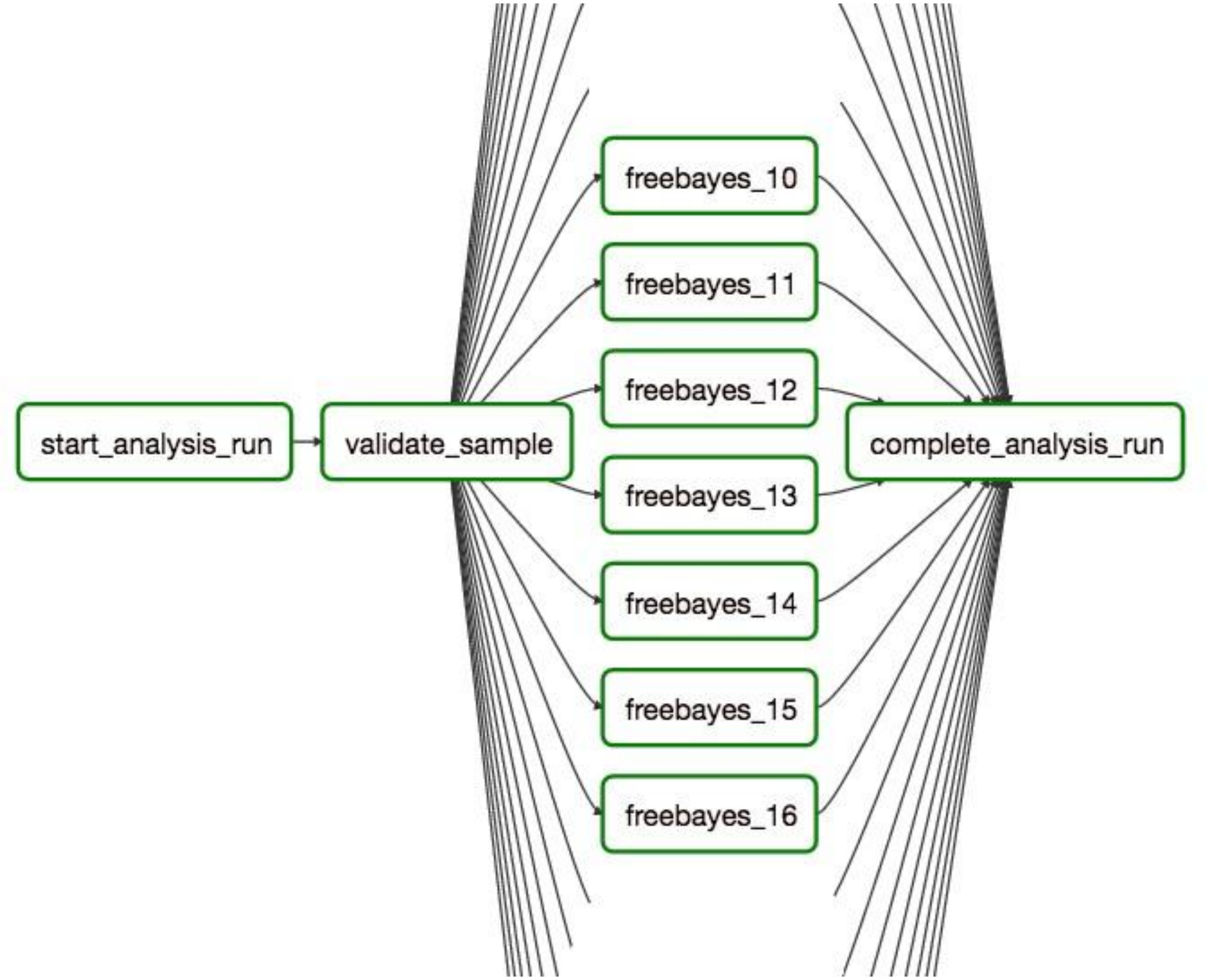

Supplementary Figure 1

Freebayes workflow

Freebayes workflow can be used for small variant discovery and genotyping and splits into tasks by chromosome, where each task can run in parallel (not all tasks are visible in figure to save space). Workflow is started and ended by standard start_analysis_run and end_analysis_run that keep track of Analysis state. validate_sample makes sure that access to the data is available.

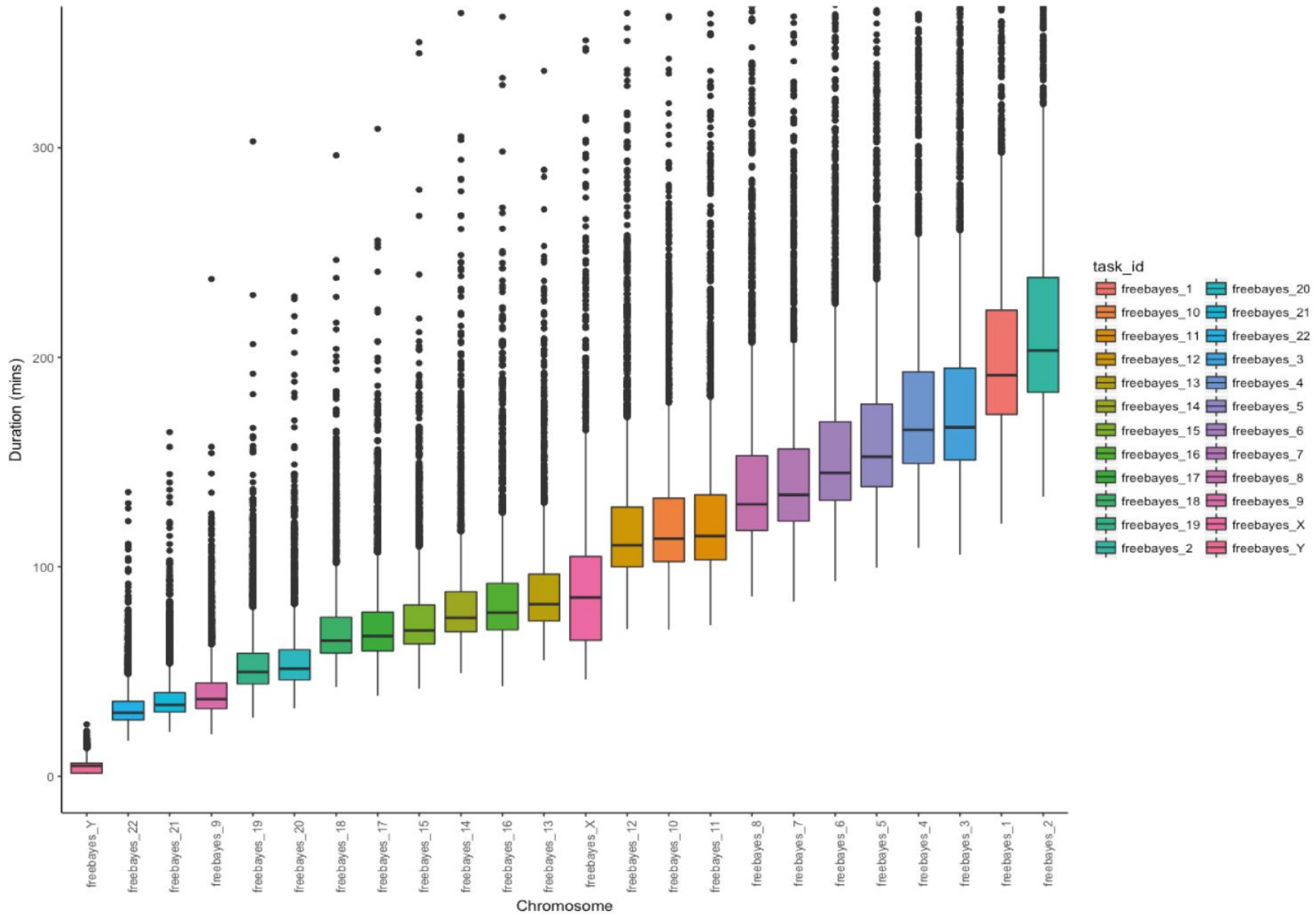

Supplementary Figure 2

Freebayes task durations

Boxplot of freebayes task durations during the SNV genotyping stage across 5668 samples. Durations are highly correlated with chromosome length (Pearson's r=0.92). n=5668 biologically independent samples Boxplot center line corresponds to the median, lower and upper hinges to the 25%th and 75%th percentiles, and whiskers to +- 1.5 Interquartile range from the hinges. The experiment was performed once.

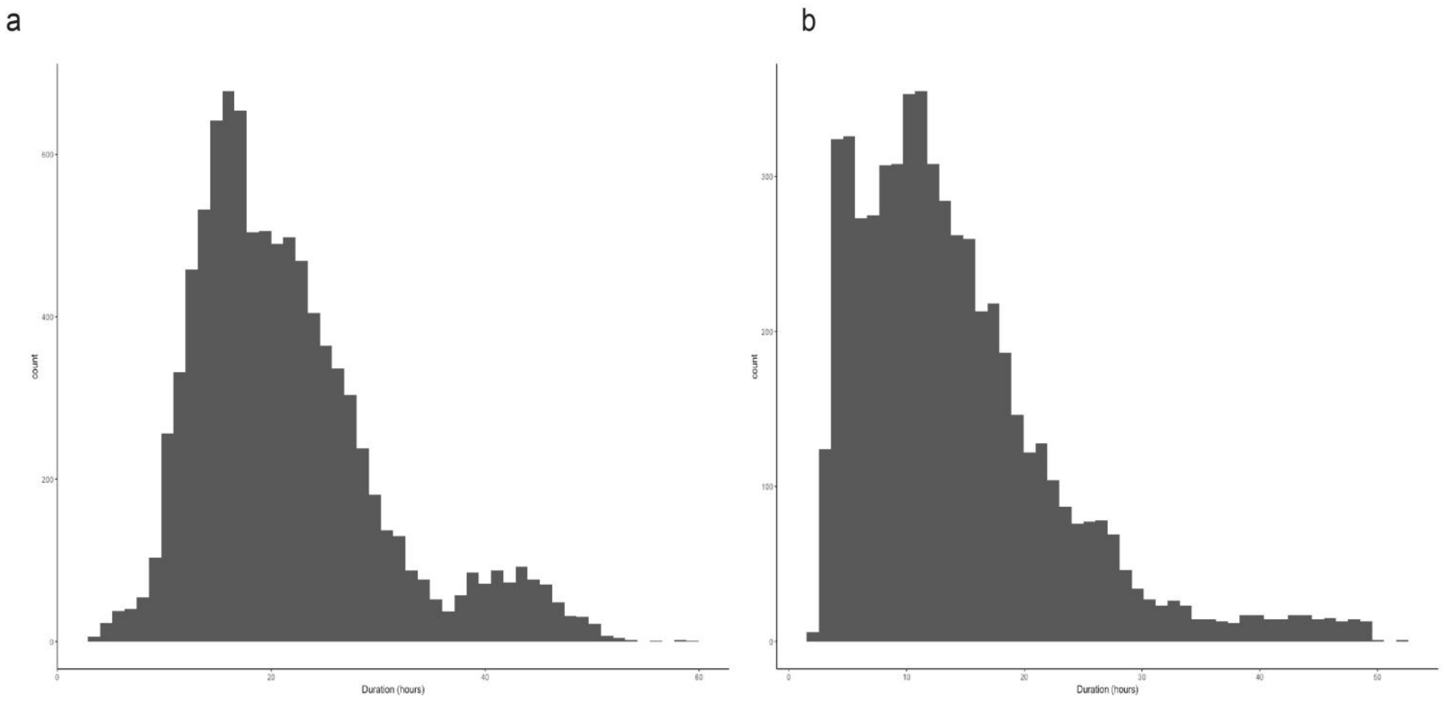

Supplementary Figure 3

Delly workflow durations

(a) Distribution of Delly workflow durations for genotyping of 244,889 germline deletions across 5668 PCAWG samples. (b) Distribution of Delly workflow durations for genotyping of 217,433 germline duplications across 5668 PCAWG samples. n=5668 biologically independent samples. The experiment was performed once.

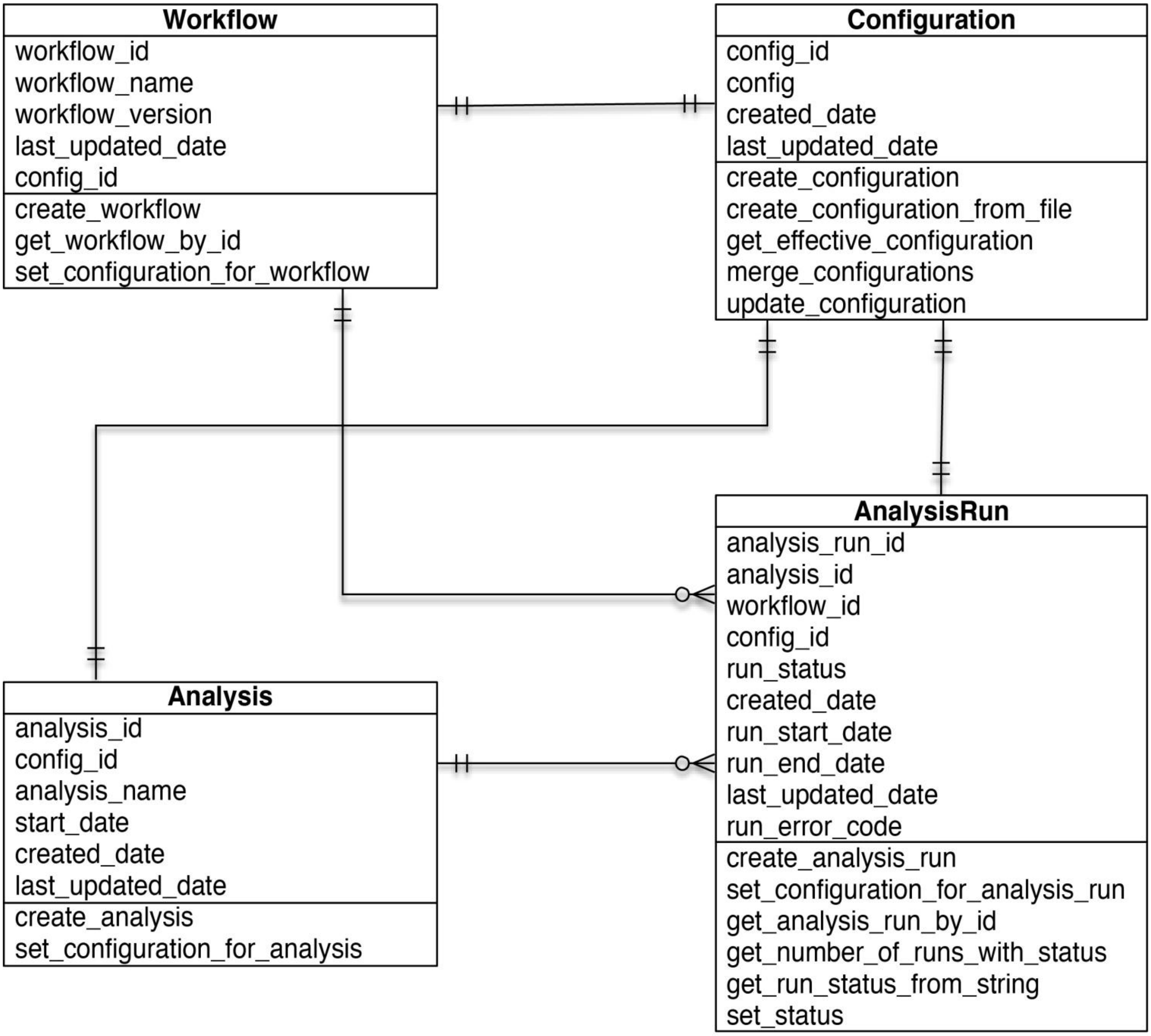

Supplementary Figure 4

Analysis Tracker UML diagram

The Analysis Tracker consists of four entities that are necessary for keeping track of the state of scientific analyses run in Butler. The Workflow object keeps a registry of known workflows and their attributes. The Analysis object keeps track of analyses that are being performed. An Analysis Run represents an instance of running a particular workflow under a particular analysis on a particular sample. Configuration objects keep track of the parameters supplied to the workflow invocation.

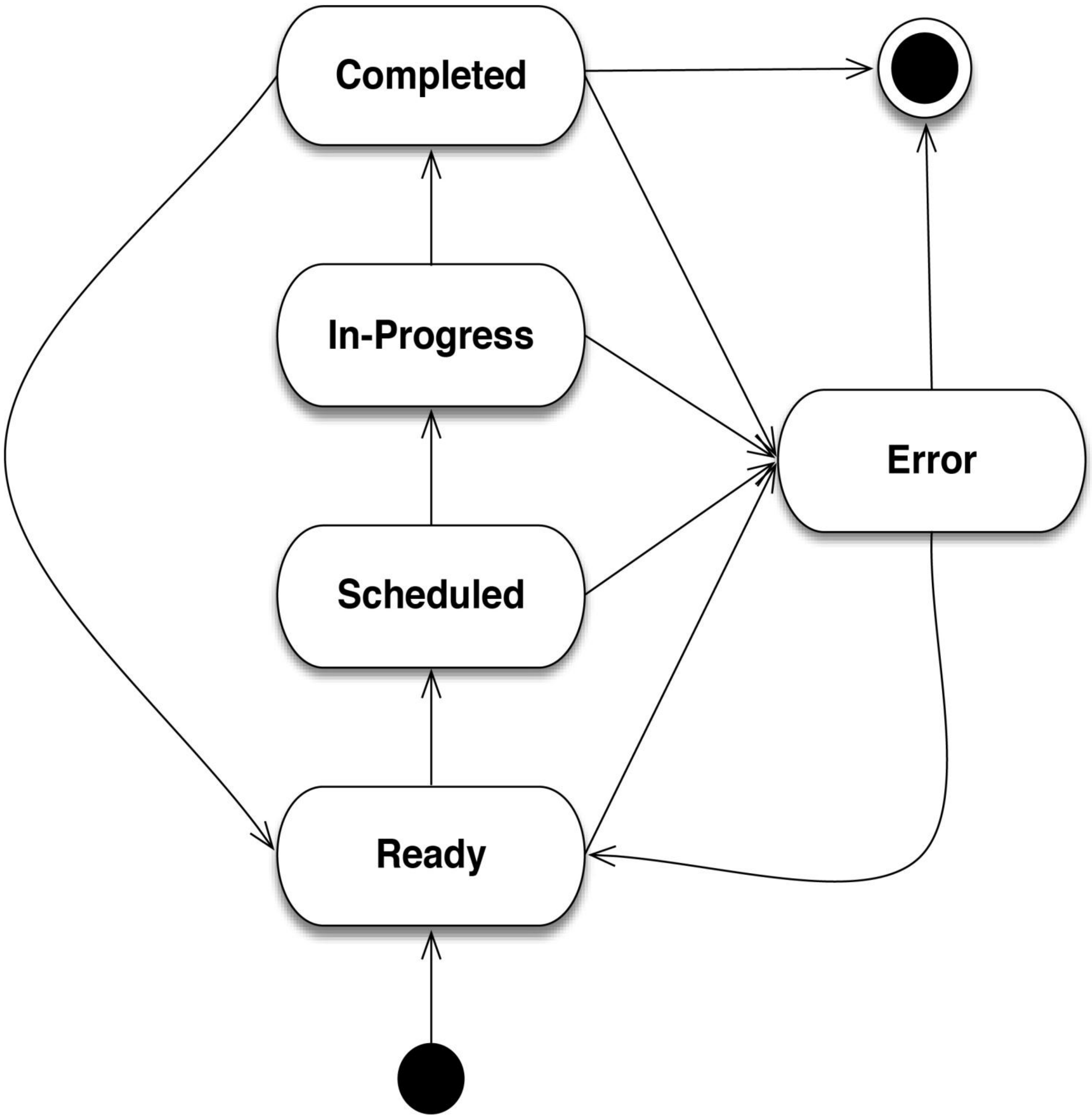

Supplementary Figure 5

Analysis Run state transitions

Each Analysis Run keeps track of its state and has a set of rules governing allowable state transitions. A Run is created in the Ready state from which it may be scheduled for execution. Once the corresponding workflow task is picked up for execution it is transitioned to In-Progress. Upon successful completion it is marked Completed. At any point a failure may put this run in an Error state from which it

can recover only to the Ready state to initiate a re-execution of the corresponding workflow.

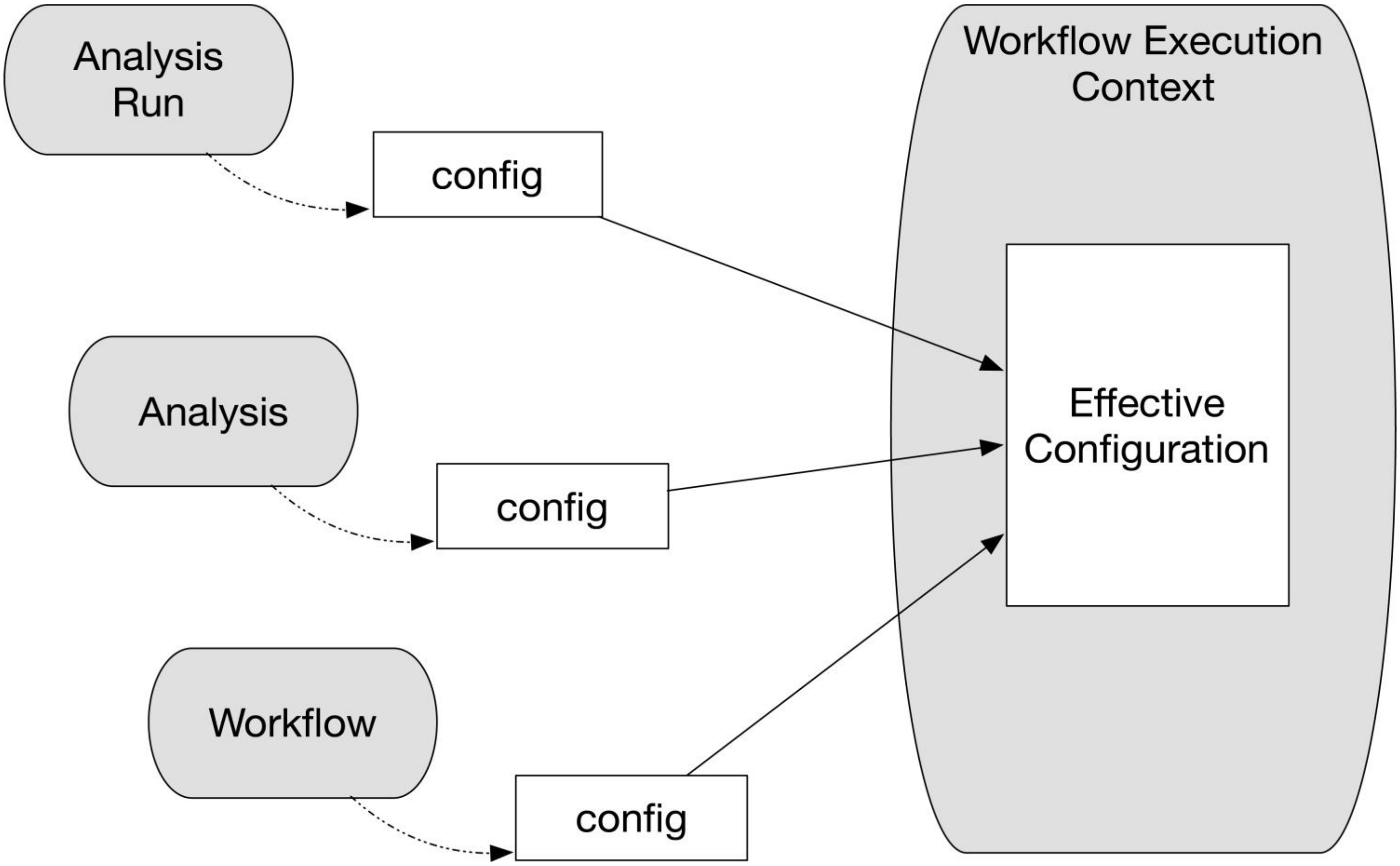

Supplementary Figure 6

Hierarchical tri-level configuration

Configuration can be applied at three levels of granularity within Butler - Workflow, Analysis, and Analysis Run. Each higher level configuration may override and augment the configurations supplied at lower levels. At runtime all three levels of configuration are resolved into an “effective configuration”, which is then applied for execution.

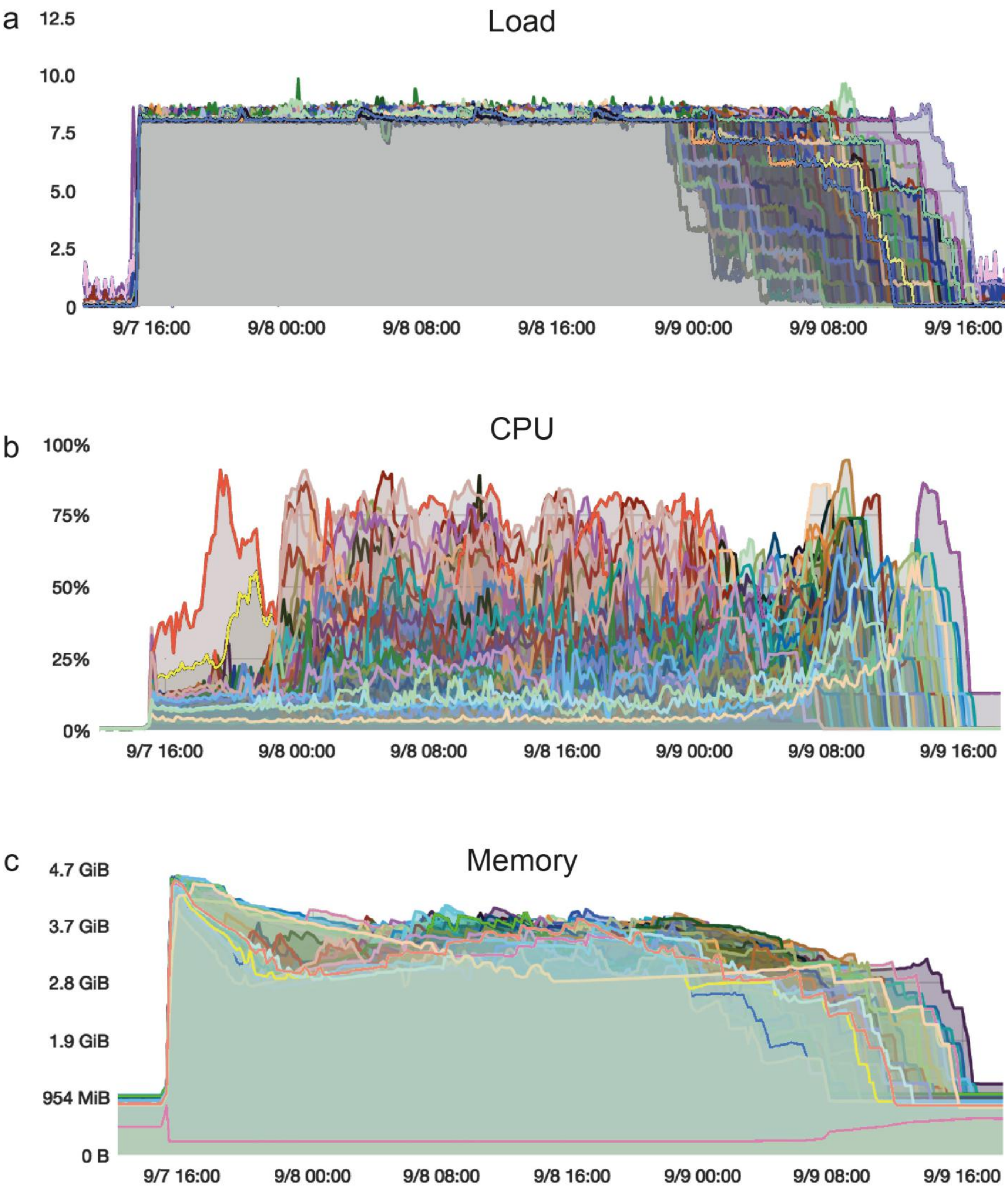

Supplementary Figure 7

Butler compute cluster performance metrics during germline deletion genotyping for PCAWG

(a) Overall load per VM that is part of the Butler cluster - shows no load prior to analysis kick-off, then steady load throughout the analysis, and drop-off in load at the end when VMs start running out of work. (b) CPU profile shows highly variable CPU utilization that is typical of Delly executions. (c) Memory profile is stable and similar between all VMs that are running the analysis. Similar measurements have been observed over the other 5 analyses performed with Butler during PCAWG, although the exact pattern of CPU and Memory utilization is dependent on the algorithms that comprise the workflow being executed.

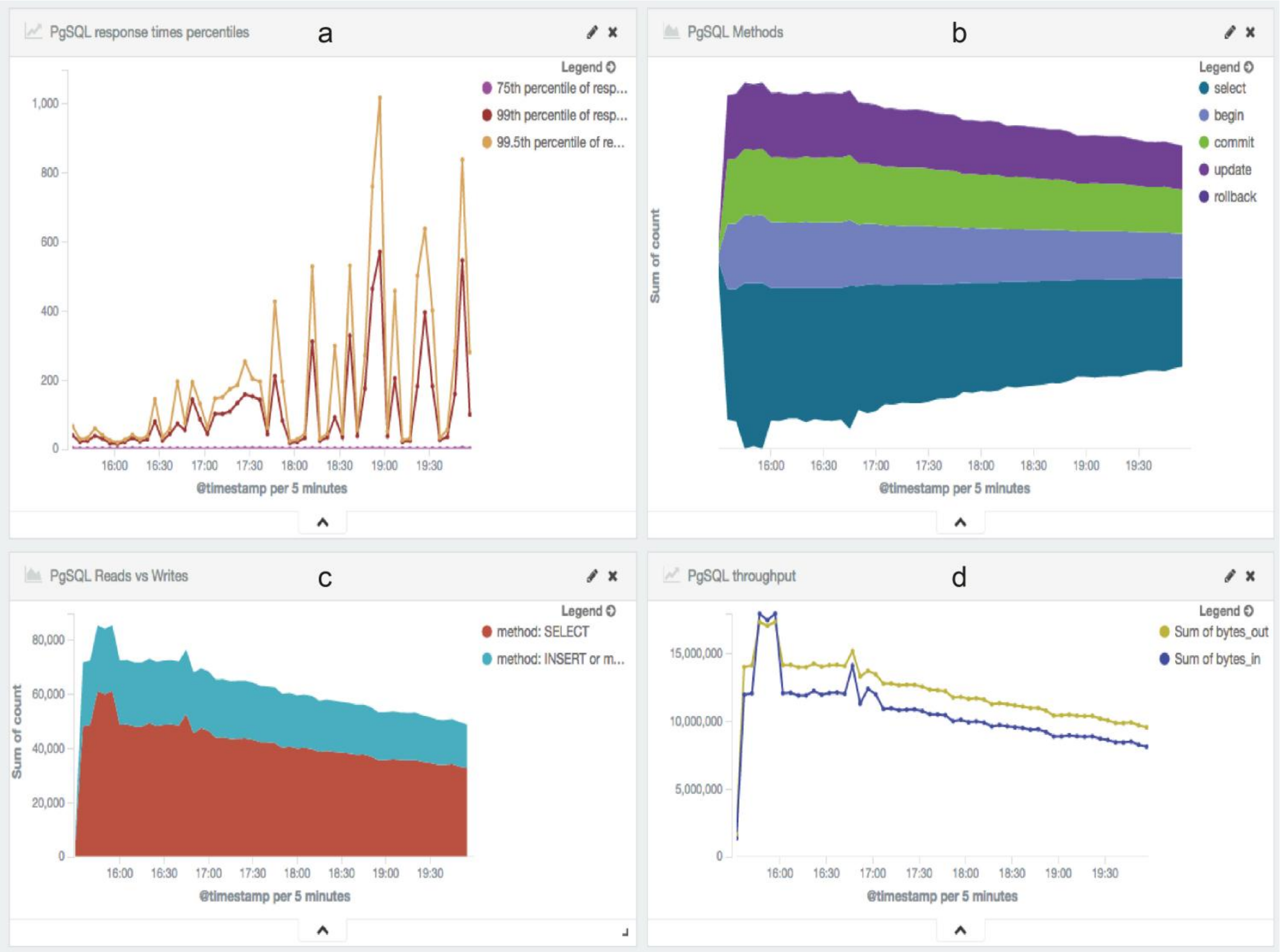

Supplementary Figure 8

SQL Database state monitoring dashboard

SQL Database health can be ascertained from logs harvested on the database server. (a) 75th, 99th, and 99.5th percentile of query response times. (b) Count queries by type. (c) Database READ and WRITE counts. (d) Data throughput in and out. These measurements were collected over a single 2-hour run of the software and serve as an example of visualization capabilities, not an indication of typical database performance.

Supplementary Tables

	PCAWG Tech	Butler	Toil	Nextflow	GenomeVIP
Language	Java	Python	Python	Groovy	PHP
Cloud platforms with full support*	3	4	1	1	1
Cloud platforms with partial support**	0	8	1	0	0
HPC support	No	No	Yes	Yes	Yes
Software Configuration Management	Yes	Yes	No	No	No
Networking and security	No	Yes	No	No	No
Health Metrics	No	Yes	No	No	No
Log Harvesting	No	Yes	No	No	No
Anomaly Detection	No	Yes	No	No	No
Multi-channel Alerting	No	Yes	No	No	No
Self-healing	No	Yes	No	No	No
Docker support	Yes	Yes	Yes	Yes	No
CWL support	Yes	Yes	Yes	No	No
Code sharing integration	Yes	Yes	No	Yes	No
Workflow modules	No	Yes	No	Yes	No
DAG Rendering	Yes	Yes	Yes	Yes	Yes
Distributed Cluster support (Spark, Mesos)	No	Yes	Yes	Yes	No

Supplementary Table 1 - Comparison of cloud-enabled scientific workflow frameworks. * full support is defined here to mean that all or most features of the framework are available on that cloud, including automated configuration, networking, security, storage, metrics, monitoring, and anomaly detection. **

partial support is defined to mean that only a partial implementation exists for a cloud, with fewer features, manual setup, or limited testing.

Machine	CPU	RAM(GB)	Disk(GB)	Roles	Count
salt-master	4	6	50 ephemeral<br>1000 block for metrics storage	salt-master<br>consul-bootstrap monitoring-server	1
tracker	4	4	40 ephemeral<br>1000 block for<br>elasticsearch	tracker<br>consul-server<br>elasticsearch	1
job-queue	4	4	40 ephemeral	job-queue<br>consul-client	1
db-server	8	16	80 ephemeral<br>1000 block for db	db-server<br>consul-client	1
worker	8	32	100 ephemeral	worker<br>germline<br>consul-client	175

Supplementary Table 2 - Butler deployment on EMBL-EBI Embassy Cloud

Core PCAWG	
BWA	0.16
Broad	0.14
Sanger	0.07
OxoG	0.14
Butler	
SNV Discovery	0.27
SNV Genotyping	0.90
SV Duplications	0.39
SV Deletions	0.28

Supplementary Table 3 - Optimal rates for PCAWG and Butler pipelines.

Supplementary Note 1

Below is a detailed description of the various sub-systems of Butler.

Cluster Lifecycle Management

This sub-system deals with the task of creating and tearing down clusters on various clouds, including defining VMs, storage devices, network topology, and network security rules. To fulfill these requirements in a cloud agnostic manner Butler utilizes an open-source framework called Terraform, developed by Hashicorp. Terraform uses a proprietary human- and machine-readable file format for specifying cluster configurations that is called HashiCorp Configuration Language (HCL). Using this language the end user can define a number of constructs for cluster management. The key task of Terraform is to perform Create, Read, Update, and Delete on cluster resources. Running Terraform causes the tool to inspect the current state and compare it to the target state, issuing any necessary commands to update current state to the target. Butler comes with a set of Terraform configuration files that define templates for all of the VMs that constitute a functional Butler cluster, as well as configurations for network security. A typical Butler cluster consists of Control VMs and Worker VMs and templates for both are available. The users are expected to adapt the templates as needed for their use case, providing their own credentials, cluster size, and other configurations.

Cluster Configuration Management

This sub-system deals with configuration and software installation of all VMs in the cluster. VMs typically will have hundreds of programs installed and configured on them, oftentimes with intricate interdependencies and inter-machine communication requirements. The Saltstack open-source Configuration Management system integrated in Butler allows managing these dependencies and installation details independently of the Operating System (OS) chosen for the Virtual Machines for deployments involving hundreds of servers (**Figure 1**). The Configuration Management System is controlled by a Master node that acts as the authority on the state of a cluster of Minion nodes. The Master has a set of configuration definitions defined and accessible through a git repository. Each Minion can have a number of roles assigned to it, and the Master maintains mappings between roles and configuration definitions. Once the Master has determined what roles a Minion has it can issue the necessary commands to apply relevant configurations to the Minion. Butler ships with configuration definitions required to run Butler itself as well as those needed to execute the bundled workflows (for example, sequence alignment with BWA, germline variant calling with freebayes and Delly, and somatic variant calling with Sanger Institute’s CGP tools). Additional configurations can be defined by the user as necessary.

Workflow System

The Workflow sub-system is responsible for allowing users to define and run scientific workflows on the cloud. Butler integrates the open-source distributed workflow system called Airflow, originally developed by Airbnb (www.airbnb.com), for this purpose. The key component at the heart of Airflow is the Airflow Scheduler (**Figure 1**). The airflow-scheduler is a service that runs perpetually on a VM and examines the state of all running workflows. All workflow tasks that meet the preconditions for being runnable are immediately "scheduled" for execution. In the context of Airflow scheduling means depositing the task into a queue (running on a separate Queue Server VM) from which a Worker VM (**Figure 1**) can eventually pick it up. The Worker VMs run an airflow-worker service that periodically polls the task queue for available tasks, and when the task is runnable by a particular Worker, that Worker consumes the task message from the queue and assumes execution. In order to keep track of the status of Workers and workflow execution each Worker periodically sends heartbeat messages to the Scheduler to communicate its state. The state is persisted by the Scheduler to a PostgreSQL database, which runs on a DB Server VM (**Figure 1**).

The user can communicate with and commandeer Airflow via the Airflow CLI, as well as a Web UI. The Web UI is provided via the airflow-flower, and airflow-webserver services, which can run on the same VM as the Scheduler or on a separate VM, depending on system load. Conceptually, an Airflow workflow is a Directed Acyclic Graph whose vertices represent tasks and edges indicate task sequence. In its implementation, an Airflow workflow is a Python program that can use any Python language construct or library. This allows the users to create workflows of arbitrary complexity and functionality.

An Analysis Tracker module is built into Butler in order to allow the user to define analyses, specify what workflows are part of these analyses, and track the status and execution of Analysis Runs - instances of running a particular workflow on a particular data sample within the context of an Analysis (**Supplementary Figure 4**).

When an Analysis Run is first created it is given a Ready status (**Supplementary Figure 5**), indicating that it is ready to be scheduled for execution. Once the Scheduler has scheduled the Run for execution it is given a Scheduled status. When workflow execution starts the Run is marked In-Progress. Once the Run is successfully completed it enters a Completed status. If, at any point, the Run encounters an error condition it cannot recover from, the Run Status is set to Error. When the error condition is addressed the Run status should be set to Ready so that it can start from the beginning.

In order to fulfill the workflow configuration and parametrisation requirements Butler implements a tri-level configuration mechanism (**Supplementary Figure 6**), allowing the user to specify configurations at Workflow, Analysis, and Analysis Run levels. At runtime all three configuration levels are merged into one “effective” configuration that applies within the execution context. Because it is important for configurations to be both human-readable and machine-readable Butler uses the JSON format to encode

configuration information. PostgreSQL, in turn, has native support for storage and deep querying of JSON values, thus making it an ideal choice for configuration persistence.

Operational Management

This sub-system provides tools for ensuring continuous successful operation of the cluster, as well as for troubleshooting error conditions. In general, the Operational Management tools fall into two categories, those that collect observations about the state of each component in the system at runtime, and those that aggregate this data and present it to the user in the form of queryable databases and management reports. We delineate two major sources of data that are indicative of system state - System Metrics, and Server Logs. While metrics provide more of a coarse-grained view of the overall health of a particular Virtual Machine, server logs can give much more of a fine-grained view of the underlying system at an application level, and down to individual lines of code that are running at any given time. Butler has dedicated components for the collection and management of these data sets

Each VM runs a metric collection daemon called Telegraf, which is an open-source package that is able to make periodic measurements of a large number of system metrics and ship them off to a centralized Monitoring Server. The definition for which metrics are collected is specified in a special configuration file. Because we are interested in observing not only the metrics as they are measured in the present, but also the dynamics of how metric values change over time, we need a mechanism for persisting this information. For this purpose the Monitoring Server component of Butler contains an instance of a database product called InfluxDB, which is an Open Source database system that is optimized for recording time series data.

The metrics collection system is collecting 50 different metrics per host on average, sampled at intervals of 10 seconds. Given a cluster of 200 Virtual Machines the monitoring system collects and stores 86,400,000 data points in a 24 hour time period. This volume of data is quite difficult for the user to comprehend and make use of, and Butler provides visualization tools to enable the display of aggregate statistics based on the monitoring data using a Graphical User Interface (**Supplementary Figure 7**). The main goal of the visualizations is to give the user an overview of the trends observed within the compute cluster with respect to a set of representative performance metrics, and to alert the user to any conditions that threaten the health of Virtual Machines and the scientific analyses they run.

Because of the potentially extremely high value of the information contained in server logs, we deploy a system of log harvesting and centralized storage that enables the Virtual Machines that are part of Butler to parse the logs that are being generated locally for interesting events and send those events to a centralized search index which is amenable to efficient querying and visualization. These open-source tools are known as the ELK stack (Elasticsearch, Logstash, Kibana).

Each Virtual Machine in the cluster runs a log shipper - Filebeat. It is responsible for finding, harvesting, and locally aggregating logs. Logstash runs on a separate centralized server and is responsible for parsing the logs forwarded from Filebeat and sending the parsed information on to the Elasticsearch index. Elasticsearch is a general-purpose scalable text indexing and search engine that supports clustering and sharing of data. Just as it is difficult to grasp and analyze performance metrics due to the number of data-points generated, it is as difficult to grasp log messages from a large cluster. We utilize a similar set of visualization tools to the ones we use for metrics, to solve this problem for server logs within Butler. The Kibana dashboarding framework allows us to create graphical dashboards that visualize log events of interest, as well as providing a web-based query interface to the Elasticsearch log messages index (**Supplementary Figure 8**).

The Monitoring Server runs an anomaly detection and alerting library called Kapacitor, which defines a series of rules that specify the normal operating conditions for the cluster, such as all hosts regularly responding to ping, CPU below 80%, disk utilization below 85%, workflow tasks and other Butler services sending heartbeats every minute. Coupled with the application metrics gatherer *statsd*, the system builds an empirical distribution of the duration of various workflow tasks and knows when tasks take longer than they historically have. The anomaly detection software monitors the time series database that records all metrics and periodically evaluates the rule-set against it. When the system detects a breach of one or more rules it can take a remedial action, such as sending a warning email, a message to a Slack topic, a Telegram, or schedule the restart of a particular service, workflow or the reprovisioning of a particular VM (**Figure 1 b**). These abilities allow Butler users to be always up to date about the health of the system and allow Butler clusters to self-heal when they encounter problematic scenarios.

This approach is markedly different, and offers dramatic improvements, over error handling routines implemented in other workflow frameworks. Nextflow, for instance, provides a workflow *onError* event handler and an *errorStrategy* (https://www.nextflow.io/blog/2016/error-recovery-and-automatic-resources-management.html); these allow for simple retry logic to be set up including the ability to request more resources (such as memory) during the retries, and possibly notify users via email – but to trigger this routine requires that a workflow can signal the error, which in practice will often not be the case. Toil, similarly, allows for job retries upon failure (http://toil.readthedocs.io/en/releases-3.6.x/running/running.html#error-handling-and-resuming-pipelines), again requiring that workflows are able to report crashes. These routines, therefore, are predicated on the workflows actually failing and being able to report that. In practice, workflow failure may not happen right away, may not happen at all, or may not be successfully reported, for example when systemic IT infrastructure issues are the root cause of failure – which in practice leaves significant gaps and is the cause of some of the hardest to detect, and costly project issues. Additionally, before the underlying issue is resolved, no amount of retrying will cause the workflow to succeed, ensuring that the workflow retry logic on its own has limited utility in practice.

For example, consider a scenario where the job scheduler component crashes or stops responding. This does not cause any jobs to fail because they are handled by the worker nodes. Instead, new workflows simply fail to get scheduled, causing the system to slowly run out of work and become idle. In a large deployment with hundreds of VMs this condition quickly becomes costly, yet may go undetected because no workflows are failing. Butler deals with these situations easily, since it collects heartbeats from all essential services. As soon as the heartbeat is missing for a pre-determined period of time an alert is raised. Butler's self-healing agent will communicate with Butler's Configuration Server using its HTTP API and will instruct the Configuration Server to restart the Workflow Scheduler (**Figure 1 b**). If it is not able to bring the scheduler back online automatically it will alert users via email and Slack notifications.

It is important to understand that even though Butler provides the anomaly-detection and self-healing framework along with some preconfigured rules and actions, the types of rules and actions that it makes sense to deploy at a given time is highly dependent on the context of the analysis being performed and the IT infrastructure that the system is deployed on. Thus, users should expect to spend effort working within this framework to properly set up and tune the rules that will best serve their needs. For example, if users set up a rule of the form "Automatically reschedule workflow tasks that have taken above 3 standard deviations of the mean runtime and have not yet completed" and train this rule on a workflow that analyses small bacterial genomes, but then decide to deploy it on an organism with a much larger genome, this rule may trigger unnecessarily given marked differences in expected and acceptable runtimes. While high levels of automation can be extremely powerful and save time and costs when used in the right context, they may also wreak havoc when ill-configured.

There are in general three types of situations that the anomaly detection and self-healing components can go after:

The most high-level scenario involves detecting and resolving when an entire VM becomes stuck or otherwise unresponsive. Detection is accomplished via an *uptime* metric, which is collected for all hosts and measures how long each host has been up. If this metric is not updated for a long time the VM can be considered "dead". This is fairly easy to deal with when a VM is a worker node; a new node can simply be launched using Butler's existing provisioning functionality. It is more difficult to replace one of the management nodes in an automated fashion due to the need to rescue and migrate existing services like databases and queues. Thus, it is recommended to run these services with standby backups if high availability is desired and manually replace the nodes that go out of commission.

At times, one of the individual services that Butler relies upon for its operation will crash. The greatest majority of these can be solved by a service restart. Butler collects a heartbeat from all such services and when the heartbeat is not detected will attempt to restart the offending service via the Configuration Server (**Figure 1 b**). As Butler collects detailed statistics about each service it can potentially act on some other conditions that may affect a service (such as running out of disk space) in an automated fashion. There

are scenarios, however, such as network outages, which are impossible to fix automatically; if Butler is not able to bring the service back online after several tries it will notify a human via email and Slack notifications to ensure the issue will be resolved through human intervention, in a timely manner.

The most advanced and fine-grained application of anomaly-detection is instrumenting individual workflows with metrics using the *statsd* library in order to collect timings, CPU, memory, and disk footprint. This allows the finest degree of automated control over workflow execution letting the system operator to detect workflows that are not crashing, but are not likely to ever finish because of a bug in the software or a data issue. Setting appropriate detection thresholds for these types of events requires detailed understanding of the resource requirements and usage patterns of the underlying algorithms that are being implemented by the workflow. Usage of these features is highly recommended for large-scale, long-running projects (*e.g.* studies at the scale of PCAWG or PPCG) or for ongoing production operations with a high degree of tuning.

Although the anomaly-detection and self-healing capabilities of Butler are extremely powerful, they also have some remaining limitations. Since these components themselves are not immune to failure, adverse events that impact their ability to function will inevitably prevent the system from being able to adequately detect and respond to other failures. This includes failures of individual metrics collection agents, the time-series database, the rules engine, and the configuration server, as well as system-wide events like network outages that prevent VMs from being able to communicate with each other. In this regard the time series database and the rules engine stand out as single points of failure for the entire self-healing functionality while the failure of other components will only have a limited effect. These key components, however, can be deployed in cluster mode with standby backups to mitigate the risk of such events. One additional source of issues that is difficult for the self-healing functionality to address is the class of errors caused by data issues. Such issues are virtually impossible to deal with generically, and require a degree of expert understanding of the underlying data sets and data formats that we would advise against implementing at this level of a workflow system. Instead, we would recommend implementing the data quality assurance steps as a separate workflow that should run over the samples before they undergo further analysis.

The anomaly detection and self-healing capabilities of Butler implement a host-, service-, and workflow-level quality assurance framework that dramatically increases system robustness when operating at large scale. It should be used with care, however, to not over-automate, inadvertently introducing false-positive signals, in order for the users to reap the most benefit from this powerful functionality.

The Butler framework consists of many different services that reside on a number of different servers and need to be able to communicate with each other. To accomplish this in a flexible manner we needed to establish a Service Registry so that IP addresses of servers that host particular services can be looked up by service name. To accomplish this, Butler uses an open-source service discovery framework called Consul. Consul provides a cross-data-center distributed Service Name Registry that is available via

HTTP and DNS protocols. In addition to registry capabilities Consul provides basic health checks for the underlying services, testing whether the IP and port the service is supposed to be listening on are actually reachable.
